# Pathway Complexity Defines the Shape of Nucleic Acid‐Loaded Polyion Complex Micelles: Spheroids vs. Hexagonal Disks

**DOI:** 10.1002/anie.9345533

**Published:** 2026-09-10

**Authors:** Lennart van den Hoven, Neshat Moslehi, Eline F. Brink, Austin Hubley, Ilja K. Voets, Tina Vermonden, Elham Mirzahossein, Bas G. P. van Ravensteijn

**Affiliations:** ^1^ Division of Pharmaceutics Utrecht Institute for Pharmaceutical Sciences Faculty of Science Utrecht University Utrecht The Netherlands; ^2^ Laboratory of Self‐Organizing Soft Matter Department of Chemical Engineering and Chemistry & Institute For Complex Molecular Systems Eindhoven University of Technology Eindhoven The Netherlands; ^3^ European Synchrotron Radiation Facility (ESRF) Grenoble France

**Keywords:** complex core coacervate micelle, cryogenic transmission electron microscopy, DNA, fluorescence correlation spectroscopy, hexagon, polyplex, polyelectrolytes, Polymerization‐induced electrostatic self‐assembly (PIESA), time‐resolved small‐angle X‐ray scattering

## Abstract

(Macro)molecular self‐assembling systems are typically subjected to pathway complexity. The final structural outcome is not only determined by the system's composition but also depends on the details and kinetics of the pathway chosen to navigate the energetic landscape during the assembly process. In this study, a distinct manifestation of pathway complexity is presented in the formation of short double‐stranded DNA (dsDNA)‐loaded polyion complex (PIC) micelles. Besides the conventional assembly pathway of rapidly mixing dsDNA with preformed polycationic block copolymers, compositionally equivalent PIC micelles are prepared via polymerization‐induced electrostatic self‐assembly (PIESA). A combination of synchrotron time‐resolved small‐angle x‐ray scattering (TR‐SAXS), cryogenic transmission electron microscopy (cryo‐TEM), and fluorescence correlation spectroscopy (FCS) reveals the formation of spheroidal particles during direct mixing, whereas PIESA generates hexagonal disk‐shaped colloids with internal crystalline dsDNA ordering. A molecular assembly mechanism for PIESA is postulated, which relies on the continuous formation of charge‐neutralized complexes that act as primary building blocks for the colloidal hexagons. Mechanistic insights into pathway complexity during PIC micellization establish assembly pathway control as a strategy to tune the particle's physicochemical properties beyond conventional design parameters, such as polymer size and chemistry. This approache expands the opportunities for therapeutic oligonucleotide delivery through control over morphology and cargo loading.

## Graphical abstract

Same building blocks, different destinies. Pathway complexity directs nucleic acid–loaded polyion complex micelles along distinct assembly trajectories, resulting in either hexagonally shaped or spheroidal nanoparticles with distinct internal structuring. Their divergent formation pathways are captured in real time through combining synchrotron time‐resolved small‐angle x‐ray scattering (SAXS), cryogenic transmission electron microscopy (cryo‐TEM), and fluorescence correlation spectroscopy.

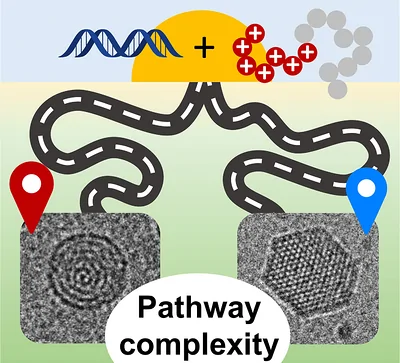

## 1 Introduction

Polyion complex (PIC) micelles are polyelectrolyte‐based nanostructures formed via associative liquid–liquid phase separation of oppositely charged macromolecules, yielding a charge‐compensated coacervate core stabilized by a solvated neutral corona [1, 2, 3]. Their formation is driven by electrostatic interactions and concomitant counterion release, while steric stabilization imposed by neutral polymer chain segments confines the assembly process to the nanoscale [2, 4]. Owing to the inherently dynamic nature of their highly hydrated polyion cores and their sensitivity to environmental conditions (e.g., ionic strength, pH, and charge stoichiometry), PIC micelles have emerged as versatile platforms for the (reversible) encapsulation and delivery of sensitive (bio)molecular cargo [5, 6, 7, 8, 9, 10, 11, 12, 13, 14].

In particular, their use as nanocarriers for nucleic acid (NA) payloads has been extensively investigated [15, 16, 17]. Encapsulation of NAs enhances cargo stability, prolongs circulation, improves cellular uptake, and reduces systemic toxicity, ultimately enabling more efficient and targeted therapeutic delivery while preserving biological activity [18]. Despite impressive advances in various in vitro and model in vivo applications [19, 20, 21], PIC micelles and related delivery systems have demonstrated limited translational success. It has been hypothesized that these limitations arise, at least in part, from the challenges associated with precisely controlling key physicochemical properties, including particle size, shape, and cargo loading, which govern biodistribution, circulation time, cargo release kinetics, toxicity, and ultimately therapeutic efficacy [22, 23].

Underlying this lack of structural control are competing assembly pathways that occur upon mixing of the oppositely charged macromolecular components. Assembly either proceed through non‐equilibrated aggregates that subsequently relax into micelles or through stepwise growth from small unit polyion complexes (uPICs) [24, 25, 26, 27, 28]. These pathways are highly sensitive to system chemistry and assembly conditions, and both are susceptible to kinetic trapping [3, 29]. As a result, experimentally observed structures often deviate from thermodynamic predictions [30], necessitating post‐assembly annealing [9, 26]. To overcome these limitations, polymerization‐induced electrostatic self‐assembly (PIESA), in which one polyelectrolyte is generated in situ using its oppositely charged counterpart as a template was coined as alternative assembly pathway [31, 32, 33, 34, 35, 36, 37]. This approach enables a gradual buildup of electrostatic interactions governed by the polymerization kinetics, thereby mitigating kinetic trapping and providing enhanced control over PIC micelle formation.

Here, we mechanistically track the assembly kinetics and resulting structures of NA‐loaded PIC micelles prepared by PIESA and conventional direct mixing, using synchrotron‐based time‐resolved small‐angle x‐ray scattering experiments (TR‐SAXS) as well as cryogenic‐transmission electron microscopy (cryo‐TEM). Facilitated by the relative rigidity of the employed double‐stranded DNA (dsDNA), a model cargo for therapeutically relevant nucleic acids such as siRNA, significant differences in particle morphology, internal structure, and NA loading were observed. Based on time‐dependent fluorescence correlation spectroscopy (FCS) measurements, a molecular formation mechanism was formulated to corroborate the mesoscale information obtained from TR‐SAXS and rationalize the profound manifestation of pathway complexity.

## 2 Results and Discussion

### 2.1 Formation Pathways: Same Chemistry; Different Outcome

We selected an experimental system (Figure 1a) consisting of short double‐stranded DNA (dsDNA, see Section S1.1 for chemical details and sequence) as permanently charged polyanionic template and a block copolymer (BCP) carrying a charge‐neutral poly(*N*,*N*‐dimethylacrylamide) (pDMAc) and polycationic poly(3‐acrylamidopropyl)trimethylammonium chloride) (pAPTAC) segment. APTAC was selected because its quaternary ammonium group is covalently linked through a stable amide bond, thereby ensuring a constant charge ratio with the dsDNA template throughout all PIC formation experiments. For the conventional direct mixing assembly pathway, aqueous solutions of pre‐synthesized p(DMAc_119_‐*b*‐APTAC_50_) (*M*_n, DM_ = 11.4 kg/mol, *Ð* = 1.13, Figures S2 and S4) and dsDNA (44 anionic charges) were mixed at the point of charge stoichiometry ([+] = [−] = 5 mM, see Section S1.6). p(DMAc‐*b*‐APTAC) was obtained by chain‐extending a p(DMAc_119_) macro‐chain transfer agent (mCTA) (*M*_n_ = 5.7 kg/mol, *Ð* = 1.19, see Section S1.2 and Figures S2‐S4) with APTAC using photo‐induced reversible addition‐fragmentation chain transfer (RAFT) polymerization with lithium phenyl‐2, 4, 6‐trimethylbenzoylphosphinate (LAP) as light sensitive radical source (*λ*_max, illumination_ = 365 nm, see Section S1.4). Near quantitative monomer conversion was accompanied with excellent chain extension efficiency as observed by size exclusion chromatography (SEC, Figure 1b, gray *vs*. red curve). Equivalent PIC micelles were prepared by performing the same photo‐RAFT synthesis of p(DMAc‐*b*‐APTAC) but in the presence of the dsDNA template. The use of photo‐initiation under low‐intensity irradiation and short exposure times ensured compatibility with the template [38, 39, 40]. Similar to the direct mixing procedure, a stoichiometric charge ratio of 1 was used where the positive charges now originate from the APTAC in the initial reaction mixture ([+] = [−] = 5 mM; see Section S1.5). After initiating polymerization, monomer was converted relatively rapidly to a final conversion of 85% as probed with ^1^H‐NMR (see Figure S5). Faint turbidity was observed, a hallmark for particle formation. Achieving rapid monomer consumption is consistent with the PIESA mechanism where the oppositely charged monomer presumably accumulates in the close vicinity of the template, thereby creating a local high monomer concentration and thus fast polymerization kinetics despite the overall low monomer concentration in the system. The plateau in APTAC conversion at approximately 85% is attributed to multivalent binding of the growing polymer to the template, which displaces the monomer into the bulk phase and reduces its local concentration below that required for further polymerization [33].

**FIGURE 1:**
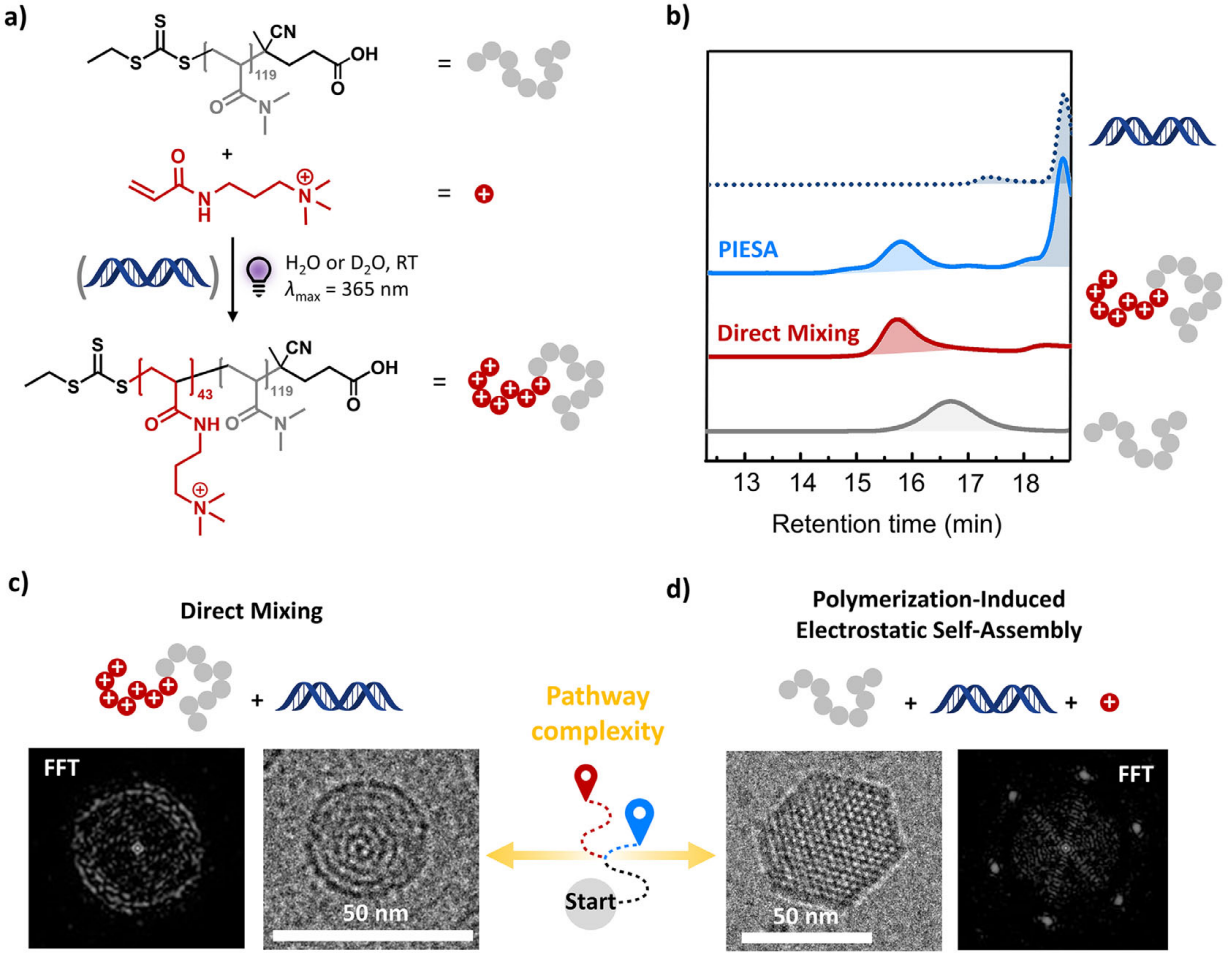
(a) Synthesis of p(DMAc_119_‐*b*‐APTAC_43_) via photo‐initiated reversible addition‐fragmentation chain transfer (RAFT) polymerization. The resulting block copolymer was either isolated, purified, and used for polyion complex (PIC) micelle formation via direct mixing or synthesized in the presence of polyanionic double‐stranded DNA (dsDNA) templates to yield PIESA‐derived PIC micelles. (b) Size exclusion chromatography (SEC) traces of employed p(DMAc) macro‐chain transfer agent (gray), p(DMAc_119_‐*b*‐APTAC_43_) synthesized in the absence and presence of the dsDNA template (red and blue, respectively) and the template (dark blue, dotted line). An aqueous 0.25 M ammonium acetate buffer (pH = 5.5) + 10%v/v DMF was used as eluent and eluting species were detected using a differential refractive index (dRI) detector. Cryo‐TEM images and corresponding Fast Fourier Transforms (FFT) of (c) direct mixing and (d) PIESA‐derived nanoparticles. Scale bar = 50 nm.

Disassembling the particles by increasing the ionic strength (300 mM, see Figure S7) and analyzing the formed BCPs with SEC revealed a near‐identical molecular weight (distribution) compared to the p(DMAc‐*b*‐APTAC) synthesized in the absence of the template (Figure 1b, blue *vs*. red trace, *M*_n, PIESA_ = 12.5 kg/mol, *Ð* = 1.13 vs. *M*_n, DM_ = 11.4 kg/mol, *Ð* = 1.13). These comparable molecular weights imply that a comparison between the direct mixing and PIESA‐derived micelles to elucidate the importance of the assembly pathway is justified. Finally, we would like to emphasize that the high intensity signal (present in the SEC trace of the PIESA sample retention time ≈ 18.5 min) corresponds to the DNA template (Figure 1b, dotted trace).

Despite the near‐identical composition of the direct mixing and PIESA samples, particles with distinct morphological features were formed as is evident from cryo‐TEM images. Whereas the nanoparticles prepared via the direct mixing pathway feature a spheroidal morphology with internal concentric circles (Figure 1c), the PIESA‐derived particles are characterized by a hexagonal disk‐shape that propagates from the highly ordered hexagonal packing of DNA within the particle cores (Figure 1d). For both assembly pathways, the final particle dispersions are dominated by either one of the morphologies (see Figure S8 and S9). Cryo‐TEM analysis revealed that the spheroidal particles have a diameter of 27 ± 7 nm, whereas the hexagonal disks are characterized by an edge length of 26 ± 9 nm and an average thickness of 33 ± 6 nm. This thickness was determined from particles displaying their side view (see Figure S11).

It is important to note that these morphologically distinct structures do not originate from residual monomer in the PIESA mixture. This was confirmed by performing two direct mixing experiments in which either p(DMAc_119_‐*b*‐APTAC_50_) or p(DMAc_119_‐*b*‐APTAC_43_) was used as the encapsulating polymer. To ensure an identical number of APTAC‐derived cationic charges and to mimic the endpoint of a PIESA reaction, seven APTAC monomer equivalents were added per p(DMAc_119_‐*b*‐APTAC_43_) chain. In both cases, spheroidal particles with a concentric inner ring structure were identified as the predominant particle morphology. (see Figure S8). Furthermore, the low residual monomer concentration remaining after PIESA (≈ 0.75 mM at 85% APTAC conversion) is not expected to provide sufficient plasticization to affect the particle morphology, as the critical salt concentration of these particles is substantially higher (≈ 250 mM, see Figure S7).

Dynamic light scattering (DLS) measurements showed only minor to negligible changes in the size distributions after 9 months of equilibration at room temperature for both particle morphologies, demonstrating excellent long‐term colloidal stability (see Figure S6). This stability is likely provided by a charge‐neutral pDMAc corona. However, direct observation of this corona, for example by SAXS was not possible due to the low scattering contrast of the presumed highly hydrated corona (see Section S5.2 and Table S5, S6).

The internal structure and high degree of order of both direct mixing and PIESA‐derived particles are reflected in a Fast Fourier Transform (FFT) representation of a single particle. Especially, the high intensity spots for the hexagonal disks indicated a regular lattice spacing of the encapsulated DNA fragments with a characteristic interaxial DNA spacing of 2.9 nm (see Figure S12). Considering that the dsDNA has a radius of 1.15 nm, as experimentally determined with SAXS (described with a rigid cylinder form factor model, see Section S5.5), this spacing leaves approximately 0.6 nm of interstitial space to accommodate the polycationic block, indicating tight and efficient packing. SAXS‐based estimates for the loading capacity will be discussed later. As mentioned before, DNA ordering into hexagonal lattices is likely facilitated by their short length (22 base pairs). Staying well below the persistence length of dsDNA (∼50 nm; ∼150 base pairs) [41, 42], ensures that the nucleic acids behave as rigid rods that can crystallize under the influence of confinement driven by electrostatic [43] or depletion forces [44]. Although internal hexagonal ordering of short dsDNA has been reported in wormlike coacervate micelles [9, 10] and protamine/DNA complexes [45], the PIESA‐derived particles are uniquely distinguished by the combination of their nanoscale dimensions and low structural dispersity.

### 2.2 Dissecting Structural Evolution by TR‐SAXS

To examine the two assembly pathways in greater detail, synchrotron TR‐SAXS experiments were conducted (Figure 2). The high temporal resolution of this technique enabled a comprehensive view of their structural evolution on colloidal length scales [46]. In brief, the direct mixing experiments were replicated in a stopped‐flow mixing device previously used to capture the dynamics of rapidly self‐assembling systems [24, 47, 48]. The device allowed for in situ recording of SAXS profiles nearly instantaneous after rapid mixing of the dsDNA and BCP solutions (dead time of mixing = 2 ms). Successive SAXS frames were acquired with logarithmically increasing delays to efficiently capture both early and late assembly kinetics. (see Section S2.5.1 and Figure S14b). PIESA reactions were performed under identical conditions (as in the lab) and aliquots were drawn from the reaction vessel at a constant flow rate adjusted to match the polymerization kinetics while optimizing the temporal spacing of the SAXS acquisition (see Section S2.5.2 and Figure S14a).

**FIGURE 2:**
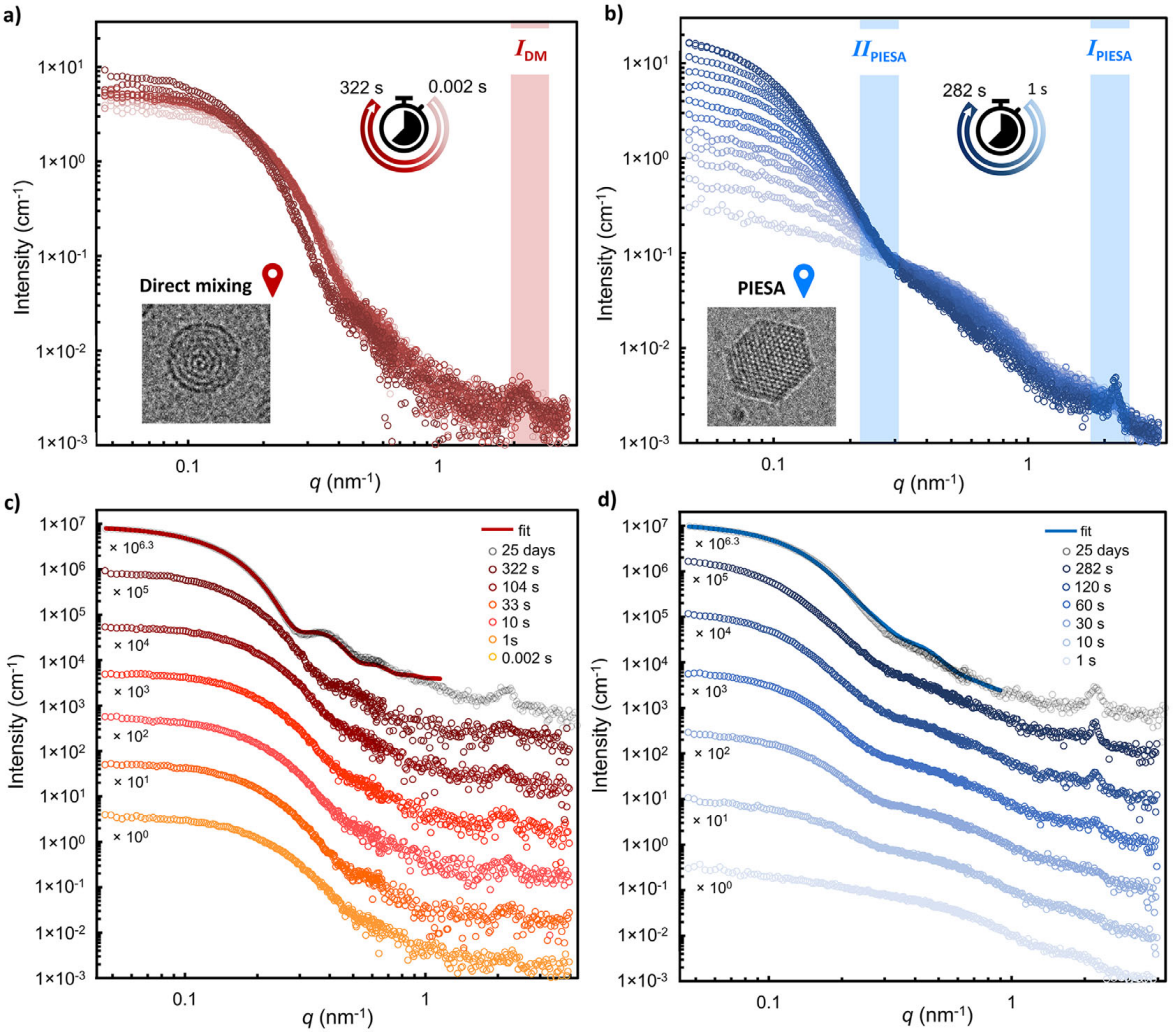
TR‐SAXS profiles on the absolute intensity scale recorded during (a) direct mixing and (b) a PIESA reaction. Whereas (internal, ***I*_DM_**) structure formation for direct mixing is nearly instantaneous, PIESA shows gradual structure formation and profiles featuring an iso‐scattering point at *q*_iso_ = 0.29 nm^−1^ (***II*_PIESA_**) and evolving sharp Bragg peak at *q*_Bragg_ = 2.2 nm^−1^ (***I*_PIESA_**). A selection of TR‐SAXS profiles scaled with arbitrary multiplication factors (10^0^ – 10^6.3^) during the (c) direct mixing and (d) PIESA pathways. Additionally, SAXS profiles measured after a 25‐day equilibration period were included for both pathways (top, black curves) to examine evidence of slow structural relaxation. These SAXS curves were fitted with an ellipsoidal (red line) or cylindrical (blue line) form factor model for direct mixing and PIESA, respectively. Motivations for the selected form factor models and fitting details can be found in Section S5.3 and S5.4.

The (intermediate) reaction/assembly products of both pathways were measured at a *q*‐range from 0.0065 – 4.8 nm^−1^ (Figure 2a, b). The selected *q*‐range provides access to length scales adequate to probe the overall particle dimensions (*R*_particle_< *π*/*q*_min_ ∼ 483 nm) and the internal DNA ordering (*R*_DNA_> *π*/*q*_max_ ∼ 0.65 nm) as observed in cryo‐TEM images (Figure 1). Directly mixing dsDNA with pre‐formed p(DMAc‐*b*‐APTAC) ([+] = [−] = 5 mM) results in near‐instantaneous particle assembly as indicated by the largely overlapping scattering profiles (Figure 2a). Alongside particle formation, a diffuse Bragg peak (Figure 2a, *I*_DM_; *q* = 2.2 nm^−1^) is formed corresponding to the concentric, yet periodic arrangement of the dsDNA. Equilibrating these samples for 25 days at room temperature (Figure 2c, top, black profile,) resulted in significant changes in the scattering profiles. These changes suggest restructuring of the particles leading to decreased particle dispersity, enhanced internal dsDNA ordering, and a non‐negligible increase in average size. The shape of the final scattering curve could accurately be captured by an ellipsoidal form‐factor model revealing a low degree of shape anisotropy (see Section S5.3 and Figure 2c, red line). The pronounced restructuring points towards the initial formation of non‐equilibrated structures, which is commonly observed for this assembly pathway and confirms that assembly processes on these short time scales are prone to kinetic trapping [24, 47].

In stark contrast, the time‐resolved PIESA profiles show a gradual, yet significant increase in scattering intensity at low *q* over time which is indicative of particle formation. Alongside the growth in mass/size, is the gradual formation of a pronounced Bragg peak at the same *q*‐position as observed for the direct mixing samples (Figure 2b, d). The Bragg peak position in the SAXS profiles is consistent with the hexagonal ordering observed by cryo‐TEM. For a hexagonal lattice, $a=\frac{4\pi }{\sqrt{3}q}$, where *a* represents the characteristic lattice parameter [49]. Using *q*_Bragg_ = 2.2 nm^−^^1^ yields *a* = 3.3 nm which matches the value found for the interaxial dsDNA spacing in the FFT analysis.

The Bragg peak evolution was analyzed by fitting the signals between 1.5 – 3.0 nm^−1^ with a Gaussian peak model summed with a power law (Figure 3a, b, and Section S5.1) [9, 10]. The full width at half maximum (FWHM) of the Bragg peak, which is inversely proportional to degree of order and size of the hexagonal crystalline cores, appears constant and is significantly lower compared to the Bragg peak observed for the direct mixing samples (Figure 3a, b, circle‐shaped markers). This suggests that the degree of dsDNA ordering is more distinct for the hexagonal disks compared to the direct mixing‐derived particles throughout the particle assembly process. More pronounced crystallinity during growth is in turn reflected by a gradual increase in intensity and area under the Bragg peak (Figure 3b, square‐shaped markers; see Section S5.1 for underlying calculations). Considering that the hexagonal crystal structure represents the most favorable packing of rigid rods [50], these results suggest that the slower assembly kinetics associated with the PIESA pathway allow for structural reconfiguration and relaxation, thereby promoting the formation of particles that more closely approach thermal equilibrium than their rapidly assembled direct‐mixing counterparts. A hypothetical self‐assembly mechanism is presented in Section 2.3.

**FIGURE 3:**
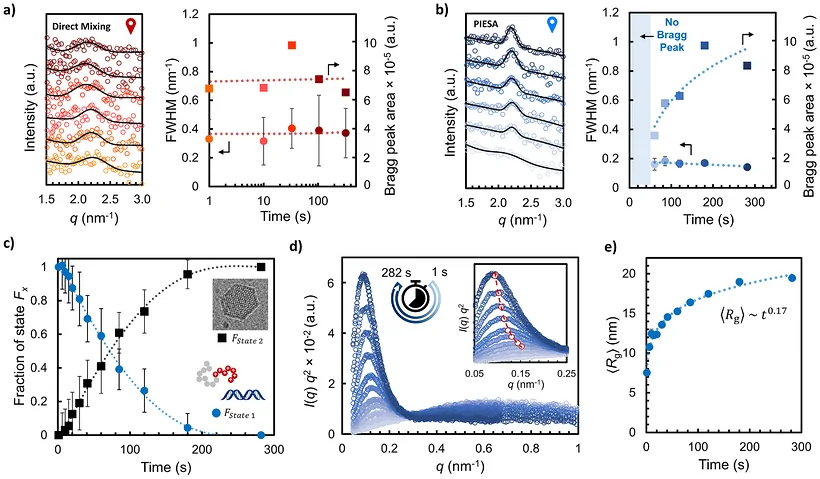
Evolution of the Bragg peak in SAXS profiles recorded during (a) direct mixing and (b) PIESA. Plotted are the full‐width half maximum (FWHM, circle‐shaped markers) and Bragg peak area (square‐shaped markers) obtained from fitting the SAXS profiles with a Gaussian peak combined with a power law relation (see Section S5.1). A logarithmic *x*‐axis (time) was chosen in panel a) for clarity. (c) Time evolution of the two states (**State 1** = dissolved macromolecular and/or uPICs (blue circle‐shaped markers); **State 2**: assembled hexagonal disks (black square‐shaped markers) during a PIESA reaction. (d) Time evolution of the Kratky plots during a PIESA reaction. Inset: Evolution in peak position in the Kratky plots. The red dotted line represents a power‐law fit to the peak maxima which are indicated by the red circles. (e) Ensemble‐averaged radius of gyration (⟨*R*_g_ ⟩) of the hexagonal disks as a function of the PIESA reaction time. Data points are fitted with a power law to indicate the average particle growth rate (blue dotted line). See Figure S17 for corresponding double‐logarithmic plot.

The highly effective packing of dsDNA, as observed with cryo‐TEM and supported by the Bragg peak positioning, was further corroborated by calculating the DNA and BCP aggregation numbers (*N*_DNA_ and *N*_BCP_, defined as the average number of encapsulated dsDNA or BCPs per particle, respectively). These aggregation numbers were determined using the average molecular weight extracted from the extrapolated forward scattered intensity at *q* → 0 on absolute scale (see Section S5.6) [51]. The *N*_DNA_ of the PIESA particles was estimated to be 234 ± 10, which is significantly higher than what is obtained for the particles with the concentric‐ring internal structuring (86 ± 12). The values for *N*_BCP_ follow the same trends and were 240 ± 11 and 76 ± 11 for PIESA and direct mixing, respectively. The calculated *N*_DNA_ for the hexagonal disks is in reasonable agreement with cargo loadings estimated by counting the number of dsDNA per layer and multiplying this value by the number of crystal layers within one disk (see Section S5.7). The number of crystal layers (2 – 4) was determined by comparing the height of the particles, either obtained directly from cryo‐TEM image analysis or from fitting the SAXS profiles with a cylindrical form factor model, with the length of a single dsDNA fragment (≈ 7.5 nm; see Section S5.5 for experimental determination of this value with SAXS). Normalizing *N*_DNA_ with respect to the particle volume, obtained by fitting the final TR‐SAXS frames to appropriate form factor models (Figure 2c, d; red and blue curves. see Sections S5.3, S5.4 for fitting details) reveals that per unit volume the hexagonal disks contain approximately 2.23 times as much dsDNA compared to the direct mixing‐derived particles. The enhanced NA packing efficiency hints towards potential benefits of using the PIESA assembly pathway for the synthesis of nano‐sized delivery vehicles, where a high‐ and efficient cargo loading is desired to optimize efficacy [12, 14, 52].

Besides the Bragg peak, the PIESA‐derived SAXS profiles also exhibit an iso‐scattering point at *q*_iso_ = 0.29 nm^−1^ (Figure 2b, *II*_PIESA_). Such feature is typically observed for systems in which a transition from one physical state into another occurs. Examples are folding and/or aggregation of proteins [53, 54], conformational changes in DNA origami nanostructures [55], and radial inwards formation of bi‐continuous emulsion gels [56]. In general, the scattering intensity of the system at a given time point during the transformation between the two states can be described by a linear combination of initial and final scattering intensity profiles multiplied by the fraction of each state: [56]

(1) $$I\;\left(q,t\right)=F_{\mathrm{State}\;1}\;\left(t\right)\;I_{\mathrm{start}}\left(q\right)+F_{\mathrm{State}\;2}\left(t\right)\;I_{\mathrm{final}}\left(q\right)$$

For the PIESA process, we define **State 1** as non‐particular, dissolved macromolecular and/or small charge‐neutral unit polyion complexes (uPICs) that serve as building blocks for particle assembly (see **Section 2.3** for justification for uPIC presence). For these species, the scattered intensity is dominated by the dsDNA due to its significantly higher scattering contrast with respect to the medium compared to the BCP (see Section S5.6). **State 2** is defined as the state in which the DNA and BCPs are encapsulated into nanoparticles with *F*_State 2_ (*t*) =  1 −  *F*_State 1_(*t*). Using this definition, **State 2** captures all particle‐based states and is compatible with particle growth as will be discussed later in relation to Figures 4 and 3e. The fractions of state were calculated by averaging the scattering intensities of the data points on either side of the iso‐scattering point within the *q* ranges of 0.046–0.15 nm^−1^ and 0.45–0.53 nm^−1^ (see Section S5.8). In agreement with the gradual build‐up of the scattered intensity, a distinct and smooth transition between the two states was found (Figure 3c). We confirmed that *F*_State 2_(*t*) could also be extracted by assuming that each recorded SAXS frame represents a linear combination of two reference scattering profiles, representing the starting and final state of the assembly process. These reference states were here defined as the final frame in the TR‐SAXS measurement (*F*_State 2_ = 1) and the frame preceding particle nucleation (*F*_State 1_ = 1). Details regarding the calculation can be found in Section S5.8. This approach provides independent evidence for the validity of the presented two‐state model. Notably, *F*_State 2_(*t* ) could also be accurately reproduced by normalizing the area under the Bragg peak at a given time point by the maximum Bragg peak area obtained in the final SAXS frame (see Section S5.8). Given that BCP/dsDNA encapsulation follows the same time evolution as the total crystallinity implies that that dsDNA ordering is persistent throughout the particle growth and dsDNA ordering is not preceded by an amorphous state.

**FIGURE 4:**
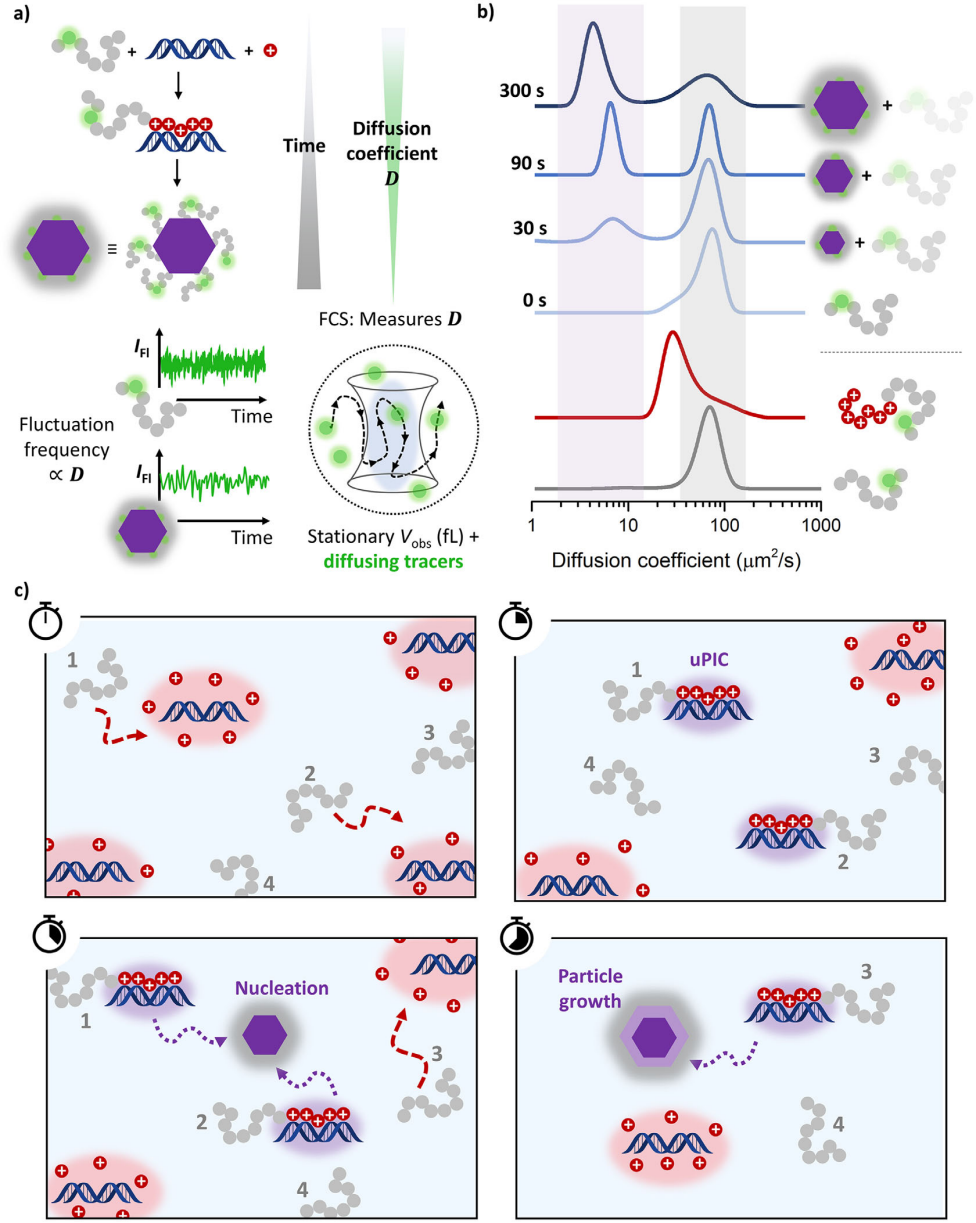
(a) Schematic depiction of a PIESA process using fluorescently‐labeled macro‐chain transfer agents (macroCTA, green). As time progresses, the macroCTA is chain‐extended, forms electrostatic complexes with the double‐stranded DNA (dsDNA, blue) template and finally gets incorporated into the hexagonally disk‐shaped particles (purple). The fate of the labeled macroCTA, and thus progression of a PIESA process on a molecular level, is detectable by measuring its diffusion coefficient (*D*) as a function of time. See Section S2.7 for analytical details. **(** **b)** Distributions of *D* measured during PIESA after 0, 30, 90 and 300 s (blue curves). The samples show the coexistence of non‐extended macroCTA polymers (*D* ≈ 80 µm^2^/s) and colloidal objects that grow over time (*D* ≈ 7 – 5 µm^2^/s). **(** **c)** Schematic representation of proposed molecular mechanism underlying the PIESA process based on FCS and TR‐SAXS data. Key step is the continuous formation of uPICs governed by the initial inhomogeneous monomer distribution and the statistical nature by which a macroCTA encounters a monomer‐rich template region. Once uPICs are formed, these building blocks rapidly nucleate into particles, explaining the coexistence of particles and non‐reacted macroCTAs without any intermediate species.

The transition between states was further corroborated by constructing and evaluating the Kratky plots of a selection of time points during the PIESA process (Figure 3d). Kratky plots (i.e., *I*(*q*)*q* ^2^ *vs. q*) are particularly sensitive to differences in macromolecular conformation [57, 58]. While extended coil and rod‐like structures show an upturn in the high *q*‐region, globular and compact structures exhibit a pronounced peak in the low *q*‐region [59]. During the PIESA process, this transition is clearly observed, implying the progressive incorporation of ‘free’ dsDNA and BCPs (**State 1**) into the particle state (**State 2**). The peak of the Kratky plots gradually narrows, increases in intensity and the *q*‐position of its maximum shifts to lower values (Figure 3d, inset plot), consistent with gradual formation of well‐defined, heavier, and denser structures as well as reduced conformational heterogeneity in the mixture. In contrast, for the direct mixing pathway, the Kratky analysis yields a pronounced peak as early as 0.002 s after mixing, which remained unchanged over time (see Section S5.9).

### 2.3 Elucidating the Molecular Mechanism Governing PIESA

Having established that the PIESA procedure provides a gradual assembly pathway to internally ordered particles, we aimed to quantify the growth mechanism by complementing the colloidal length scale information provided by TR‐SAXS with a molecular picture. Fluorescence correlation spectroscopy (FCS) was selected for this purpose. FCS is a powerful technique that can sensitively probe the diffusion coefficients (*D*) of fluorescently labeled species [60, 61, 62]. In essence, one records the fluorescence intensity (*I*_Fl_) from a stationary femtoliter‐sized observation volume (*V*_obs_) that is illuminated with laser light that can selectively excite the fluorescent species of interest (Figure 4a). The fluctuations are a direct consequence of continuous diffusion of the fluorescent species in and out of *V*_obs_, making the characteristic frequency of these fluctuations proportional to *D*. Given that only labeled species are visible, FCS measurements can be conducted in complex mixtures without interference of other non‐fluorescent components. To track a PIESA process, a rhodamine‐B‐labeled macroCTA was used (Figure 4a, green circle = fluorescent tag, see Section S1.3 for its synthesis). Coupling the dye to the charge‐neutral segment ensured minimal interference with the polymerization and assembly process. Simultaneously, this strategy allows us to track the formation of BCPs, intermediate electrostatic complexes/uPICs, and colloidal particles as these species have increasing hydrodynamic sizes and thus decreasing diffusion coefficients, respectively. To this aim, the intensity fluctuations were recorded on aliquots taken during the PIESA process (utilizing the labeled macroCTA) followed by analysis using a maximum entropy method (MEM) to account for the anticipated dispersity in diffusion coefficients in an unbiased way (see Section S2.7 for more details) [63]. The obtained diffusion coefficient distributions revealed a peculiar pattern where non‐extended macroCTAs (*D* ≈ 80 µm^2^/s, bottom gray spectra as reference) coexists with colloidal particles which rapidly form and then gradually grow over time (*D* ≈ 7 – 5 µm^2^/s; corresponding hydrodynamic radii *R*_h_ ≈ 35 – 45 nm) (Figure 4b). The signal corresponding to the macroCTA decreases in intensity at the expense of the particle‐related signal over the course of the PIESA process. The residual signal of the fast‐diffusing species at the final time point can be attributed to a fraction of inactive macroCTA molecules unable to undergo chain extension reactions [64, 65]. A fraction of these ‘dead’ chains is also evident from the diffusion coefficient distribution of a molecularly dissolved BCP by the tailing of the distribution to the high *D* side (Figure 4b, red curve). These ‘dead’ chains are not seen in SEC (Figure 1b, blue chromatogram), highlighting FCS's superior sensitivity towards detecting low concentrations of diffusing species. Throughout the PIESA process, no species with intermediate values for *D* were observed, despite the adequate sensitivity of FCS to distinguish macroCTA from dissolved BCPs (Figure 4b, gray *vs*. red curve). This result implies that formed BCPs get rapidly incorporated into the growing particles.

The persistent coexistence of unreacted macroCTAs and growing particles is atypical for controlled polymerizations [66]. Under well‐established RAFT conditions in homogeneous polymerization media, all macroCTAs experience the same average growth rate towards the BCPs. However, in the PIESA system these uniform reaction conditions are not met. The cationic monomer preferentially partitions in close vicinity of the dsDNA template to balance its negative charge [33]. The resulting nanoscopic regions with increased monomer concentration act as polymerization loci. Considering that the SEC results depicted in Figure 1b show efficient and rather controlled BCP formation, significant contributions of uncontrolled free‐radical polymerization can be excluded (see Figure S5b). This implies that the macroCTA needs to enter the monomer‐rich polymerization loci to initiate the polymerization (Figure 4c, red arrows). Once BCP formation starts in the vicinity of a dsDNA template, we anticipate the formation of (nearly) charge‐neutral complexes or uPICs which are relatively monodisperse due to the limited availability of monomer set by the local charge stoichiometry (Figure 4c, purple). Subsequently, these uPICs serve as the building blocks for particle nucleation and growth. We envision that charge neutralization through uPIC formation is a prerequisite for particle assembly, as the individual components, including the template, are highly charged and therefore experience strong electrostatic repulsion. Entry of the macroCTA into the polymerization loci is purely driven by Brownian diffusion, as this charge‐neutral polymer experiences no attraction to the charged monomer and/or dsDNA. Consistent with the observed iso‐scattering point and proposed two‐state model, the probabilistic formation of uPICs accounts for the simultaneous presence of macroCTAs and particles, while also distributing the generation of these charge‐neutral complexes over the duration of the PIESA process (Figure 4c). The absence of a distinct uPIC‐associated signal in the FCS measurements and the fact that the first detected particles already have hydrodynamic radii on the order of 35 nm suggests rapid nucleation. Since larger particles are already observed early in the PIESA process, the gradual yet significant increase in scattered intensity observed with TR‐SAXS (Figure 2d) is predominantly caused by continuous formation of new particles from the generated uPICs. We would like to mention that this mechanism requires particle formation under conditions far from global charge stoichiometry. Yet, this condition is met on a macromolecular scale due to confinement of the polymerization in close vicinity of the template. Additionally, DNA‐containing coacervates and nanoparticles have been shown to form even under off‐stoichiometric charge conditions, both experimentally and in simulations [43, 45, 67, 68]. This behavior has been attributed to the chemistry and relatively high stiffness of DNA as one of the coacervation partners and deviates therefore from conventional PIC micelle systems, where particle formation is typically confined to conditions near the point of net zero charge [69, 70]. The contrasting non‐observed scenario, where all uPICs form first to generate global charge neutrality, followed by particle formation is not supported by FCS and SAXS, as it would manifest itself as the appearance of species with intermediate diffusion coefficients and a jump in scattered intensity, respectively. Likewise, a uniform particle growth is inconsistent with the SAXS data, as it would lead to a gradual shift in the first oscillation of the profiles rather than the observed iso‐scattering point (Figure 2b, d) [71].

The postulated molecular growth mechanism is further supported by evaluating the radius of gyration (*R*_g_, Figure 3e), obtained from a Guinier analysis [72], as a function of reaction time (*t*). Fitting the experimental data reveals a power‐law scaling of *R*_g_ ∝ *t* ^0.17^ (R^2^ ≈ 0.98, Figures 3e and S17 for the corresponding double‐logarithmic plot). This scaling might be interpreted as an average particle growth rate that is significantly slower compared to conventionally surface‐reaction limited (*t* ^0.5^) or Ostwald ripening growth processes (*t* ^0.33^) [73, 74, 75, 76]. However, this interpretation is not consistent with the rapid particle nucleation and uPIC incorporation observed with FCS (Figure 4b). The slow increase in *R*_g_ can, however, be rationalized when considering the temporal heterogeneous character of the PIESA system. Because uPIC formation, and therefore particle nucleation, occurs over an extended period, particles at different stages of formation coexist at any given time. The measured *R*_g_ will reflect all these species and gives therefore an ensemble averaged result (⟨*R*_g_⟩). Throughout the PIESA process, an increasing number of particles reach their final dimensions, generating a larger contribution to the measured *R*_g_. The validity of this reasoning was confirmed by calculating ⟨*R*_g_⟩ from a simulated SAXS profile comprising various ratios of disk‐like particles with distinct dimensions (see Section S5.10 and Figure S25). Although we believe this particle coexistence is the major contributor for the slowly evolving *R*_g_, other factors might amplify this behavior. As mentioned earlier, dsDNA ordering is already present during the early stages of the PIESA process and scales with the total fraction of dsDNA in the particle state (**State 2,** Equation (1)). This implies that all incoming uPICs need to find lattice positions onto the particle surface. Drawing analogies from protein crystallization [77], we hypothesize that initially incorrect docking of the uPICs can suppress particle growth by blocking neighboring lattice sites. Only after surface reconfiguration and diffusion of incorrectly bound species, additional uPICs can be added. Additionally, we note that the densification observed in the Kratky analysis (Figure 3d) may result in a less pronounced increase in *R*_g_, as the particle mass becomes increasingly concentrated near the center of mass as the PIESA process proceeds.

## 3 Conclusion

This study provides a striking example of pathway complexity in the electrostatically‐driven assembly of dsDNA with oppositely charged BCPs into PIC micelles. Two contrasting assembly pathways were investigated, namely (***i***) rapid mixing of dsDNA with preformed BCPs and (***ii***) a polymerization‐induced electrostatic self‐assembly route in which the BCP synthesis was templated by the dsDNA. Altering the pathway had a distinct influence on particle morphology with the PIESA process yielding hexagonal disk‐shaped particles with highly ordered internal packing of the stiff dsDNA molecules. Performing in situ TR‐SAXS measurements revealed near‐instantaneous particle formation for the direct mixing approach, whereas PIESA spreads out the assembly process over a longer period. The SAXS profiles recorded during a PIESA process featured a continuously developing Bragg peak and iso‐scattering point, reflecting progressive dsDNA internal ordering and a gradual transition between a non‐particulate and particulate state, respectively. Detailed FCS measurements elucidated the molecular underpinning of the PIESA process, which strongly suggests the time‐distributed formation of small, charge‐neutral complexes (uPICs) that subsequently act as building blocks for the nanoparticles. Having a continuous supply of building blocks spreads out particle formation and facilitates building the thermodynamically favored hexagonal packing of the relatively stiff dsDNA cargo.

By combining a comprehensive set of analytical techniques with high spatial and temporal resolution, this study demonstrates that the physicochemical characteristics of self‐assembled nanoparticles can be tuned independently of their chemical composition by controlling the assembly pathway. Together with the proven long‐term colloidal stability, these findings open new opportunities to tune PIC micelle formulations in terms of morphology and cargo loading. Future studies will define the scope and limitations of this strategy for programmable particle size and shape control across a broader chemical space, while evaluating its potential for (in vitro) NA‐delivery.

## Author Contributions

**Lennart van den Hoven**: conceptualization, investigation, validation, methodology, formal analysis, writing – original draft, visualization. **Neshat Moslehi**: investigation, validation, methodology, formal analysis, writing – review and editing. **Eline F. Brink**: investigation, writing – review and editing, methodology. **Austin Hubley**: investigation, methodology, writing – review and editing. **Ilja K. Voets**: writing – review and editing, supervision. **Tina Vermonden**: writing – review and editing, supervision. **Elham Mirzahossein**: investigation, formal analysis, writing – review and editing, supervision. **Bas G. P. van Ravensteijn**: conceptualization, writing – original draft, writing – review and editing, supervision, project administration, funding acquisition, visualization.

## Conflicts of Interest

The author declares no conflicts of interest.

## Supporting information



A detailed description of the experimental and analytical procedures, polymer‐ and particle characterization (cryo‐TEM, SEC, NMR, DLS), schematic overview of used experimental set‐ups (photo‐reactor and SAXS sample environments), and additional SAXS data analysis are compiled in the Supporting Information (SI). See DOI: [https://doi.org/10.1002/anie.9345533]. The raw data underlying this study can be found in the Yoda (DOI: [https://doi.org/10.24416/UU01‐9TNAVZ]) and ESRF (DOI: [https://doi.esrf.fr/10.15151/ESRF‐DC‐2498341338]) data repositories.  The authors have cited additional references within the Supporting Information [78, 79, 80, 81, 82, 83, 84, 85, 86, 87, 88, 89].
**Supporting File**: anie74363‐sup‐0001‐SuppMat.pdf.

## Data Availability

The data that support the findings of this study are openly available in the Yoda (DOI: [https://doi.org/10.24416/UU01‐9TNAVZ]) and ESRF (DOI: [https://doi.esrf.fr/10.15151/ESRF‐DC‐2498341338]) data repositories.
