# Supplementary material for: Pathway Complexity Defines the Shape of Nucleic Acid‐Loaded Polyion Complex Micelles: Spheroids vs. Hexagonal Disks
Source: Angew Chem Int Ed Engl. 2026 Sep 10;65(44):e9345533. doi: 10.1002/anie.9345533 (PMC13703908; doi:10.1002/anie.9345533)
Supplement: Supplementary file 1 [file ANIE-65-e9345533-s001.pdf]

Supporting information

Pathway Complexity Defines the Shape of Nucleic Acid-Loaded Polyion Complex Micelles: Spheroids *vs*. Hexagonal Disks

*Lennart van den Hoven[1], Neshat Moslehi[2], Eline F. Brink[1], Austin Hubley[3], Ilja K. Voets[2], Tina Vermonden[1], Elham Mirzahossein[1], Bas G. P. van Ravensteijn[1]**

[1] Division of Pharmaceutics, Utrecht Institute for Pharmaceutical Sciences, Faculty of Science, Utrecht University, Utrecht 3584 CG, The Netherlands.

[2] Laboratory of Self-Organizing Soft Matter, Department of Chemical Engineering and Chemistry & Institute for Complex Molecular Systems, Eindhoven University of Technology, P.O. Box 513, 5600 MB Eindhoven, The Netherlands

[3] European Synchrotron Radiation Facility (ESRF), The European Synchrotron, Grenoble 38043, France

*To whom correspondence should be addressed: b.g.p.vanravensteijn@uu.nl

Table of Contents

S1 **Materials & Synthesis Methods** 3

S2 **Characterization Methods** 8

S3 **Polymer- and Particle Characterization** 14

S4 **Experimental Set-ups** 22

S5 **SAXS Data Analysis** 23

S6 **References** 40

S1. Materials & Synthesis Methods

S1.1 Materials. 2,2'-Azobis(2-methyl-propionitrile) (AIBN, ≥98%), 1,3,5-trioxane (>99%), deuterium oxide (99.9%), dimethyl sulfoxide-d_6 (99.9%), prepacked columns for inhibitor removal (hydroquinone and monomethyl ether hydroquinone), (3-acrylamidopropyl)trimethylammonium chloride solution (APTAC, 75 wt% in H_2O), and lithium phenyl-2,4,6-trimethylbenzoylphosphinate (LAP, >95%) were purchased form Merck - Sigma Aldrich. Diethyl ether (stab./BHT, AR grade) and dimethyl formamide (DMF, peptide synthesis) were purchased from Biosolve. *N*,*N*-dimethylacrylamide stabilized with MEHQ (DMAc, >99%) was purchased from Tokyo Chemical Industries. 4-Cyano-4 [(ethylsulfanylthiocarbonyl)sulfanyl] pentatonic acid (CECP, >90%) was purchased from Polymer Source. Acryloxyethyl thiocarbamoyl Rhodamine B (RhoB acrylate) was purchased from Polysciences. Double-stranded DNA (dsDNA), featuring a base pair sequence derived from the study by Lueckheide et al.,[1] was acquired from Integrated DNA Technologies (purification method: Standard desalting):

5'-TCAACATCAGTCTGATAAGCTA-3'

3'-AGTTGTAGTCAGACTATTCGAT-5'

All chemicals were used as received. Ultrapure water (resistivity = 18.2 MΩ·cm) was employed throughout the study, except for dialysis, where singly deionized water was used.

S1.2 Synthesis of a poly(*N*,*N*-dimethylacrylamide) (pDMAc)-based macro-chain transfer agent (macroCTA). CECP (50.4 mg, 0.19 mmol), inhibitor-free DMAc (2.7 g, 27.2 mmol), AIBN (2.0 mg, 0.012 mmol), and 1,3,5-trioxane (as internal standard, 51.1 mg, 0.57 mmol) were dissolved in anhydrous DMF (4.8 mL) in a 15 mL Schlenk flask. The flask was sealed with a Suba-Seal septum, and the solution was degassed by purging with N_2 for 30 min under continuous stirring. The polymerization was initiated by submerging the reaction flask into a preheated oil bath at 70 °C under constant N_2 overpressure. The polymerization reaction was quenched after 2 h by exposure to ambient atmosphere and placing the reaction flask in an ice bath. The reaction

mixture was then precipitated into ice cold diethyl ether (800 mL). The polymer was retrieved via filtration and dried overnight under vacuum, yielding 1.7 g of polymer (61% yield).

^{1}H-NMR analysis in DMSO-d_6 gave a monomer conversion of 84% by comparing the intensity of the residual acrylamide signals (δ = 5.55 – 6.75 ppm) with the trioxane internal standard (δ = 5.1 ppm). This conversion corresponds to an average degree of polymerization (*DP*) of 119. No residual monomer was detected after precipitation. See **Figure S1** for NMR spectrum and associated analysis. Size exclusion chromatography (SEC) yielded M_n = 5.9 kg/mol and *Đ* = 1.19 (**Figure S4**).

S1.3 Synthesis of a rhodamine-B labeled poly(*N*,*N*-dimethylacrylamide) (pDMAc)-based macro-chain transfer agent (macroCTA). CECP (12.4 mg, 0.047 mmol), inhibitor-free DMAc (0.56 g, 5.6 mmol), AIBN (1.5 mg, 0.0091 mmol), RhoB acrylate (5.4 mg, 0.0083 mmol), and 1,3,5-trioxane (as internal standard, 9.7 mg, 0.11 mmol) were dissolved in anhydrous DMF (1 mL) in a 15 mL Schlenk flask. The flask was sealed with a Suba-Seal septum, and the solution was degassed by purging with N_2 for 30 min under continuous stirring. The polymerization was initiated by submerging the reaction flask into a preheated oil bath at 70 °C under constant N_2 overpressure. The polymerization reaction was quenched after 16 h by exposure to ambient atmosphere and placing the reaction flask in an ice bath. 1.2 mL of anhydrous DMF was added to the polymer solution, which was added into a Spectrum™ Specra/Por™ 3 Dialysis Standard RC dialysis membrane (MWCO: 3.5 kD) and dialyzed against pure DMF for 72 h in which the dialysate fluid was refreshed 3×. The polymer was retrieved via lyophilization yielding 0.5 g of polymer (86% yield). Size exclusion chromatography (SEC) yielded M_n = 7.0 kg/mol and *Đ* = 1.30. ^{1}H-NMR analysis in DMSO-d_6 gave a monomer conversion of 99% by comparing the intensity of the residual acrylamide signals (δ = 5.55 – 6.75 ppm) with the trioxane internal standard (δ = 5.1 ppm). This conversion corresponds to an average degree of polymerization (*DP*) of 120. No residual monomer or dye was detected after dialysis.

S1.4 Synthesis of poly(*N*,*N*-dimethylacrylamide-*b*-(3-acrylamidopropyl)trimethyl-ammonium chloride) p(DMAc-*b*-APTAC$_x$) block copolymers (BCPs). p(DMAc$_{119}$) macroCTA

(254 mg, 0.021 mmol for DP_{target} = 50 or 292 mg, 0.024 mmol for DP_{target} = 43; as prepared under **Section S1.2**), APTAC (215 mg, 1 mmol), and LAP (0.6 mg, 0.002 mmol) were dissolved in H_2O (3 mL) in a Schlenk flask. The flask was sealed with a Suba-Seal septum, and the solution was degassed by purging with N_2 gas for 30 min under continuous stirring. The polymerization of APTAC was started by exposing the reaction mixture to UV light (λ_{max} = 365 nm). Homogeneous illumination was achieved by using a UV-LED photo-reactor setup previously described by Sobotta et al.[2] (**Figure S13**). Briefly, UV light was supplied via a UV-LED strip (Luxalight; 240 LEDs/m, total length: 1 m), powered using a 24 V power supply that was dimmed to 12 V. The UV-LED strip was held in place by a cylindrical aluminum reactor chamber, with the average distance between the reaction vessel and the LED being 3 cm. To reduce radiative heating of the reaction mixture, the set-up was cooled with a continuous stream air supplied to the reactor chamber with an electronic fan. Polymerizations were allowed to proceed under continuous UV-light exposure for 3 h under constant N_2 overpressure and magnetic stirring. Afterwards, the polymerization reaction was quenched by exposure to ambient atmosphere. The crude reaction mixture was dialyzed against deionized water (900 mL) in a Slide-A-Lyzer™ Dialysis Cassettes, 2kDa MWCO (3 mL) for 24 h in which the dialysate fluid was refreshed 3×. The purified polymer solution was collected and freeze-dried overnight yielding 390 mg of polymer (yield = 83%) and 407 mg (yield = 80%) for DP_{target} = 50 and DP_{target} = 43, respectively.

^{1}H-NMR analysis in D_2O revealed a near quantitative monomer conversions for both DP_{target} = 50 and 43 reactions by comparing the integration values of polymer related signals (δ = 1.25 – 3.5 ppm) with the acrylic proton signal of APTAC (δ = 5.7 ppm) at t = 0 and t = 3 h (**Figure S2** and **S3**). Additionally, no residual monomer was detected after purification. SEC analysis yielded M_n = 8.2 kg/mol and $Đ$ = 1.21 for p(DMAc$_{119}$-*b*-APTAC$_{43}$ and M_n = 7.8 kg/mol, $Đ$ = 1.22 for p(DMAc$_{119}$-*b*-APTAC$_{50}$). In addition to the increase in molecular weight, successful chain extension was evident from a complete shift of the chromatograms to shorter retention times as compared to compared to the traces obtained for the employed macroCTA (**Figure S4**).

S1.5 Particle fabrication *via* Polymerization-Induced Electrostatic Self-Assembly (PIESA). p($DMAc_{119}$) macroCTA (3.6 mg, 0.3 μmol as prepared under **Section S1.2**) or its fluorescent analogue (3.7 mg, 0.3 μmol as prepared under **Section S1.3**), APTAC (3.1 mg, 15 μmol), LAP (0.18 mg, 0.6 μmol), and dsDNA (4.6 mg, 0.34 μmol) were dissolved in D_2O (3 mL) and added to a Schlenk flask. The Schlenk tube was sealed with a Suba-Seal septum, and the solution was degassed by N_2 gas bubbling for 30 min under continuous stirring. The PIESA process was initiated by exposing the reaction mixture to UV light (λ_{max} = 365 nm) using the same photoreactor set-up as described in **Section S1.4**. A 6 V operating voltage was used, and the reaction mixture was kept under a constant N_2 overpressure while being magnetically stirred. Aliquots were taken either by drawing sample from the reaction mixture with degassed syringes, or via a constant withdrawal using an automated syringe pump to enable time-resolved small-angle X-ray scattering (TR-SAXS) measurements (see **Section S2.5.2** for details). The reaction was quenched by exposure to ambient atmosphere after 5 min.

The apparent hydrodynamic radius (R_h) of the particles was determined using dynamic light scattering (DLS), while their morphology and internal dsDNA ordering was probed with cryogenic transmission electron microscopy (cryo-TEM, **Figure 1c** in **Main Text** and **Figure S8 – 12, S22**). DLS measurements were repeated 21 days and 9 months after preparation to confirm the particle's colloidal stability (**Figure S6**). The APTAC conversion was determined using ^{1}H-NMR after addition of NaCl to disintegrate the particles and ensure reliable integration values. The aromatic signals originating from the dsDNA (δ = 7.0 – 8.3 ppm) or total (residual) monomer + macroCTA signals (δ = 2.75 – 3.5 ppm) were used as reference compared to the vinylic proton signal intensities at δ = 5.7 ppm or 6.5 ppm of APTAC. This procedure was followed because the integration value of the trioxane signal proved to be unreliable in PIESA systems. An APTAC conversion of 85% was found. See **Figure S5** for NMR spectrum and associated peak integration values. SEC analysis gave M_n = 12.4 kg/mol and $Đ$ = 1.13 (**Figure 1b**, blue trace in **Main Text**).

S1.6 Particle fabrication *via* direct mixing. Equal volumes of a p($DMAc_{119}$-*b*-$APTAC_{50}$) BCP solution (0.2 mM in D_2O) and a dsDNA solution (0.226 mM in D_2O) were mixed in an Eppendorf

tube yielding a stoichiometric charge ratio of 1 ([-] = [+] = 5 mM). The complete mixture was vortexed for 10 s to facilitate the assembly process.

To account for the slight inconsistency in cationic block length and non-quantitative APTAC conversion during the PIESA reaction (see **Section S1.4** and **Figure S5**), a comparative direct mixing experiment was performed with p($DMAc_{119}$-*b*-$APTAC_{43}$) and 15% free monomer. To achieve this, a solution containing p($DMAc_{119}$-*b*-$APTAC_{43}$) and APTAC (0.2 and 1.4 mM in D_2O, respectively) was mixed with an equal volume of a dsDNA solution (0.226 mM in D_2O).

For both direct mixing experiments, cryo-TEM confirmed the formation of near-spherical particles with an internal concentric ring structure (**Figure 1** in **Main Text** and **Figure S8**). This comparison confirms that direct mixing-derived morphologies are robust and that the distinct hexagonal morphology observed for the PIESA process (**Figure 1** in **Main Text**) is an immediate consequence of the fundamentally different assembly protocol. R_h values were measured with DLS on freshly prepared particle dispersions and after a 21-day and 9-month period to confirm colloidal stability (**Figure S6**).

S2. Characterization Methods

S2.1 Nuclear Magnetic Resonance Spectroscopy (NMR). ^{1}H-NMR spectroscopy was performed in 5 mm diameter tubes in DMSO-d_6 or D_2O at 25 °C. Spectra were recorded on an Agilent 400-MR NMR spectrometer (Agilent Technologies) operated at 400 MHz. Residual solvent peaks of DMSO-*d6* (δ = 2.50 ppm) or D_2O (δ = 4.79 ppm) were used to reference the chemical shifts. In samples containing significant amounts of H_2O, a water-suppression 1D proton NMR protocol was implemented. Data was processed using MestReNova software.

S2.2 Dynamic Light Scattering (DLS). DLS experiments were performed on a Malvern Zetasizer Nano S equipped with a 633 nm He-Ne laser to determine the apparent, intensity-weighted hydrodynamic radius R_h of the particles and their polydispersity index (PDI). Scattered light was detected at 173° on dilute particle solutions at T = 25 °C. Each light scattering experiment comprised 3 – 5 measurements which in turn contained 12 individual 10 s data acquisitions. The reported values are averaged over all runs. Data were recorded and processed with Zetasizer Software 8.02 using a cumulant fitting algorithm.

S2.3 Size Exclusion Chromatography (SEC). Molecular weights M_n (number-average) and M_w (weight-average), as well as the dispersity $Đ = M_w/M_n$ were measured using a Waters e2965 AllianceXC chromatography set up, equipped with a Waters 2489 differential refractive index (dRI) detector (Waters). Separation occurred using a Aquagel-OH mixed M column (molecular weight range = 100 – 600.000 g/mol, Agilent Technologies) at 30 °C. An aqueous 0.25 M ammonium acetate buffer (pH = 5.5) + 10 %v/v DMF (0.45 μm PTFE filtered) was employed as eluent at a flowrate of 1 mL/min, with an overall runtime of 30 min. Samples were prepared at a typical concentration of 1 – 3 mg/mL and filtered with a 0.2 μm PTFE filter prior to injection. A series of linear polyethylene glycol (PEG) standards of defined and narrow molecular weights (Polymer Standards Service GmbH (PSS)) were used for calibration. Data was recorded and processed with Waters Empower 3 software.

S2.4 Cryogenic transmission electron microscopy (cryo-TEM). Vitrified thin films of the particle dispersions were prepared using an automated vitrification robot (FEI Vitrobot Mark IV) by plunge vitrification in liquid ethane. Before vitrification, 200-mesh copper grids covered with a Quantifoil R 2/2 holey carbon film (Quantifoil Micro Tools GmbH, Großlöbichau, Germany) were surface plasma treated for 2× 40 s using a Cressington 208 carbon coater (5 mA). Cryo-TEM imaging was carried out on a Glacios (Thermo Fisher Scientific), equipped with a field emission gun (X-FEG), Ceta 16M camera and a Falcon 4i direct electron detector. The microscope was operated at 200 kV acceleration voltage in bright-field TEM at a nominal magnification in the range of 8.500× to 96.000× magnification, at the defocus values ranging from −1 to −5 μm, and with 1 s acquisition time.

S2.5 Time-resolved small-angle X-Ray scattering. Time-resolved small-angle X-ray scattering (TR-SAXS) measurements were performed at the ID02 beamline at the European Synchrotron Radiation Facility (ESRF) in Grenoble, France. The incident X-ray energy was 12.23 keV with sample-to-detector distances of 10 m and 1.5 m resulting in an overall q-range of 0.0065 to 4.8 nm^{-1}, with the scattering vector $q = \frac{4\pi}{\lambda \sin(\theta/2)}$, where λ is the wavelength of the beam and θ the scattering angle. The incident beam intensity was on the order of 10^{13} photons/s. Data was recorded on an Eiger2 4M detector (Dectris). Two-dimensional scattering patterns were normalized to an absolute intensity scale, and detector corrections were applied to account for homogeneity and linearity before being azimuthally averaged to obtain one-dimensional scattering profiles, which were background-subtracted from the corresponding solvent references. In the case of background measurements, statistics were improved by averaging multiple frames. To ensure accurate background subtraction, all measurements were performed at the same point of the quartz capillary cell. Reduced SAXS profiles were logarithmically rebinned to optimize signal-to-noise while preserving the relevant structural information.

S2.5.1 Direct mixing for TR-SAXS - *In-Situ* stopped-flow mixing. Direct mixing was performed with a SF-4000 stopped-flow mixing system (Biologic, see **Figure S14b** for schematic

representation). A p($DMAc_{119}$-*b*-$APTAC_{50}$) BCP solution (0.2 mM in D_2O) and a dsDNA solution (0.226 mM in D_2O) were (100 μL each) were injected at a rate of 4 mL/s. The data acquisition was triggered at the beginning of the injection, with the first SAXS frame occurring at the end of the rapid-mixing phase. The solenoid hard stop was activated at the end of this mixing, such that the kinetic time is equal to the dead time of mixing (approximately 2.3 ms) of the stopped-flow device. Subsequent frames were recorded with increasing delay between frames following a geometric progression, enabling efficient sampling of both early and late assembly kinetics of a total duration of approximately 5 minutes. The delay between consecutive frames (Δt_n) was calculated using a geometric progression, such that $\Delta t_n = t_i + t_0 \cdot 1.12^n$, where $t_i = 45$ ms is the dead time preceding the mixing, $t_0 = 5$ ms is the initial delay between the first frames, and n is the frame number for a total of 80 frames. The exposure time was fixed at 5 ms. Injection sequences were repeated 3× yielding reproducible scattering evolution, and all measurements were performed at the same point of the quartz capillary cell (1.3 mm diameter, 0.01 mm wall thickness).

S2.5.2 PIESA for TR-SAXS - *In-situ* flow-through. *In-situ* flow-through reactions were performed as outlined in **Section S1.5.** To adapt the reaction for *in-situ* SAXS measurements, silicone tubing connected the reaction vessel to a flow-through cell (quartz capillary, 2 mm diameter with 0.01 mm wall thickness) mounted in front of the X-ray beam (**Figure S14a**). This capillary was then connected to a syringe pump (NE-1000 New Era SyringeOne) set to withdraw the PIESA mixture from the reaction vessel at a predefined rate (0.5 mL/min). The UV irradiation was triggered remotely, and SAXS measurements were acquired throughout the duration of the reaction. Beam-induced radiation damage was avoided by assuring continuous sample flow through the capillary, and a predefined dead time was included between exposures. Exposures of 50 ms were used with either a zirconium or molybdenum attenuator in place for the 1.5 and 10 m detector distances, respectively. No systematic radiation-induced changes in the scattering profiles were observed under the employed conditions.

S2.6 Static SAXS. SAXS measurements on pure dsDNA solutions were performed at the BM29 beamline (BioSAXS) of the European Synchrotron Radiation Facility (ESRF, Grenoble, France), equipped with a Pilatus3 X 2M 2D detector. The sample-to-detector distance was set to 2.87 m and the beam wavelength to 1.0 Å. Samples (dsDNA concentration: 0.113 mM in D_2O) were measured in 10 exposures of 1 s each at 20 °C, in constant flow via a flow-through quartz capillary set-up with a 1 mm diameter using the sample changer mode to minimize radiation damage. The corresponding backgrounds were measured before and after each sample. The intensity $I(q)$ of the integrated background-subtracted data was plotted as a function of the magnitude of the scattering vector q, over the range of 0.058 – 5.58 nm^{-1} using monochromatic X-rays with an energy of 12.5 keV. ATSAS primus was used for frame averaging and background subtraction.

S2.7 Maximum entropy method - Fluorescence correlation spectroscopy (MEM-FCS). Aqueous, fluorophore containing solutions (100 µL) were loaded into a chambered glass coverslip (#1.5; thickness = 170 ± 5 µm) with 18 individual wells (µ-slide, Ibidi). FCS measurements were performed 20 – 30 µm above the glass surface to prevent surface effects. For each solution, one measurement with a total duration of 3 min was performed at room temperature (21 °C). A commercial confocal microscope (Stellaris 8 FALCON FCS, Leica) using a 63×1.2 water immersion objective equipped with motorized correction collar to precisely correct for variations in cover glass thickness (HC PL APO motCORR CS2, Leica) was used to collect the FCS data. Excitation was achieved by a pulsed white light laser (WLL, λ = 440 – 790 nm, $\Delta\lambda$ = 1 nm, 80 MHz) set at the peak excitation maximum of the fluorophore under investigation (543 nm for Rhodamine-B labeled species). The emitted fluorescence light was collected through the same objective after passing through a confocal pinhole (1 airy unit at 580 nm) and directed to GaAsp-based photon-counting detectors (HyD X) with spectral detection ranges of 552 – 834 nm, respectively. The time-dependent fluctuations of the detected fluorescent signal ($F(t)$) were recorded and evaluated by an auto-correlation function ($G(\tau)$) given by the following generalized definition:

$$G(\tau) = \frac{\langle F(t)\, F(t + \tau)\rangle}{\langle F(t)\rangle^2} \qquad \textbf{Eq. S1}$$

where τ is the delay time. To analyze the heterogeneous samples with different molecular or particle mobilities, the experimental FCS auto-correlation curves were fitted using a maximum entropy method (MEM) previously reported by Sengupta et al.[3] Instead of assuming a fixed number of diffusing components, as is commonly done in multi-component FCS fitting, the MEM-FCS approach describes the auto-correlation curve as a linear combination of diffusion components distributed over a defined range of diffusion times.

$$G(\tau) = \sum_{i=1}^{n} \alpha_i \left(\frac{1}{1 + \frac{\tau}{\tau_{D_i}}}\right)\left(1 + \frac{\tau}{\kappa^2 \tau_{D_i}}\right)^{-1/2} \qquad \textbf{Eq. S2}$$

Eq. S2 operates under the assumption that dark triplet states can be neglected. In this equation, τ_{Di} is the diffusion time of the ith species and κ corresponds to the structural parameter of the focal volume (V_{obs}), which is defined as z_0/ω_0 (≈ 5 – 7 for the used set-up as determined before each measurement session by calibration using a fluorescent probe with a known diffusion coefficient; Fluorescein ≈ 400 $\mu m^2 \cdot s^{-1}$ at 21 °C) with z_0 and ω_0 representing the axial and lateral radii of V_{obs}. α_i represents the relative amplitude of the diffusive component i. The employed range of diffusion coefficients was chosen on a logarithmic scale, allowing both slow and fast diffusing populations to be represented. The calculated auto-correlation function was then constructed as a weighted sum of theoretical 3D-diffusion correlation functions, where each weight relates to a relative contribution of a given τ_{Di} to the measured signal. Please note that α_i is not simply equivalent to relative concentrations of the species due to potential variations in molecular brightness.

The distribution of τ_{Di}'s was determined by optimizing these weights. The optimization was performed by minimizing an objective function that contains two terms: a goodness-of-fit term and an entropy (S) regularization term. The goodness-of-fit term measures the difference between the measured auto-correlation curve and the reconstructed curve, while the S term

favors smooth and minimally biased distributions. In practice, the optimized function can be written as:

$$Q = \chi^2 - \lambda S \qquad \textbf{Eq. S3}$$

where χ^2 describes the weighted residual error between the experimental and fitted autocorrelation curves, S is the entropy of the diffusion time distribution, and λ is the regularization parameter that controls the balance between fitting accuracy and smoothness of the recovered distribution. By including the S term, the routine avoids overfitting the noise in the autocorrelation data and reduces the need to predefine the number of diffusing species. After optimization, the recovered values for α_i were normalized and plotted as a function of τ_{Di}'s. For each τ_{Di} the corresponding diffusion coefficient was calculated using

$$D_i = \frac{\omega_0^2}{4\tau_{D_i}} \qquad \textbf{Eq. S4}$$

3. Polymer- and Particle Characterization

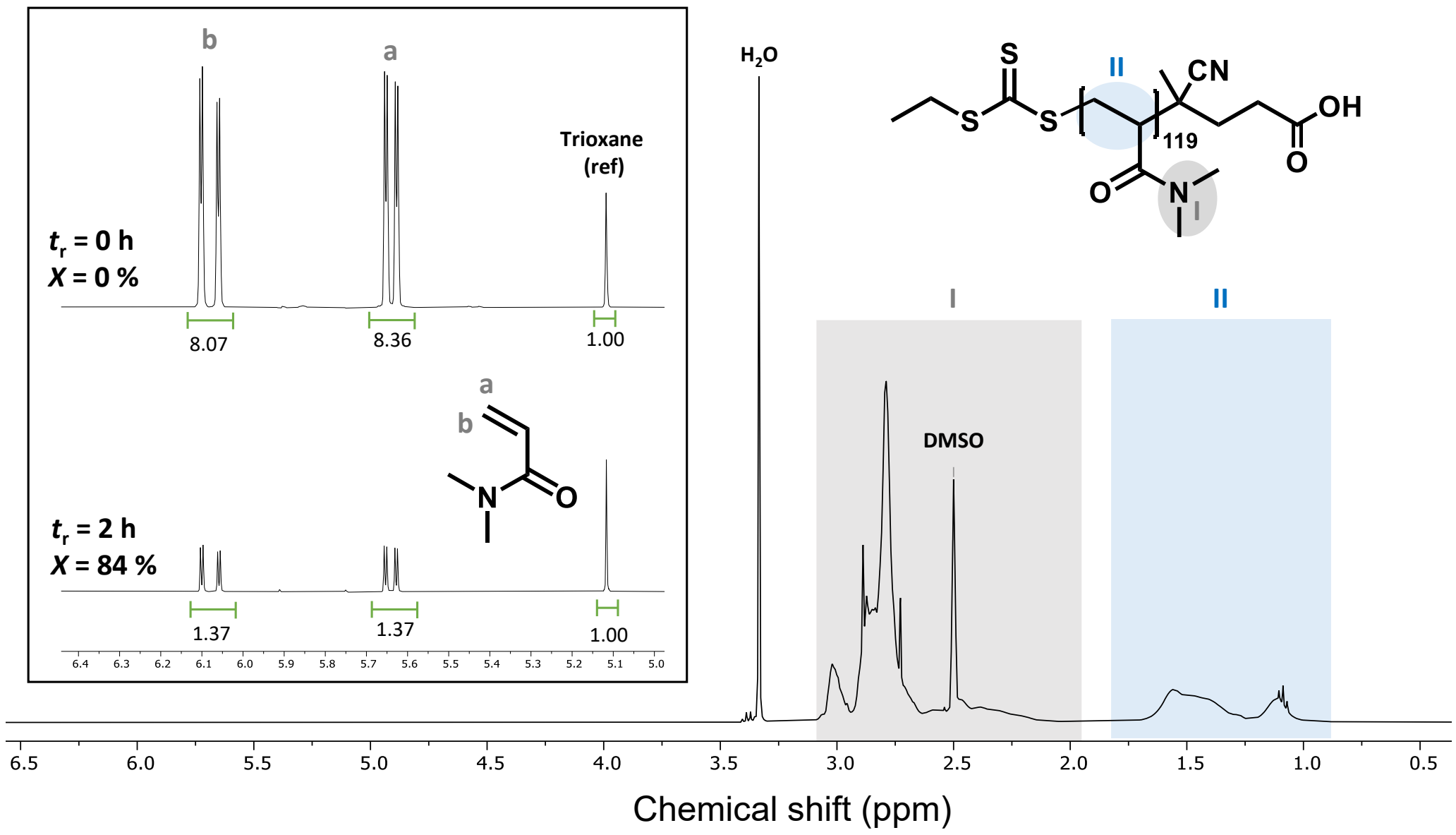

Figure S1. ¹H-NMR spectrum the poly(N,N-dimethylacrylamide) (pDMAc)-based macro-chain transfer agent (macroCTA) measured in DMSO-d_6. The characteristic methyl amide and backbone signals are highlighted by the gray and blue boxes, respectively. A DMAc conversion of 84% was calculated by comparing the intensity of the residual acrylamide signals (δ = 5.55 – 6.75 ppm) with the trioxane internal standard (δ = 5.1 ppm) at t_r = 0 and after quenching the reaction at t_r = 2 h (see inset). This conversion corresponds to an average degree of polymerization (DP) of 119.

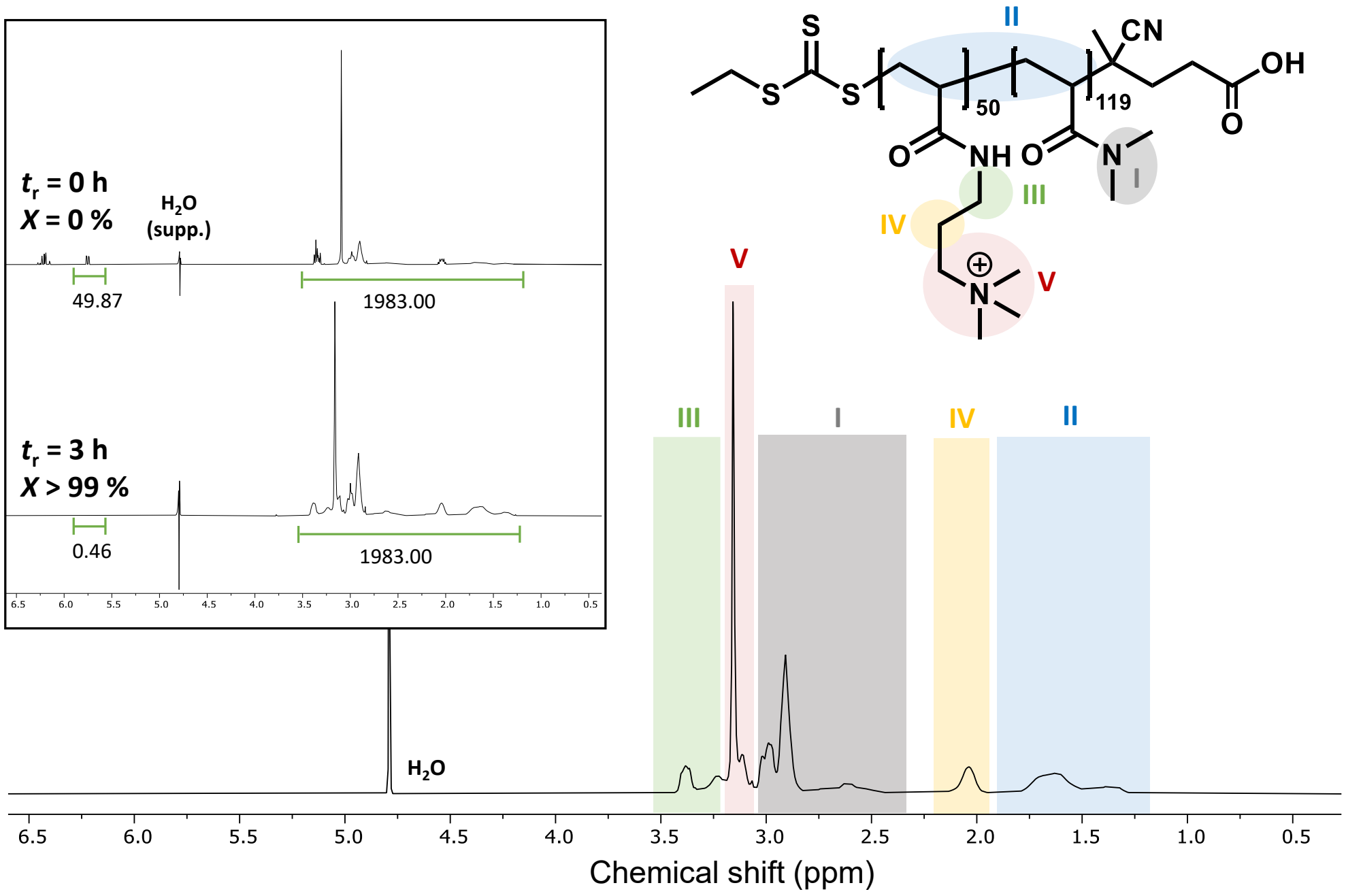

Figure S2. ¹H-NMR spectrum poly(N,N-dimethylacrylamide-b-(3-acrylamidopropyl)trimethylammonium chloride) p(DMAc-b-APTAC$_{50}$) synthesized in the absence of a template (**Section S1.4**) and measured in D_2O. The characteristic p(DMAc)-derived methyl amide and backbone signals are highlighted by the gray and blue boxes, respectively. APTAC reacted signals are indicated by the green, red, and yellow shaded areas. The APTAC conversion was determined to be (near) quantitative by comparing the integration values of polymer related signals (δ = 1.25 – 3.5 ppm) with the acrylic proton signal of APTAC (δ = 5.7 ppm) at t_r = 0 and t_r = 3 h (see inset). This conversion corresponds to an average degree of polymerization (DP) of 50 for the APTAC block.

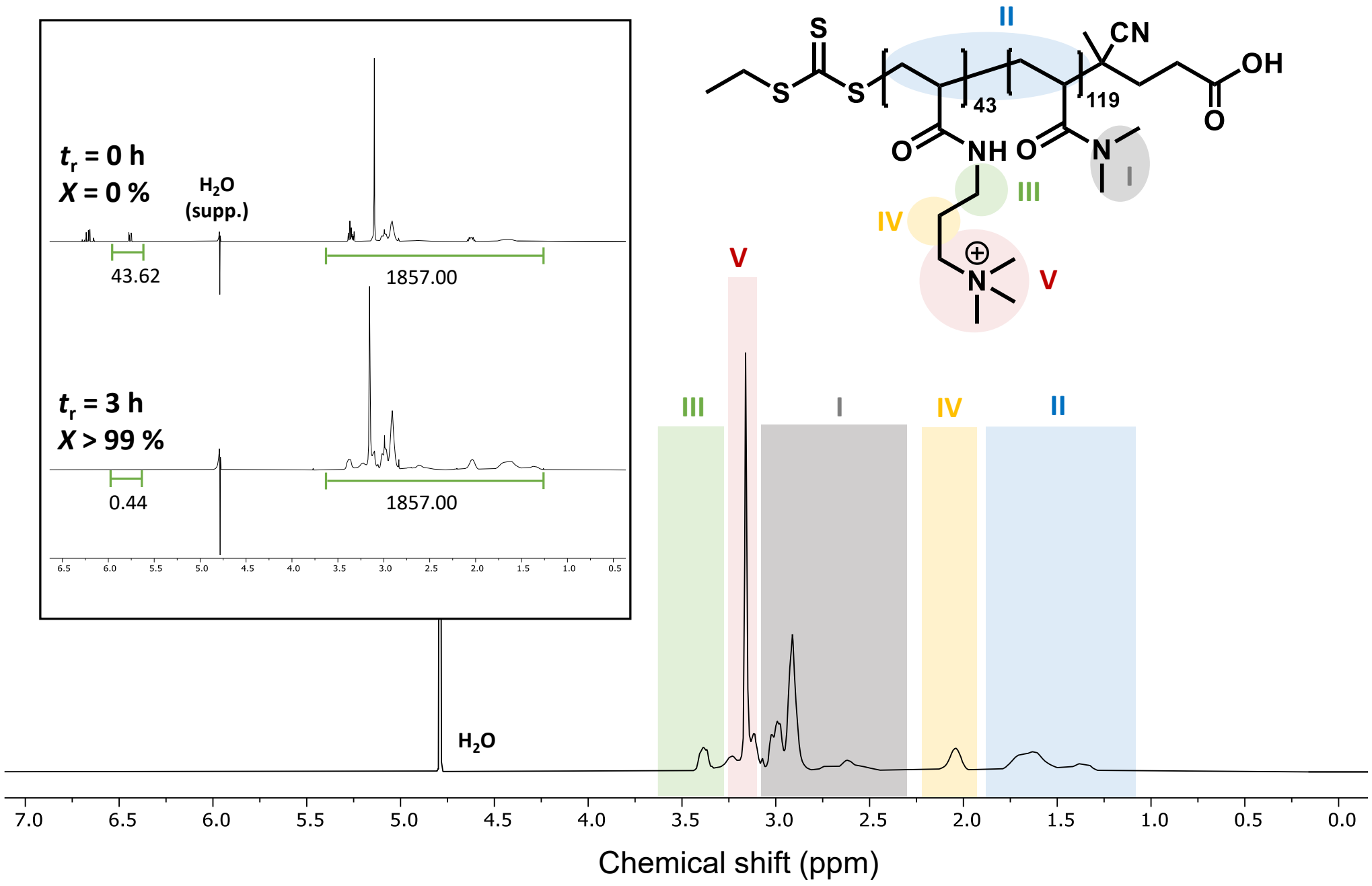

Figure S3. ^{1}H-NMR spectrum poly(*N*,*N*-dimethylacrylamide-*b*-(3-acrylamidopropyl)trimethylammonium chloride) p(DMAc-*b*-APTAC$_{43}$) synthesized in the absence of a template (**Section S1.4**) and measured in D_2O. The characteristic p(DMAc)-derived methyl amide and backbone signals are highlighted by the gray and blue boxes, respectively. APTAC reacted signals are indicated by the green, red, and yellow shaded areas. The APTAC conversion was determined to be (near) quantitative by comparing the integration values of polymer related signals (δ = 1.25 – 3.5 ppm) with the acrylic proton signal of APTAC (δ = 5.7 ppm) at t_r = 0 and t_r = 3 h (see inset). This conversion corresponds to an average degree of polymerization (*DP*) of 43 for the APTAC block.

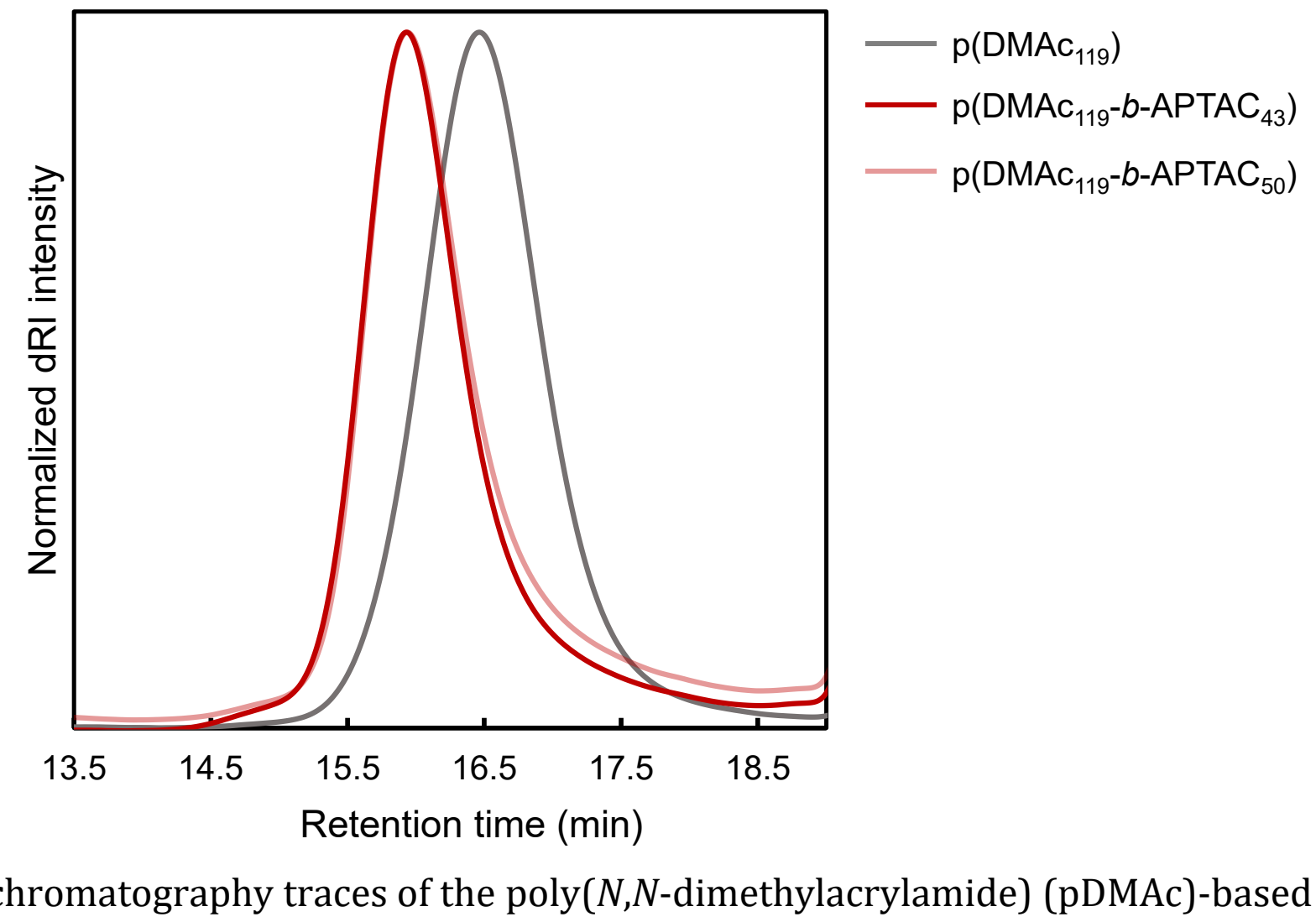

Figure S4. Size exclusion chromatography traces of the poly(*N*,*N*-dimethylacrylamide) (pDMAc)-based macro-chain transfer agent (macroCTA, pDMAC$_{119}$, gray) and poly(*N*,*N*-dimethylacrylamide-*b*-(3-acrylamidopropyl)trimethyl-ammonium chloride) with APTAC blocks having a *DP* of 43 (p(DMAc-*b*-APTAC$_{43}$), dark red) and a *DP* of 50 (p(DMAc-*b*-APTAC$_{43}$), light red). The block copolymers were synthesized in the absence of a template (**Section S1.4**). Derived number average molecular weights (M_n) and dispersities ($Đ$): p(DMAc$_{119}$): M_n = 5.9 kg/mol, $Đ$ = 1.19; p(DMAc$_{119}$-*b*-APTAC$_{43}$): M_n = 8.2 kg/mol, $Đ$ = 1.21; p(DMAc$_{119}$-*b*-APTAC$_{50}$): M_n = 7.8 kg/mol, $Đ$ = 1.22. *Please note that these M_n's were determined in a different SEC run than those reported in the **Main Text**. Consequently, minor differences in the reported values reflect the inherent run-to-run calibration variability, despite nearly identical elution times.*

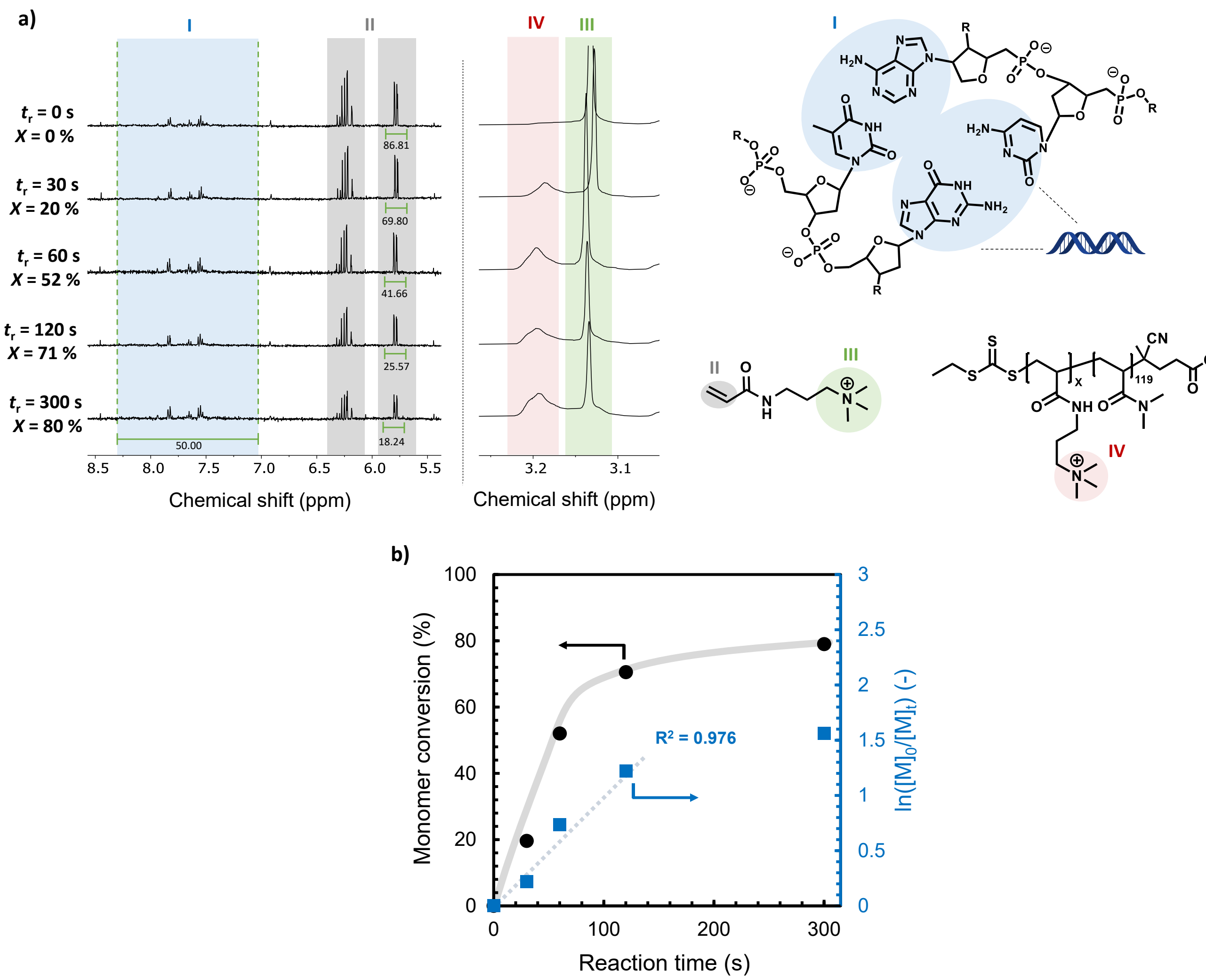

Figure S5. a) ^{1}H-NMR spectra of aliquots collected during a polymerization-induced electrostatic self-assembly (PIESA) reaction (**Section S1.5**) at t_r = 0, 30, 60, 120 and 300 s measured in D_2O. The characteristic signals of the quaternary ammonium moieties of the (3-acrylamidopropyl)trimethylammonium chloride (APTAC) monomer and corresponding polymer block are highlighted in green and red, respectively. The low-intensity aromatic signals (blue) originate from the double-stranded DNA used as template in the PIESA process. The APTAC conversion was determined for each time point by comparing the integration values of the aromatic protons (δ = 7.0 – 8.3 ppm, blue) with the acrylic proton signal of APTAC (δ = 5.7 – 5.9 ppm, gray). **b)** Plot of the APTAC conversion as function of reaction time (black circles) and the corresponding semi-logarithmic kinetic representation of the data (blue squares). The semi-logarithmic data follows a linear trend, suggesting the polymerization follows first-order kinetics and is thus controlled. Typically, conversions of 80 – 85% were obtained for all PIESA reactions. A conversion of 85% translates to an average degree of polymerization (*DP*) of 43 for the APTAC block.

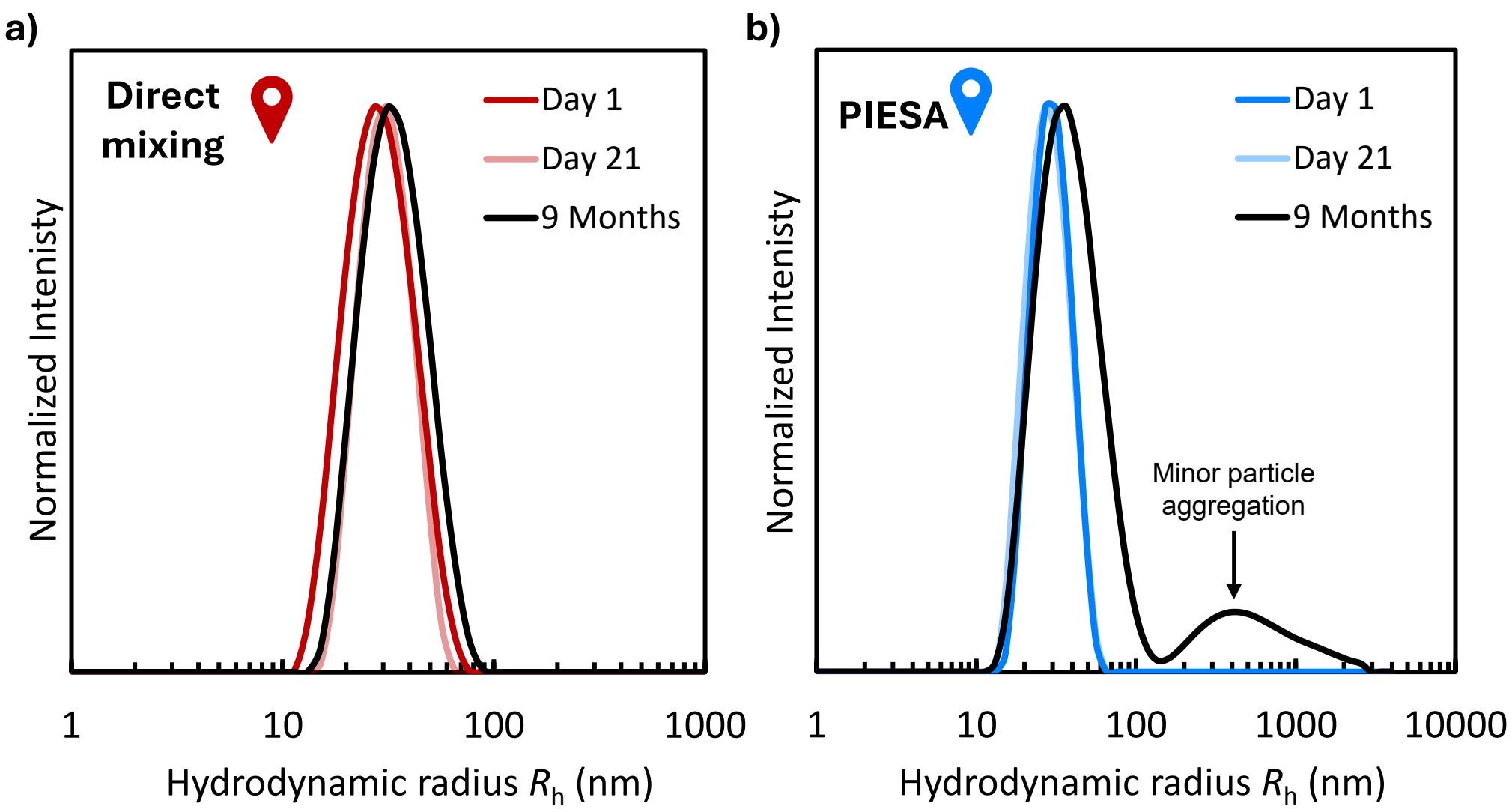

Figure S6. Intensity-weighted size distributions as measured with dynamic light scattering (DLS) for particles prepared via **a)** direct mixing and **b)** polymerization-induced electrostatic self-assembly (PIESA). Samples were measured directly after synthesis (day 1) and after storing the particles for 21 days and 9 months at room temperature. Extracted R_h values and polydispersity indices (PDI): Direct Mixing - Day 1: R_h = 24 nm, PDI = 0.13; Day 21: R_h = 27 nm, PDI = 0.055; 9 Months: R_h = 28 nm, PDI = 0.121, PIESA - Day 1: R_h = 25 nm, PDI = 0.060; Day 21: R_h = 27 nm, PDI = 0.079; 9 Months: R_h = 35 nm, PDI = 0.30.

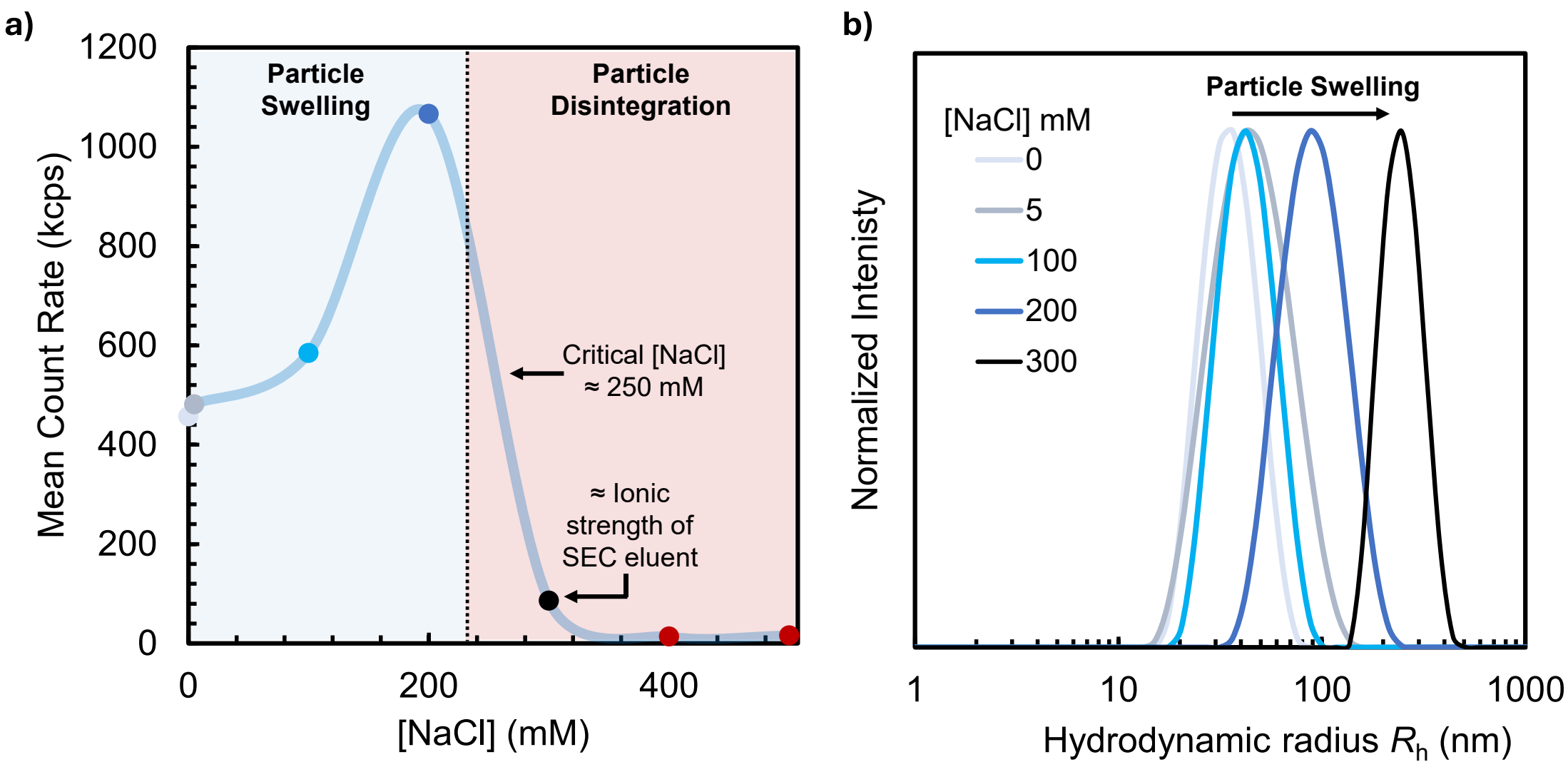

Figure S7. a) Scattered intensity of polymerization-induced electrostatic self-assembly (PIESA)-derived particles measured with dynamic light scattering (DLS) as a function of salt concentration. Salt weakens the electrostatic interactions between the encapsulating polymers and dsDNA, which is initially manifested as particles swelling (blue shaded area) followed by particle disintegration (red shaded area). The critical salt concentration and ionic strength of the SEC eluent used throughout the study are highlighted. Samples were measured at 25 °C and equilibrated at the given salt concentrations overnight. DLS measurements were performed at a fixed attenuator (setting = 8). **b)** Normalized intensity-weighted size distributions as a function of salt concentration for the data shown in panel a). Shifting of the size distributions to larger hydrodynamic sizes (R_h) confirms particles swelling. Please note that size distributions could not be constructed for samples containing [NaCl] > 300 mM due to the absence of any scattering above background levels. The size distribution for [NaCl] = 300 mM (black curve) represents a small fraction of particles that did not disintegrate.

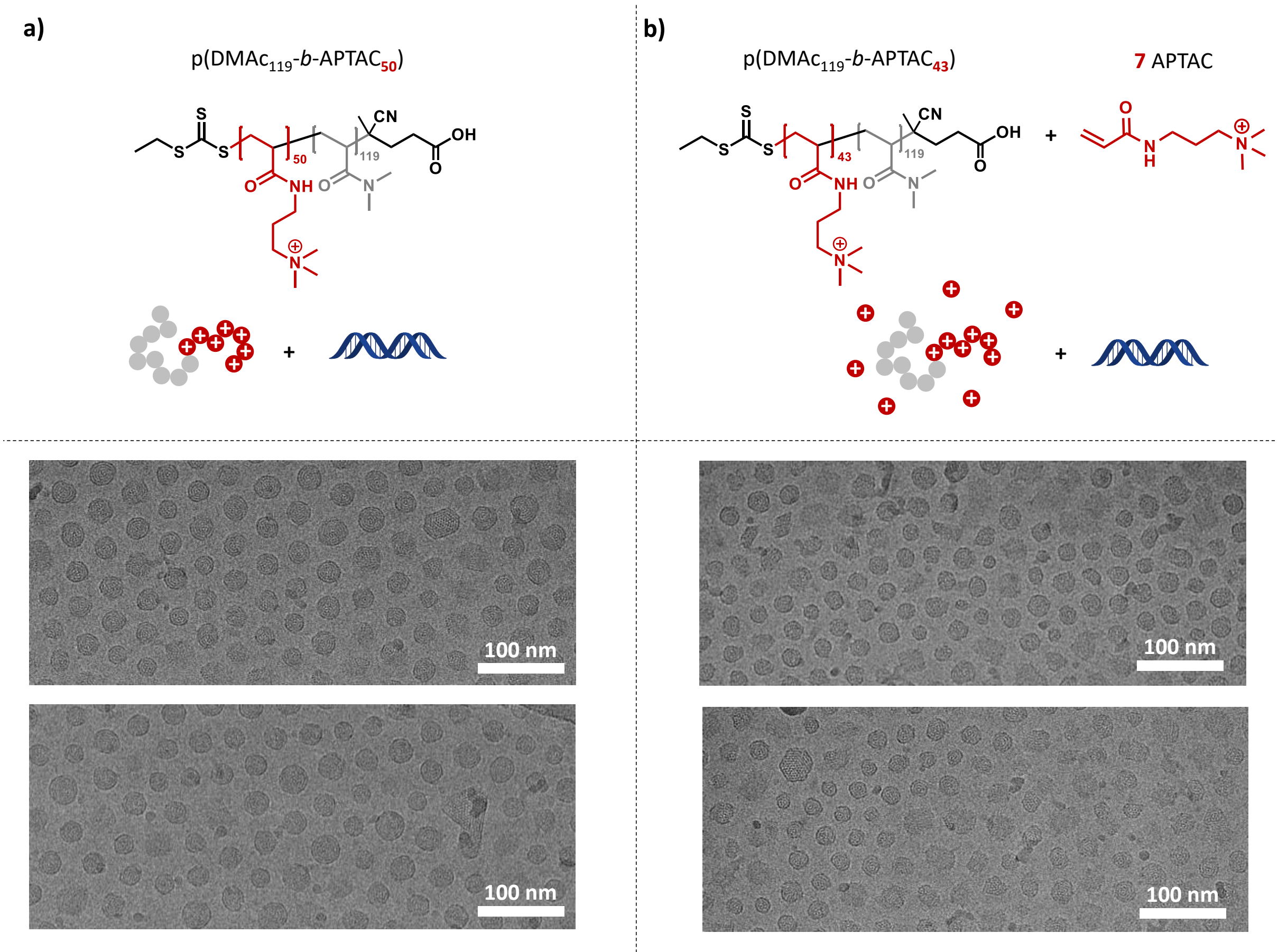

Figure S8. Cryogenic transmission electron microscopy (cryo-TEM) images of dsDNA-loaded particles prepared via the direct mixing assembly approach using either **a)** p(DMAc$_{119}$-*b*-APTAC$_{50}$) or **b)** p(DMAc$_{119}$-*b*-APTAC$_{43}$) as encapsulating polymer. To ensure the same number of APTAC related cationic charges and mimic the end point of a PIESA reaction, 7 APTAC monomers were added per p(DMAc$_{119}$-*b*-APTAC$_{43}$). In both cases, spheroidal particles with an inner concentric ring structure proved to be the predominant particle morphology. Images were taken from the same TEM grid at different locations and provide a representative view of the complete sample.

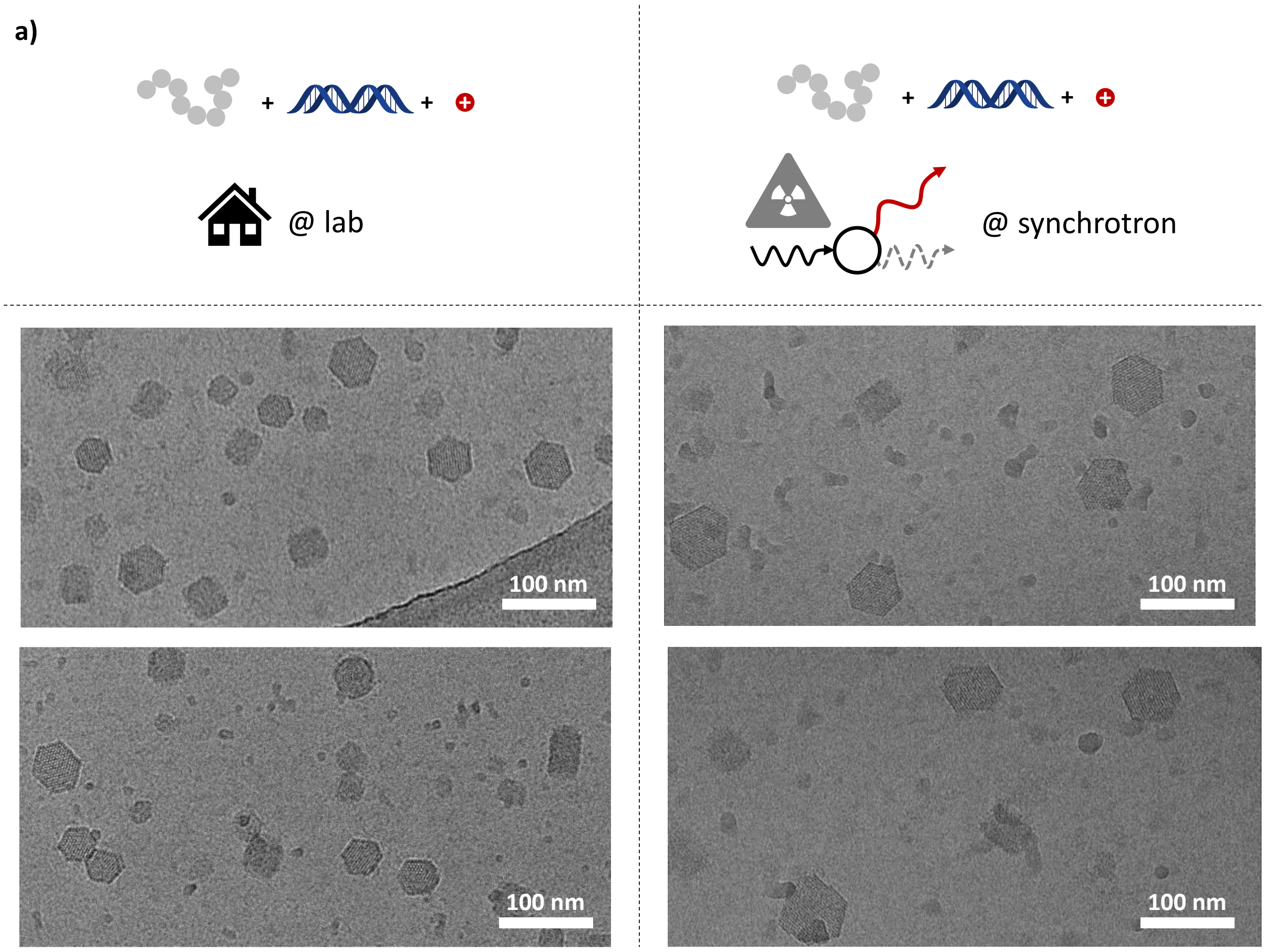

Figure S9. Direct comparison between the polymerization-induced electrostatic self-assembly (PIESA)-derived particles prepared **a)** in our wet chemical lab and **b)** at the synchrotron facility. Despite performing the reaction at the synchrotron on a larger volume (3 mL *vs*. 300 µL) and having a continuous outflux of reaction volume into the SAXS measurement capillary, hexagonal disk-shaped particles with distinct hexagonal internal ordering of the double-stranded DNA cargo (dsDNA) were obtained in both cases as confirmed with cryogenic transmission electron microscopy (cryo-TEM). Note: the amorphous features in panel b) are related to poor ice quality which can occur during snap freezing of the samples. Images were taken from the same TEM grid at different locations and provide a representative view of the complete sample.

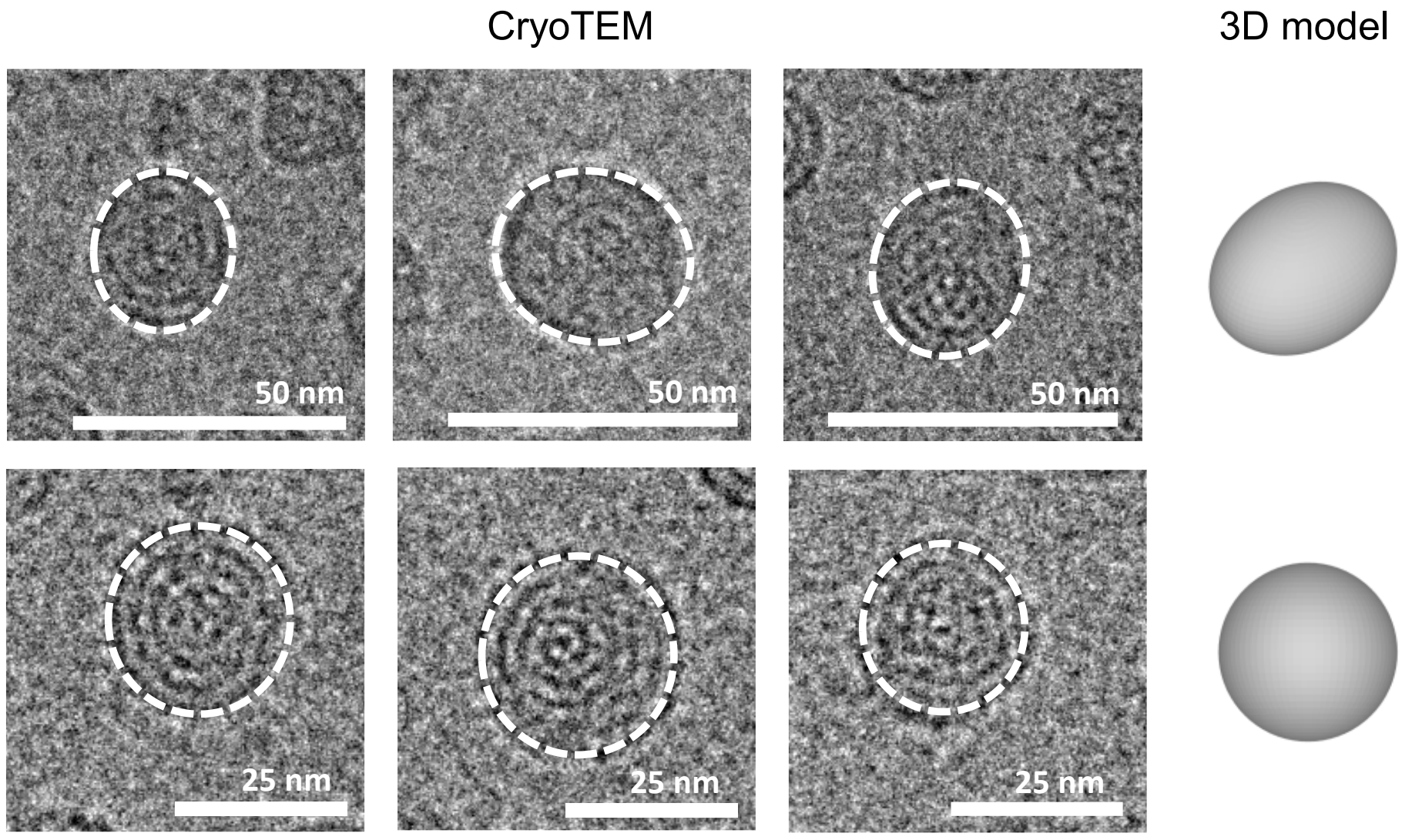

Figure S10. Representative cryogenic transmission electron microscopy (cryo-TEM) image of spiral-like particles prepared by direct mixing. Depending on the particle orientation withing the frozen layer of the cryo-TEM sample, the particles either appear egg-shaped (top row) or spherical (bottom row). The dependence of the 2D projection on particle orientation suggests that the particles have an elliptical shape, which is in agreement with SAXS fitting (see **Section S5.3**) The 3-dimensional model represents an ellipsoid with an aspect ratio of 1.3.

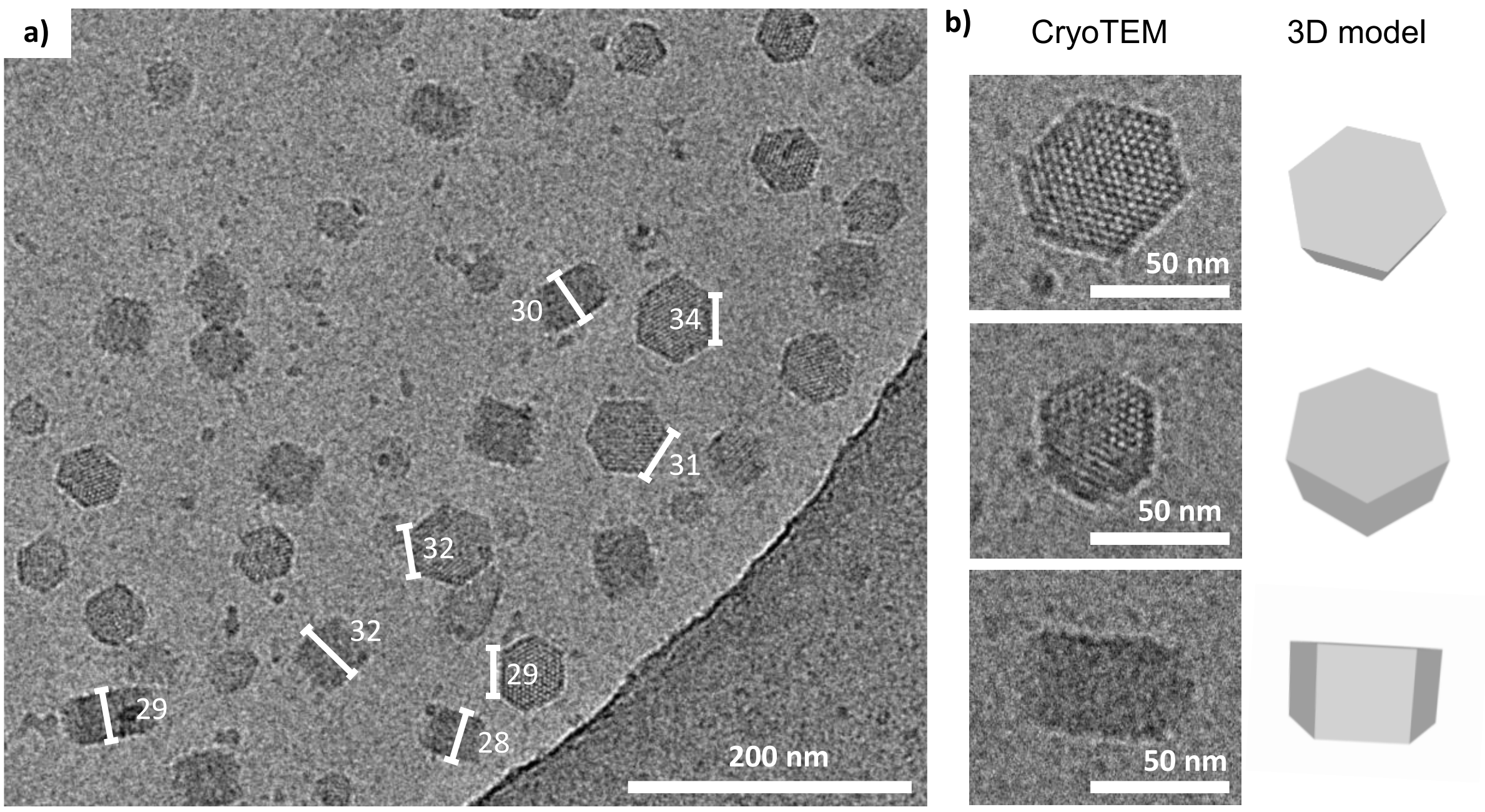

Figure S11. Representative cryogenic transmission electron microscopy (cryo-TEM) image of hexagonal disk-shaped particles prepared by polymerization-induced electrostatic self-assembly (PIESA). In addition to the distinct hexagons, the image features rectangular shapes representing the side view of the particles. The numbers in this figure reflect measured edge lengths and thicknesses of the hexagons in nanometers, revealing an average edge length of 26.0 ± 8.8 nm and an average thickness of 32.8 ± 6.2 nm. This thickness is in reasonable agreement with the dimension extracted from SAXS fitting (see **Section S5.4**). The effect of variations in particle orientations within the frozen layer of the cryo-TEM sample is illustrated by mapping commonly observed particle appearances onto a 3-dimensional model of a hexagonal disk (thickness = 90% of edge length).

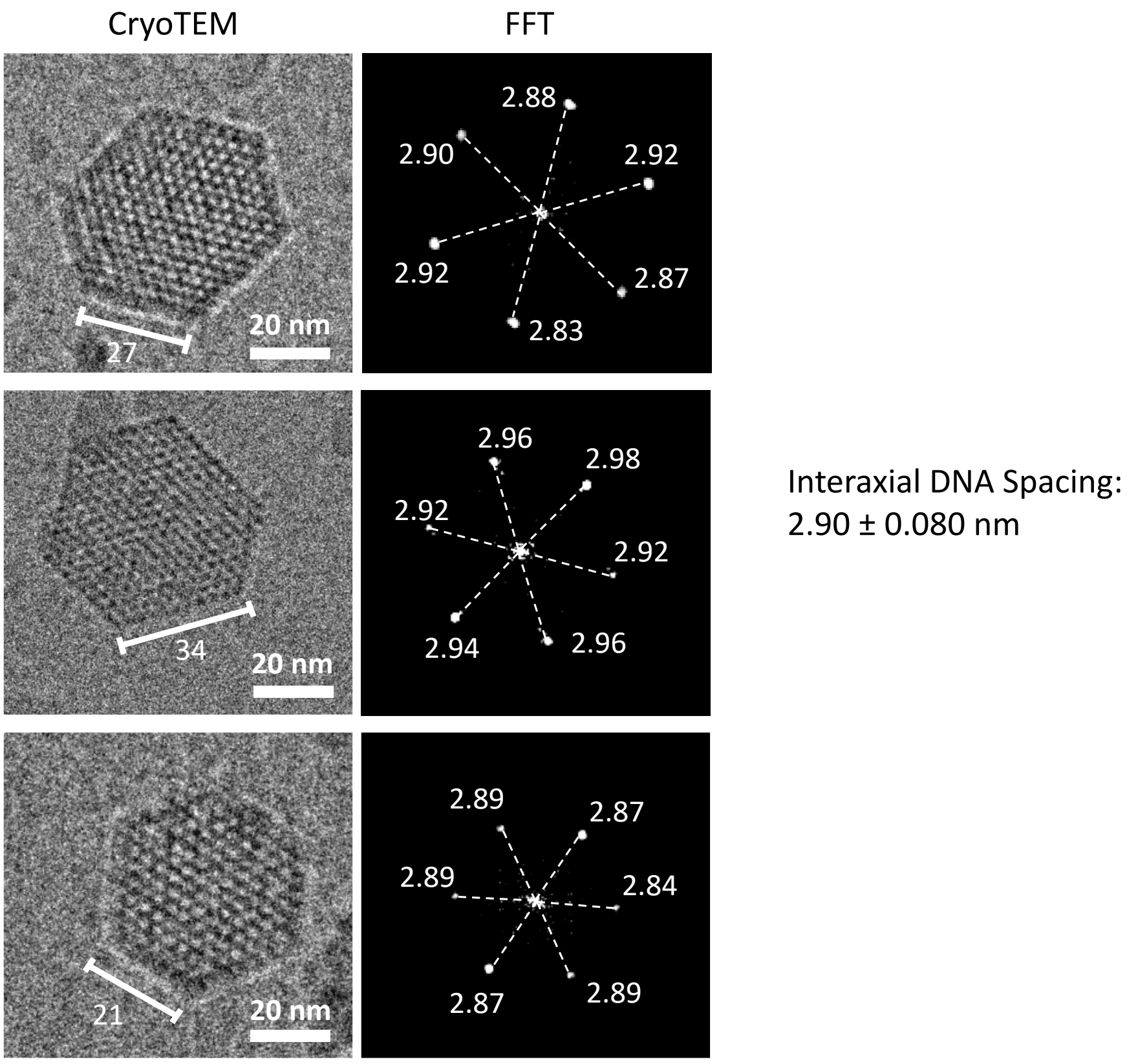

Figure S12. Three representative, high magnification cryogenic transmission electron microscopy (cryo-TEM) images (left) and their corresponding Fast Fourier Transform (FFT) representations (right) of hexagonal disks obtained by polymerization-induced electrostatic self-assembly (PIESA). For all three particles, hexagonally patterned signals were observed in the FFTs. Measuring the distance between high intensity signals (dotted lines) provides an average interaxial double-stranded DNA (dsDNA) spacing within these particles. An average value of 2.9 ± 0.08 nm was found.

S4. Experimental setups

S4.1 Custom-made LED-Photoreactor

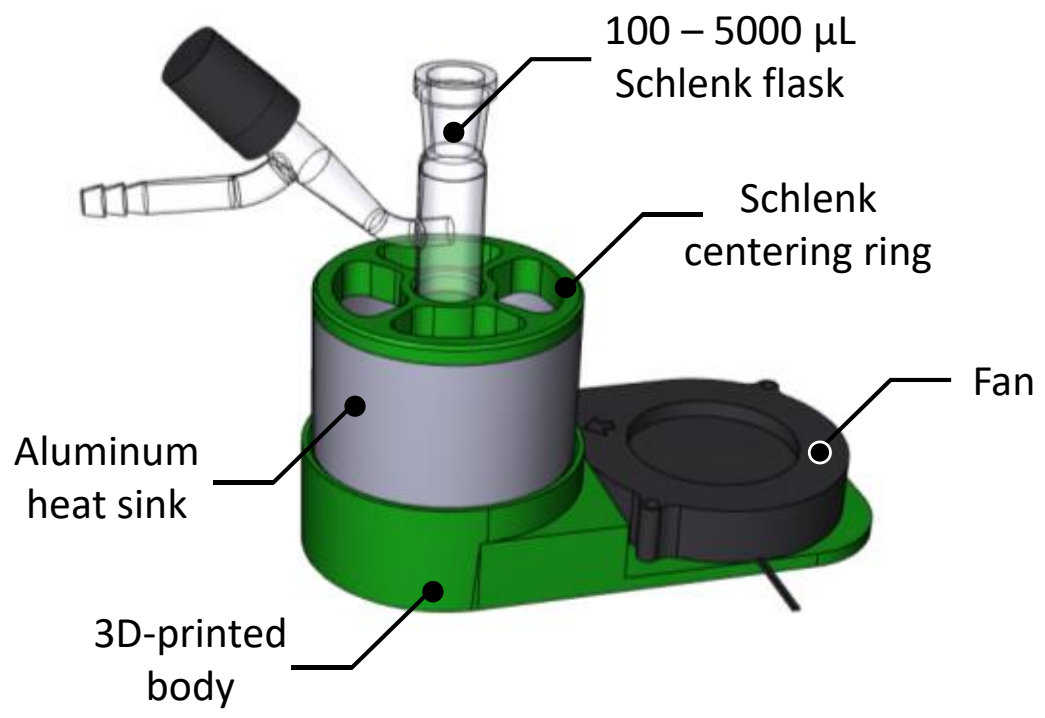

Figure S13. Schematic representation of the LED-photoreactor used for the synthesis of poly(*N*,*N*-dimethylacrylamide-*b*-(3-acrylamidopropyl)trimethylammonium chloride) p(DMAc-*b*-APTAC$_x$) block copolymers (BCPs; **Section S1.4**) and during PIESA (**Section S1.5**). The design features a 3D-printed body fitted with an aluminum cylinder lined with LEDs (λ_{max} = 365 nm, see **Section S1.4** for details). A Schlenk flask (volume = 100 – 5000 µL) is positioned in the middle of the cylinder using a centering ring (distance between LEDs and reaction flask ≈ 3 cm). The aluminum cylinder acts as a heat sink to counteract heating of the LEDs. Irradiative heating of the sample is further suppressed by an electric fan that blows air from the bottom into the aluminum cylinder.

S4.2 Set-ups used for Time-Resolved Small Angle X-Ray Scattering experiments

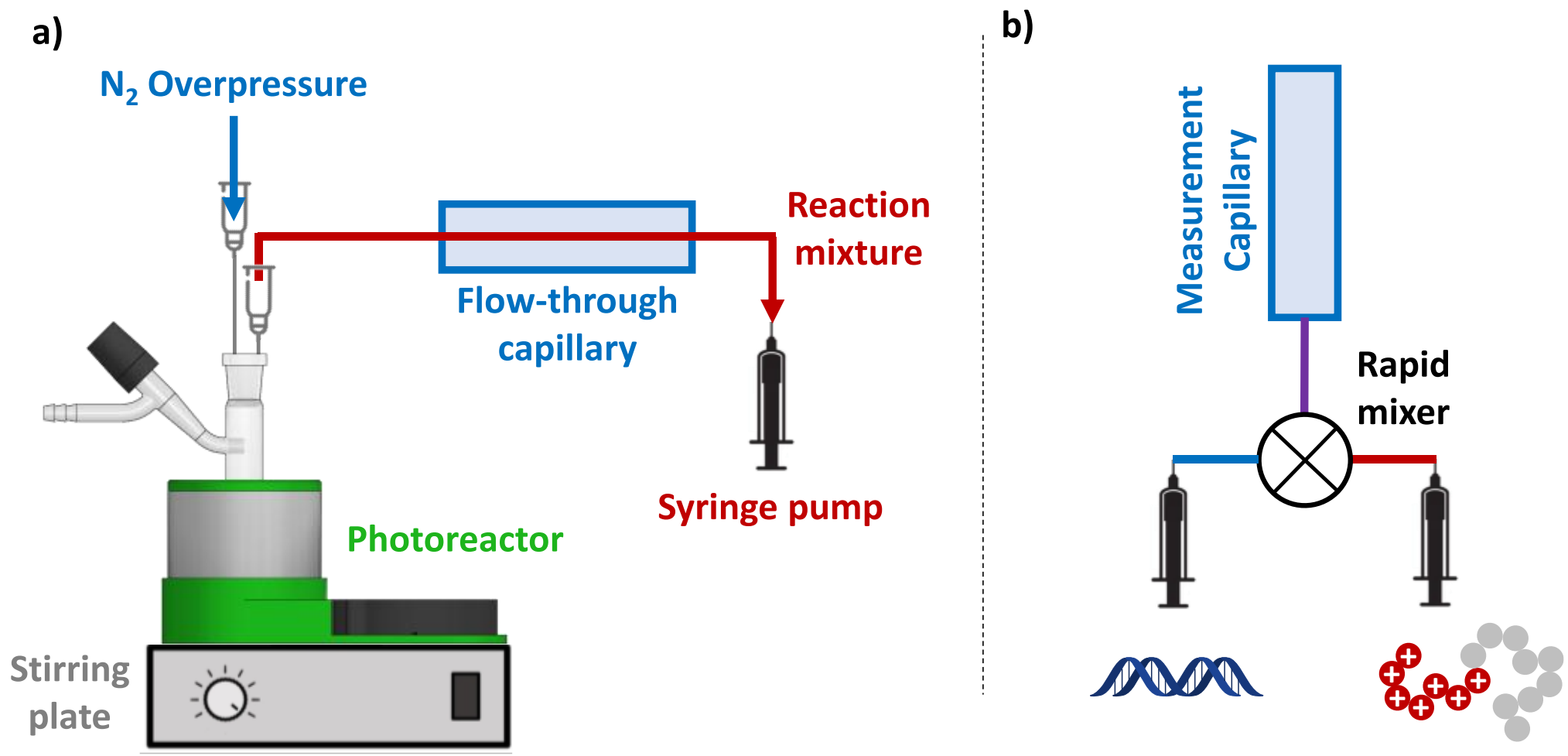

Figure S14. a) Schematic overview of the set-up used to monitor particle formation during polymerization-induced electrostatic self-assembly (PIESA) with time-resolved small-angle X-ray scattering (TR-SAXS). Degassed reaction mixture was placed in an LED photoreactor (**Figure S13**). After initiating the reaction and under continuous magnetic stirring, a syringe pump was activated that allowed for the constant withdrawal of reaction mixture (red arrow) into the SAXS flow-through capillary (blue). Nitrogen overpressure (blue arrow) was supplied to minimize the risk of oxygen contamination. **b)** Schematic representation of the stopped-flow sample environment to study the direct mixing process with TR-SAXS. Two separate syringes were filled with either a solution containing the double-stranded DNA template (blue) or the preformed poly(*N*,*N*-dimethylacrylamide-*b*-(3-acrylamidopropyl)trimethylammonium chloride) p(DMAc-*b*-APTAC$_{50}$) block copolymer (BCP, red). Both solutions are simultaneously injected in a rapid mixer, after which the mixed solution (purple) enters a SAXS measurement capillary. See **Section S2.5** for further experimental details on TR-SAXS measurements.

S5. SAXS Data Analysis

S5.1 Bragg Peak analysis: Peak fitting and integration. The evolution of the Bragg peak at high q was analyzed and quantified using a summation of a Gaussian peak and a power law model:

$$I(q) = f_s \left(f_a \, e^{\frac{-0.5(q-q_0)^2}{\sigma^2}} + f_b q^{-p} \right) + \text{background} \quad \textbf{Eq. S5}$$

f_s	Total scaling factor
f_a	Scaling factor for Gaussian Peak model
f_b	Scaling factor for Power Law model
q_0	Position of Bragg peak (nm^{-1})
p	Power exponent
σ	Standard Deviation in Bragg peak (nm^{-1})

To obtain the full width half maximum (FWHM) of the Bragg peak, the standard deviation obtained from the fit was multiplied by 2.354.

The area under the Bragg peak, a measure for the dsDNA ordering and encapsulation, was calculated after fitting a baseline to the curves described by **Eq. S5** and calculating the enclosed area (**Figure S15**, shaded regions) via numerical integration in Origin 9.1. Baselines (**Figure S15**, black lines) were created by defining several anchoring points on both sides of the Bragg peak and connecting these points via a BSpline interpolation method.

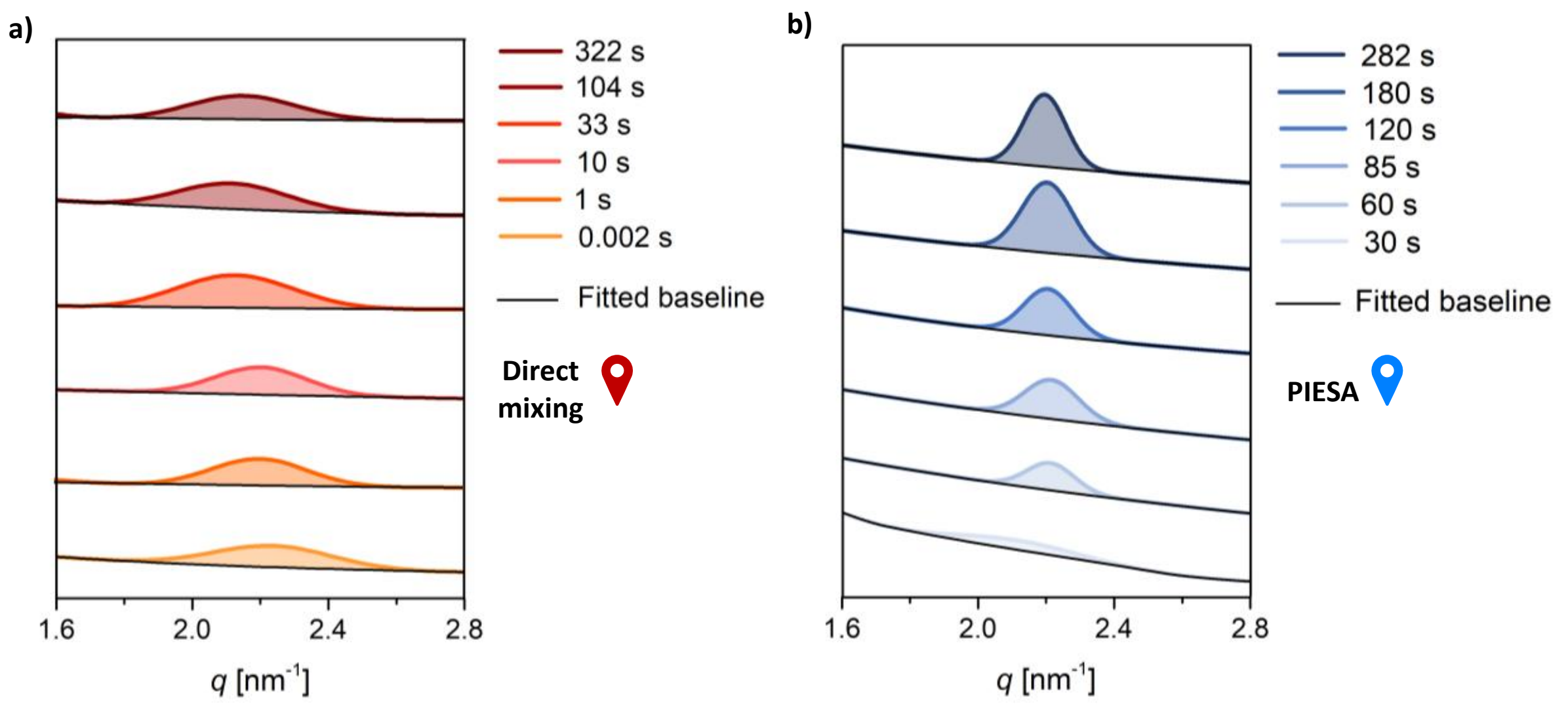

Figure S15. Fits (**Eq. S5**, colored lines) to the Bragg peaks originating from internal ordering of the double-stranded DNA (dsDNA) templates as measured with time-resolved small-angle X-ray scattering (TR-SAXS) for particle dispersions prepared *via* **a)** direct mixing and **b)** PIESA. For all time points, baselines (black lines) were fitted, and the enclosed areas (shaded colors) were calculated *via* numerical integration.

S5.2 Determining the radius of gyration (R_g) via Guinier analysis. The radii of gyration (R_g's) of the particles for both assembly routes were obtained using the Guinier approximation on the final collected SAXS frames.[4] It was assumed that the particles were small enough (form factor $P(q) \approx 1$) and that the dispersions were sufficiently diluted (structure factor $S(q)$ = 1). Following Guinier's formalism (**Eq. S6**), ln($I(q)$) was plotted against q^2. The slope of a linear fit in the low q-regime ($qR_g < 1.3$) equals $R_g^2/3$ (**Figure S16**) and gives therefore direct access to R_g.

$$\ln(I(q)) = \ln(I_0) - \frac{R_g^2}{3} q^2 \qquad \textbf{Eq. S6}$$

where I_0 is the forward scattering (scattering at 0 angle). To increase accuracy of extrapolation to low q values, $q \rightarrow 0$, the scattering profiles obtained at a detector distance of 1.5 m was supplemented with data recorded on a 10 m detector. It should be noted that Guinier's formalism is strictly only valid for particles with a homogeneous scattering length density (SLD). However,

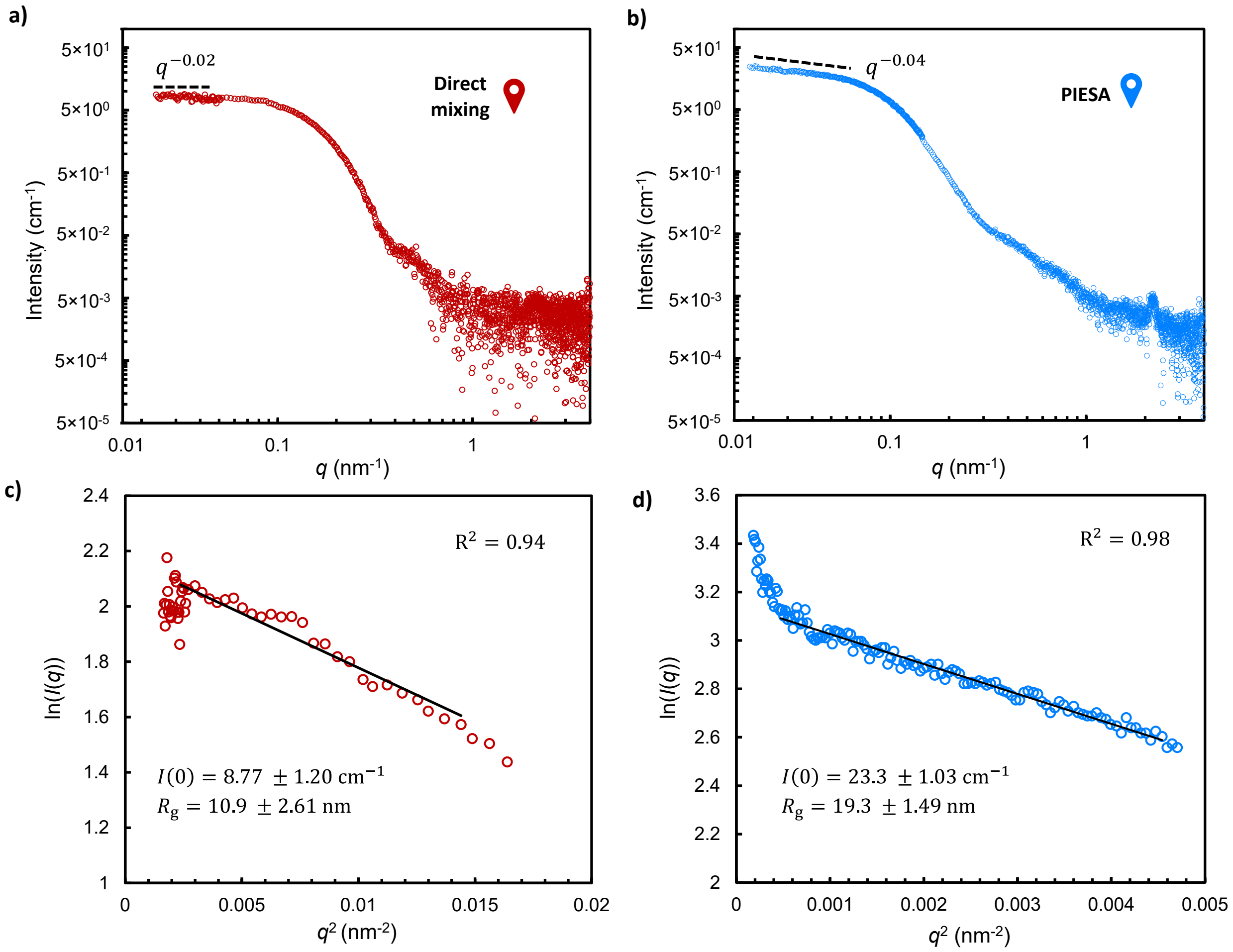

Figure S16. Merged small-angle X-ray scattering (SAXS) profiles measured at detector distances of 1.5 and 10 m for particle dispersion obtained by **a)** direct mixing and **b)** PIESA. The indicated slopes at the low q-regime were obtained by fitting a power law to the experimental data. Guinier analysis of **c)** direct mixing and **d)** PIESA-derived particles based on the scattering profiles shown in Panels a) and b), respectively. Radii of gyration (R_g's) were obtained from the slope of the Guinier fit. The deviating points at low q^2 are the result of imperfect background subtraction and/or detector stitching. These points were excluded from fitting.

since we anticipate that the nanoparticles in this study have a weakly scattering stabilizing corona and that dsDNA is the main contributor to the scattered intensity, an effective radius of gyration weighted by contrast, $R_{g,eff}$, is obtained and reported.

Following the Guinier analysis, R_g was determined for all spectra shown in **Figure 2b** of the **Main Text** (PIESA-derived hexagonal disks). **Figure 3e** of the **Main Text** depicts a power law scaling of $R_g \propto t^{0.17}$ by fitting a power law to the data. Linearizing the data by plotting log(t) *vs.* log(R_g) and fitting the obtained data with a linear curve reveals the same scaling behavior (**Figure S17**).

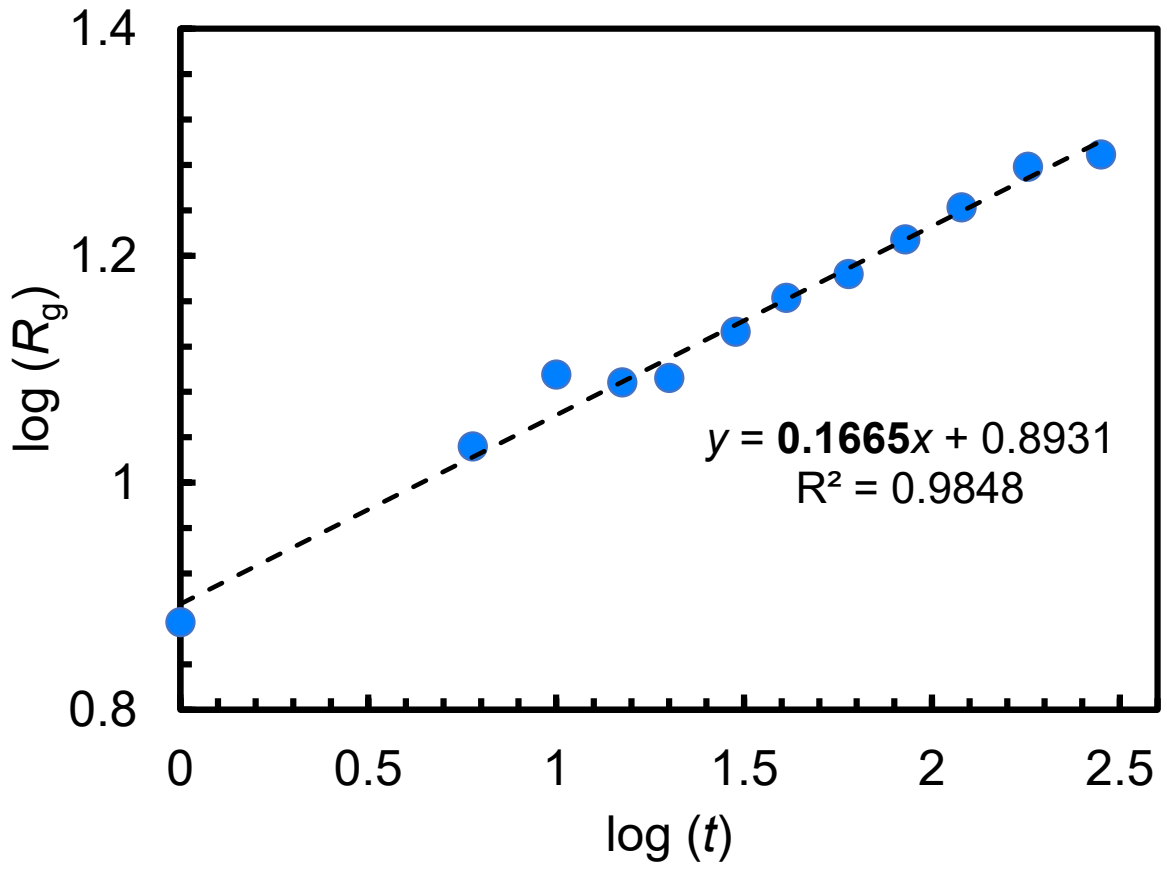

Figure S17. Time evolution of the radius of gyration (R_g) during the PIESA process yielding hexagonal disk-shaped particles plotted on a log-log scale. The power law scaling of $R_g \propto t^{0.17}$ shown in **Figure 3e** of the **Main Text** was recovered by fitting the linearized data with a straight line.

S5.3 Direct mixing: Form factor fitting of SAXS data. The particles obtained by direct mixing were fitted using a form factor for ellipsoidal particles. This choice was motivated by cryo-TEM analysis (see **Figure S10**), which showed that the particles either appear circular or egg-shaped depending on their orientation during imaging. Additionally, converting the measured

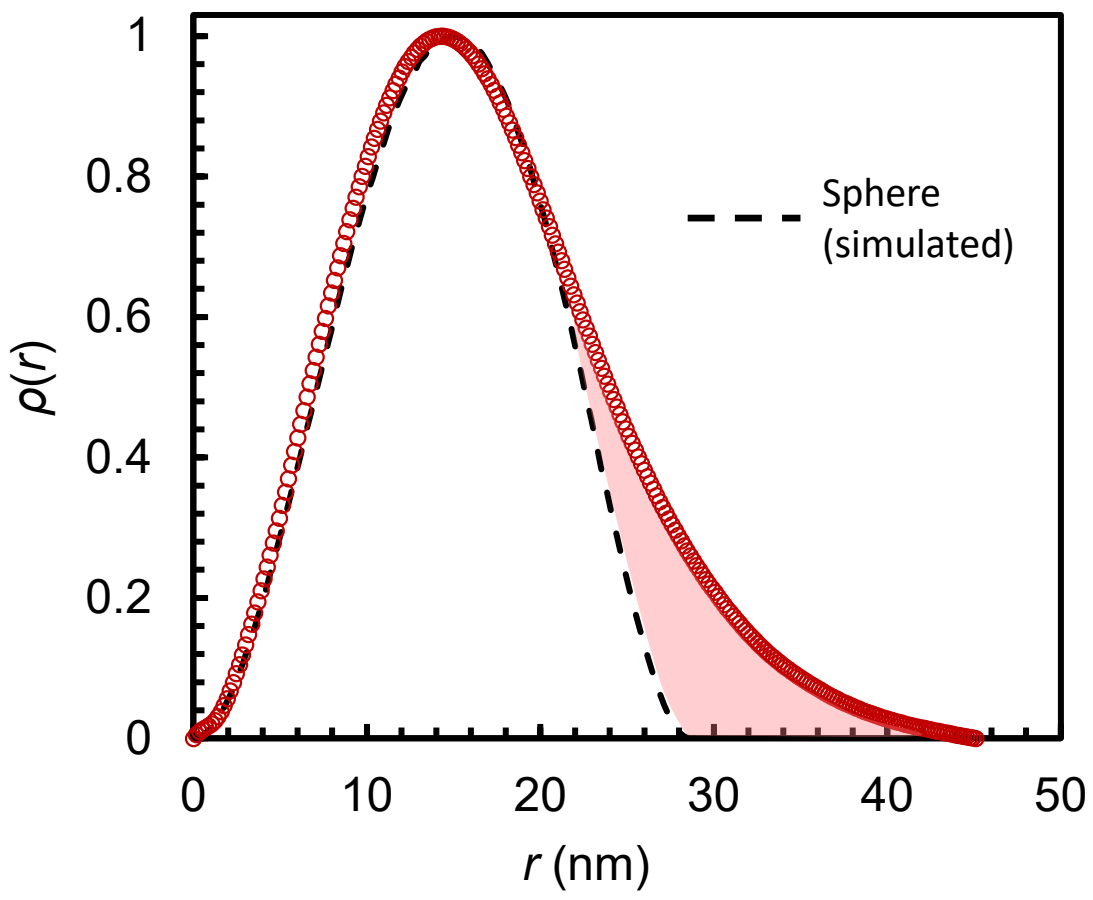

Figure S18. Experimentally determined pair distribution function ($\rho(r)$) for particles prepared via the direct mixing approach. Compared to the simulated $\rho(r)$ for a perfect spherical particle, the experimental $\rho(r)$ features an asymmetric shape, highlighted by the pink shaded area, signaling particle anisotropy.

scattering profile of these particles to a pair distribution function ($\rho(r)$), yielded asymmetric curves, indicative for particle anisotropy (**Figure S18**). As reference, a simulated $\rho(r)$ for a perfect sphere was overlayed to the experimentally obtained function (**Figure S18**, dotted line).

Fitting of the experimental SAXS profiles was performed using SasView[5] using the standard ellipsoidal model. Here, the scattered intensity is given by

$$I(q,\alpha) = \frac{f_\mathrm{e}}{V_\mathrm{el}} F^2(q,\alpha) + \mathrm{background} \qquad \textbf{Eq. S7}$$

with f_e as the scaling factor, V_el as the ellipsoidal volume and $F(q,\alpha)$ as the form factor amplitude which can be described as

$$F(q,\alpha) = \Delta\rho V_\mathrm{el} \frac{3(\sin qr - qr\cos qr)}{(qr)^3} \qquad \textbf{Eq. S8a}$$

with

$$r = \left(r_\mathrm{e}^2 \sin^2\alpha + r_\mathrm{p}^2 \cos^2\alpha\right)^{0.5} \qquad \textbf{Eq. S8b}$$

where $\Delta\rho$ represents the scattering length density (SLD) of the particles with respect to the solvent, and r_e and r_p are the equatorial and polar radius of the ellipsoid, respectively. The ellipsoidal volume, V_el, can be described with $V_\mathrm{el} = \frac{4}{3}\pi r_\mathrm{p} r_\mathrm{e}^2$. α is the orientation angle of the ellipsoid. For random orientation of the nanoparticles with respect to the incoming X-rays, the form factor is given by $P(q) = F^2(q) = \int_0^{0.5\pi} F^2(q,\alpha)\sin(\alpha)\, d\alpha$.

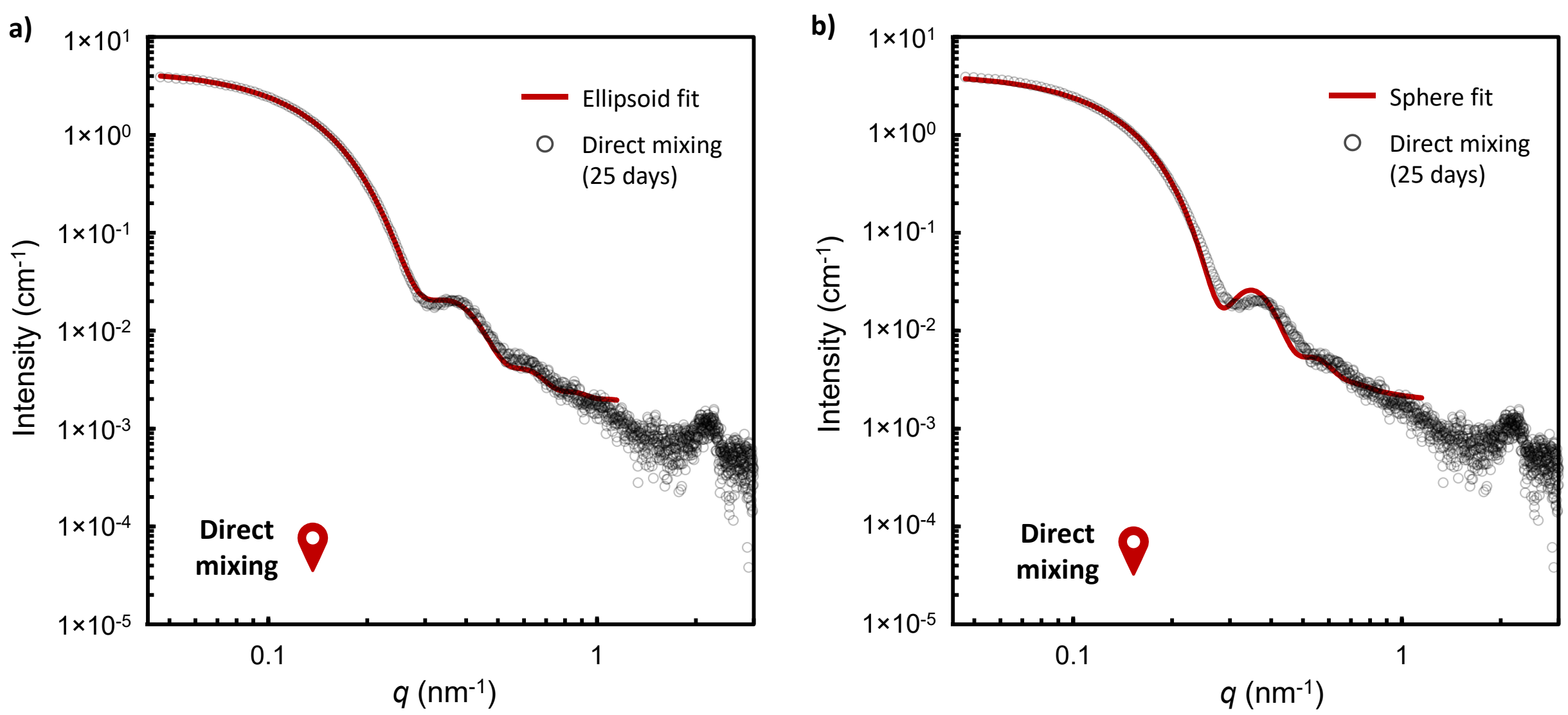

Figure S19. Small-angle X-ray scattering (SAXS) profiles measured for particles prepared *via* the direct mixing protocol (black circles). The particles were equilibrated for 25 days (RT, ([+] = [-] = 5 mM). The experimental data was fitted either with an **a)** ellipsoidal or **b)** a polydisperse spherical form factor model (red lines). Only the ellipsoidal model was able to capture all features in the scattering profile. Note that fitting was restricted to $0.047 < q < 1.15$ nm^{-1}. Higher q-values were omitted from the fit because no shape information is present in this region.

The scattering profiles were fitted from $0.047 < q < 1.15$ nm^{-1}. The high q region was omitted as it contains no contributions of the overall particle shape. As shown in **Figure S19a**, the fit (red line) could accurately describe the experimentally measured data. The values follow from the fit can be found in **Table S1**.

Table S1. Fitting parameters obtained for fitting direct mixing-derived particles with an ellipsoidal form factor model.

f_e	0.48 ± 0.00033
background	0.0018 ± 3.3 · 10^{-5} cm^{-1}
r_p	13.36 ± 0.11 nm
r_e	17.21 ± 0.17 nm
polydispersity in r_e	0.185 ± 0.008
χ^2	1.75

Plugging the values of r_p and r_e yields an elliptical particle volume of

$$V_{el} = \frac{4}{3}\pi r_p r_e^2 = \frac{4}{3}\pi \cdot 13.36 \text{ nm} \cdot (17.21 \text{ nm})^2 \approx 16575 \text{ nm}^3 \quad \textbf{Eq. S9}$$

As reference, an attempt was made to fit the data with a form factor model describing polydisperse spheres. In essence the applied form factor model is identical to the ellipsoidal model described above, with the exceptions that α is omitted and r_p and r_e are both equal to the radius of the spherical particle. The best possible fit is depicted in **Figure S19b**, and the corresponding fitting values are listed in **Table S2**.

Table S2. Fitting parameters obtained for fitting direct mixing-derived particles with a spherical form factor model.

f_s	0.47 ± 0.00028
background	0.0019 ± 3.3 · 10^{-5} cm^{-1}
r	15.55 ± 0.0048 nm
polydispersity in r	0.11 (fixed)
χ^2	19.97

As anticipated based on the asymmetric $\rho(r)$, the spherical model does not capture all features of the scattering profiles. In addition to a slight misfitting of the slope at low q, the sphere model is unable to capture the first oscillation point regardless of the polydispersity that was introduced. The limited fitting accuracy of is also reflected by a χ^2 value that is significantly higher than the one found for the ellipsoidal model (**Table S1**).

S5.4 PIESA: Form factor fitting of SAXS data. The SAXS profile of the particles obtained after PIESA was fitted with a standard cylinder model in SasView.[5] Although not capturing the fine details of the hexagonal morphology, cylinder models have been used to fit colloids with

comparable shapes, *e.g.*, hexagonal nanoplatelets.[6] For the cylindrical model, the scattered intensity is described as

$$I(q,\alpha) = \frac{f_c}{V_{\text{cyl}}} F^2(q,\alpha) + \text{background} \qquad \textbf{Eq. S10}$$

where f_c is a scaling factor, V_{cyl} as the cylinder volume and $F(q,\alpha)$ as the form factor amplitude which can be written as

$$F(q,\alpha) = 2(\Delta\rho)V_{\text{cyl}} \frac{\sin(0.5\, qL\cos\alpha)}{0.5\, qL\cos\alpha} \frac{J_1(qR_c \sin\alpha)}{qR_c \sin\alpha} \qquad \textbf{Eq. S11}$$

with $\Delta\rho$ representing the SLD of the particles with respect to the solvent and J_1 the first order Bessel function. R_c and L describe the radius and length of the cylinder, respectively. Evidently, L is directly related to the thickness of the hexagonal disks (see **Figure S11**). The cylinder volume, V_{cyl}, can be readily calculated by $V_{\text{cyl}} = \pi R_c^2 L$. α is the orientation angle of the cylinder, where for random orientation of the nanoparticles, the form factor is given by $P(q) = F^2(q) = \int_0^{0.5\pi} F^2(q,\alpha)\sin(\alpha)\, d\alpha$.

The scattering profile was fitted from 0.047< q < 0.9 nm^{-1}. The high q region was omitted as it contains no contributions of the overall particle shape. As shown in **Figure S20**, the fit (blue line) could accurately describe the experimentally measured data, despite ignoring the hexagonal cross-section of the particles. The values follow from the fit can be found in **Table S3**.

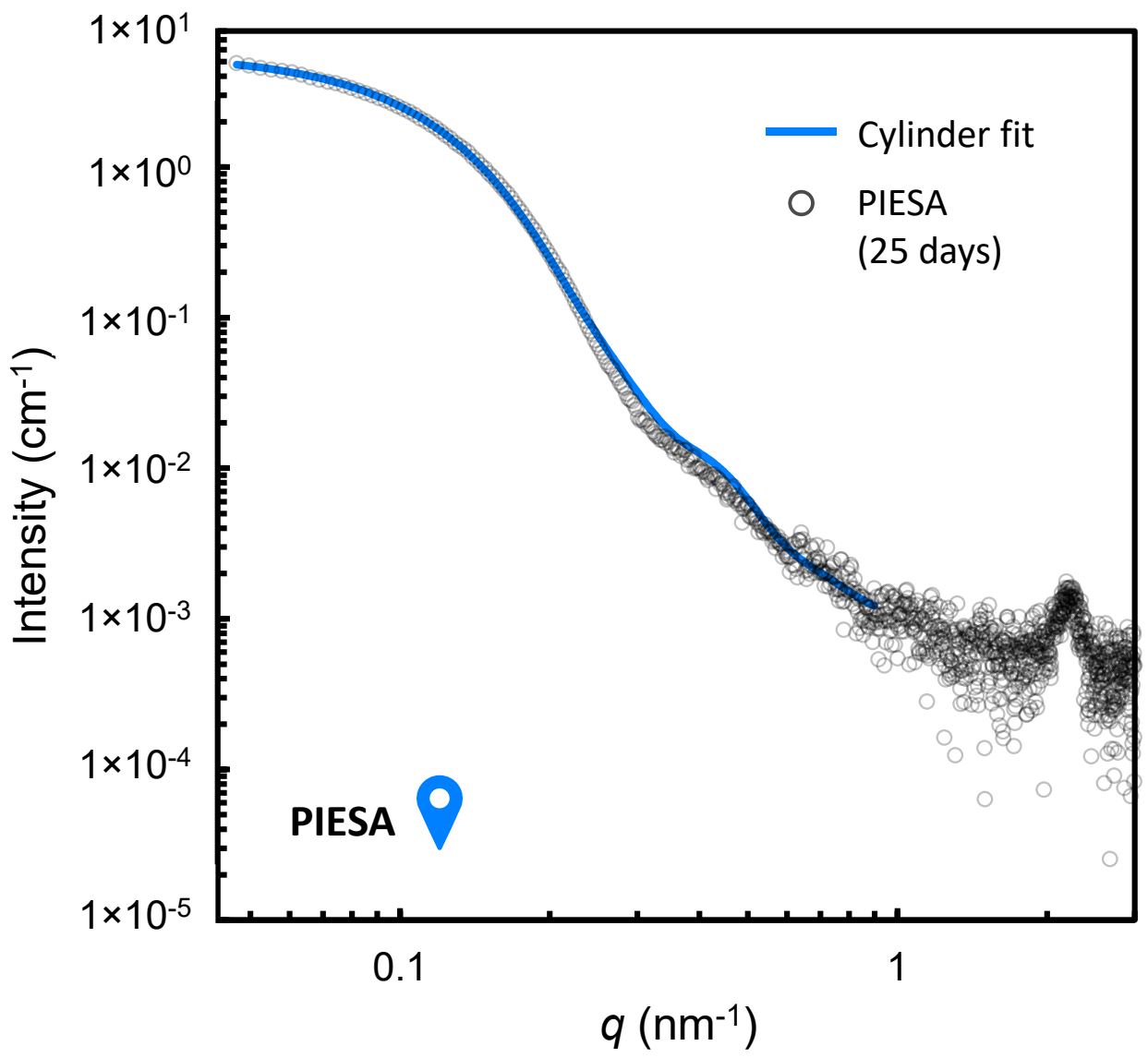

Figure S20. Small-angle X-ray scattering (SAXS) profiles measured for particles prepared *via* PIESA (black circles). The particles were equilibrated for 25 days (RT, ([+] = [-] = 5 mM). The experimental data was fitted with a polydisperse cylinder form factor model (blue line). Note that fitting was restricted to 0.047 < q < 0.9 nm^{-1}. Higher q-values were omitted from the fit because no shape information is present in this region.

Table S3. Fitting parameters obtained for fitting PIESA-derived particles with a cylindrical form factor model.

f_c	0.47 ± 0.00030
background	$7.5 \cdot 10^{-4}$ cm^{-1} (fixed)
R_c	17.74 ± 0.016 nm
polydispersity in R_c	0.19 (fixed)
L	20.42 ± 0.026 nm
polydispersity in L	0.15 (fixed)
χ^2	14.40

Plugging the values of L and R_c yields a cylindrical particle volume of

$$V_{\text{cyl}} = \pi\, R_c^2\, L = \pi\,(17.74\text{ nm})^2 \cdot 20.42\text{ nm} \approx 20189\text{ nm}^3 \qquad \textbf{Eq. S12}$$

Correcting for the hexagonal cross-section provides an estimation for the volume of the disk-shaped particle:

$$V_{\text{hex}} = \frac{3\sqrt{3}}{2}\, R_c^2\, L = \frac{3\sqrt{3}}{2}\,(17.74\text{ nm})^2 \cdot 20.42\text{ nm} \approx 16696\text{ nm}^3 \qquad \textbf{Eq. S13}$$

S5.5 dsDNA: Form factor fitting of SAXS data. A pure solution of double-stranded DNA (dsDNA) was measured with static SAXS (see **Section S2.6**) to confirm that these short oligonucleotides behave as rigid rods in solution. Based on literature, a cylindrical shape was anticipated,[7-8] making the use of the same cylindrical form factor model as described in **Section S5.4** appropriate. The best attainable fit (blue solid line) to the experimental scattering profile (black circles) is shown in **Figure S21**.

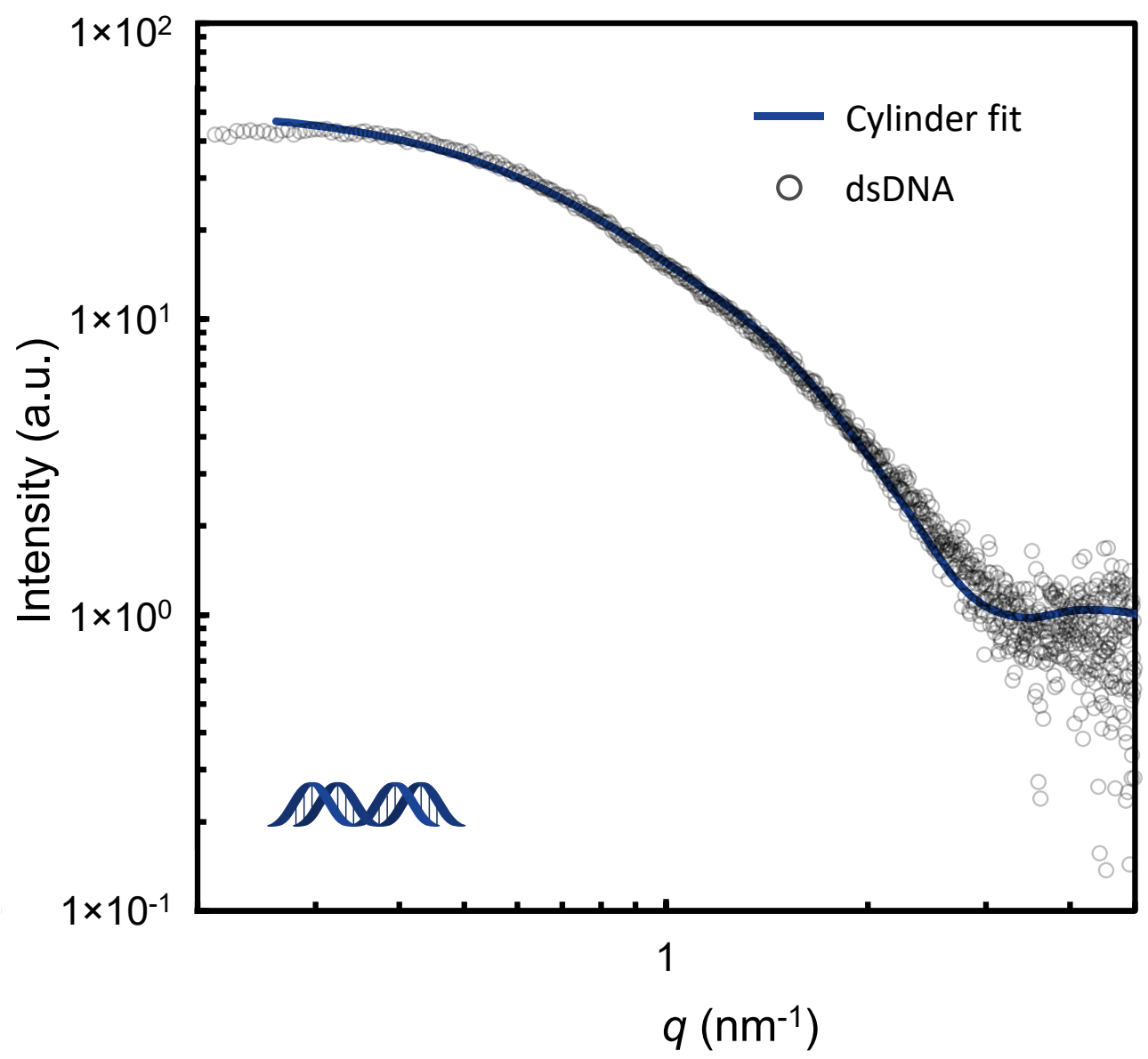

Figure S21. Small-angle X-ray scattering (SAXS) profiles measured for a solution of pure double-stranded DNA (dsDNA, black circles). The experimental data was fitted with a monodisperse cylinder form factor model (blue line).

Table S4. Fitting parameters obtained for fitting double-stranded DNA (dsDNA) with a cylindrical form factor model.

f_c	16715 ± 72
background	0.95 ± 0.013 (a.u.)
R_c	1.15 ± 0.0031 nm
Polydispersity in R_c	0 (fixed)
L	7.33 ± 0.038 nm
Polydispersity in L	0 (fixed)
χ^2	1.73

The cylinder model provided a high accuracy fit with the experimental data. The extracted values of L and R_c are in perfect agreement with values found in literature for short DNA and siRNA,[9-11] providing confidence in the fit and obtained structural features.

S5.6 Calculating the particle's apparent molecular weight. The particle molecular weight (M_{particle}) can be calculated using the scattering intensity at $q \rightarrow 0$ (obtained from Guinier plot, **Figure S16**) and is given by[12]

$$I_0 = \frac{M_{\text{particle}} c_{\text{total}} (\rho_{\text{bb}} - \rho_{\text{solv}})^2 \, v_0^2}{N_{\text{a}}} \qquad \textbf{Eq. S14}$$

where I_0 is the scattering intensity at $q \rightarrow 0$ (cm^{-1}) (**Figure S16**), c_{total} the total particle weight concentration (g · cm^{-3}), ρ_{bb} and ρ_{solv} the scattering length densities (SLDs) of the building block and the solvent (cm^{-2}), v_0 represents the building block specific volume (cm^3 · g^{-1}), and N_{a} equals Avogadro's number (mol^{-1}). A building block is defined as the smallest hypothetical charge neutral complex that can be formed upon electrostatic complexation of p(DMAc$_{119}$-*b*-APTAC$_x$) and the dsDNA template. For direct mixing p(DMAc$_{119}$-*b*-APTAC$_{50}$) was used, implying 1.136 DNA strands are required for reaching the point of charge stoichiometry (c_{total} = 3.56 · 10^{-3} g/cm^3). The PIESA pathway yielded p(DMAc$_{119}$-*b*-APTAC$_{43}$) BCPs that require 0.977 dsDNA strands to neutralize the cationic charges. The total particle weight concentration was corrected for the residual 15% of monomer that remained in the sample after the reaction (c_{total} = 3.45 · 10^{-3} g/cm^3). The building block SLD and v_0 were calculated by taking the weighted fraction of each individual species of which the building block comprises. The following SLDs and $v_0's$ used for direct mixing and PIESA samples can be found in **Table S5** and **S6**, respectively.

Table S5. Scattering length densities (SLDs) and specific volumes ($v_0's$) for direct mixing samples

Direct Mixing	M_w (g · mol-1) (no counterions)	SLD (10^{10} cm-2)	v_0 (cm3 · g-1)	Weight fraction in building block (-)
D_2O*a, [12]	20.03	9.39	0.905	-
p(DMAc)[13-14]	99.13	10.72	0.846	0.33
p(APTAC)*b, [15-16]	171.26	10.33	0.766	0.24
dsDNA[1, 17]	13466.9	15.59	0.56	0.43
p(DMAc$_{119}$-*b*-APTAC$_{50}$) + 1.136 dsDNA	35657.9	12.72	0.70	1

Table S6. Scattering length densities (SLDs) and specific volumes ($v_0's$) for PIESA samples

PIESA	M_w (g · mol-1) (no counterions)	SLD (10^{10} cm-2)	v_0 (cm3 · g-1)	Weight fraction in building block (-)
D_2O*a, [12]	20.03	9.39	0.905	-
p(DMAc)[13-14]	99.13	10.72	0.846	0.36
p(APTAC)*b, [15-16]	171.26	10.33	0.766	0.22
dsDNA[1, 17]	13466.9	15.59	0.56	0.42
p(DMAc$_{119}$-*b*-APTAC$_{43}$) + 0.977 dsDNA	32317.8	12.61	0.71	1

*a The SLD of D_2O was calculated in SasView
*b The partial specific volume of poly((3-methacrylamidopropyl)trimethylammonium) was used

Plugging the values listed in **Table S5** and **S6** into **Eq. 14** yields M_{particle} and the corresponding aggregation numbers for both BCPs (N_{BCP}) and dsDNA (N_{DNA}) (**Table S7**).

Table S7. Particle molecular weights ($M_{particle}$) and aggregation numbers for BCPs (N_{BCP}) and dsDNA (N_{DNA}) for particles prepared via direct mixing and PIESA.

	M_{particle} (g · mol-1)	Number of building blocks / particle N_{BCP} (-)	Number of dsDNA / particle N_{DNA} (-)
**Direct mixing**	$2.7 \cdot 10^6 \pm 0.37 \cdot 10^6$	76 ± 10	86 ± 12
**PIESA**	$7.7 \cdot 10^6 \pm 0.34 \cdot 10^6$	240 ± 11	234 ± 10

The packing density of the DNA per cubic nanometer can be obtained by normalizing the values for N_{DNA} per particle by the particle volumes obtained from the SAXS fits as described in **Section S5.3** and **S5.4**

Direct Mixing: $$\frac{86}{V_{\text{el}}} = \frac{86}{16575\ \text{nm}^3} = 5.2\ \cdot 10^{-3} \pm 7.2\ \cdot 10^{-4}\ \text{nm}^{-3} \qquad \textbf{Eq. S15a}$$

PIESA: $$\frac{234}{V_{\text{cyl}}} = \frac{234}{20189\ \text{nm}^3} = 11.6\ \cdot 10^{-3} \pm\ 5.4\ \cdot 10^{-4}\ \text{nm}^{-3} \qquad \textbf{Eq. S15b}$$

Compared with particles prepared by direct mixing, the internal hexagonal dsDNA ordering of the PIESA particles enables a 2.23-fold increase in packing density.

S5.7 Number of layers and thickness of the hexagonal PIESA particles. Assuming the hexagonal disks consist of layers of hexagonally packed dsDNA resembling a smectic liquid crystal, the number of crystal layers was estimated based on three different methods:

(i) *Directly measuring the dimensions of side views shown in cryo-TEM.* The procedure was previously shown in **Figure S11**. Although difficult to judge if the disks are imaged fully side-on, measuring a larger number of 22 particles yielded an average thickness of 32.8 ± 6.2 nm. Assuming that one crystal layer has approximately the same height as the length of a single dsDNA (= 7.5 nm as measured with SAXS; see **Section S5.5**), 3 – 4 crystal layers could be expected.

(ii) *Extracting the thickness of the hexagonal disks by form factor fitting.* The procedure of fitting the experimental data to a cylindrical form factor model was previously outlined in **Section S5.4** and yielded an estimated thickness of 21 nm. Although slightly smaller compared to the dimensions directly from cryo-TEM, approximately 3 crystal layers can again be accommodated in the measured thickness.

(iii) *Counting the number of dsDNA per layer*. High resolution cryo-TEM images allow to distinguish individual dsDNA fragments. These fragments were manually assigned in three representative particles (**Figure S22**). Lattice positions that could be assigned with high confidence are labeled blue. Sites where the presence of a dsDNA was less clear are tagged red. Because the particles are hardly imaged fully face-on, the blurry, out-of-focus edges were not considered for the counting. On average 113 dsDNA fragments per layer were found. Comparing this number to the DNA aggregation number determined from the particle's molecular weight (see **Section S5.6**; N_{DNA} = 234 ± 10) yields approximately 2 crystal layers.

Although the number of layers is not perfectly consistent, they offer similar values. We anticipate that Method (***i***) and (***ii***) might slightly overestimate the number of layers due to the presence of interstitial spaces between the crystal layer to, *e.g.*, provide space for the charge-neutral segments of the BCPs.

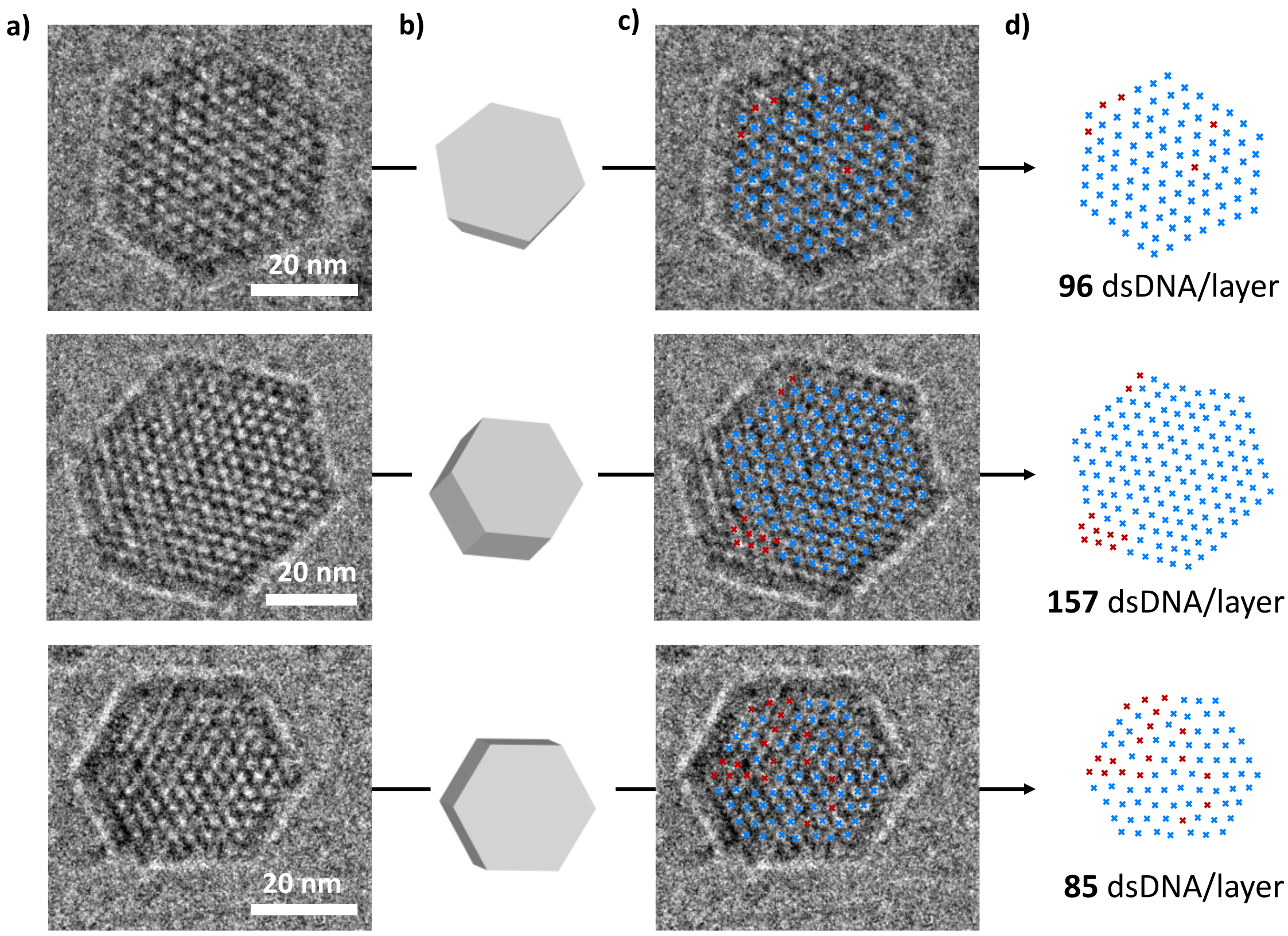

Figure S22. a) Representative cryogenic transmission electron microscopy (cryo-TEM) images of hexagonal disk-shaped particles obtained by PIESA. **b)** Assumed 3-dimensional orientation of the imaged particles. Blurry sections on the particle are assumed to originate from an out-of-focus side view. These regions were excluded when counting individual double-stranded DNA (dsDNA) strands as depicted in panel **c)**. The dsDNA strands appear as white dots. Dots marked with a blue × are assigned with high confidence, while a red × represents lattice site with a high uncertainty. Assignment was performed manually after increasing image contrast. **d)** Counting the total number of ×'s per image provides an estimation for the number of dsDNA per layer.

S5.8 Fraction of states calculations and verification. Given the existence of an iso-scattering point in the PIESA SAXS profiles, the fractions of states (F) were calculated using[18]

$$I(q,t) = F_{\text{State 1}}(t)\, I_{\text{start}}(q) + F_{\text{State 2}}(t)\, I_{\text{final}}(q) \qquad \textbf{Eq. S16a}$$

with

$$F_{\text{State 2}}(t) = 1 - F_{\text{State 1}}(t) \qquad \textbf{Eq. S16b}$$

To confirm that the transition between the two states was occurring on length scales that were adequate to the nanoparticle- and DNA/polymer size, two q-ranges were selected on both sides of the iso-scattering point: 0.046 – 0.15 nm^{-1} and 0.45 – 0.53 nm^{-1}. The fraction of state was calculated for each datapoint within the selected range for a SAXS profile at a given time point and averaged to give the final value for $F_{\text{State 1}}$ and $F_{\text{State 2}}$. The results are plotted in **Figure 3c** of the **Main Text**. The blue circles in **Figure S23a** contain the same $F_{\text{State 2}}$ values (error bars omitted for clarity).

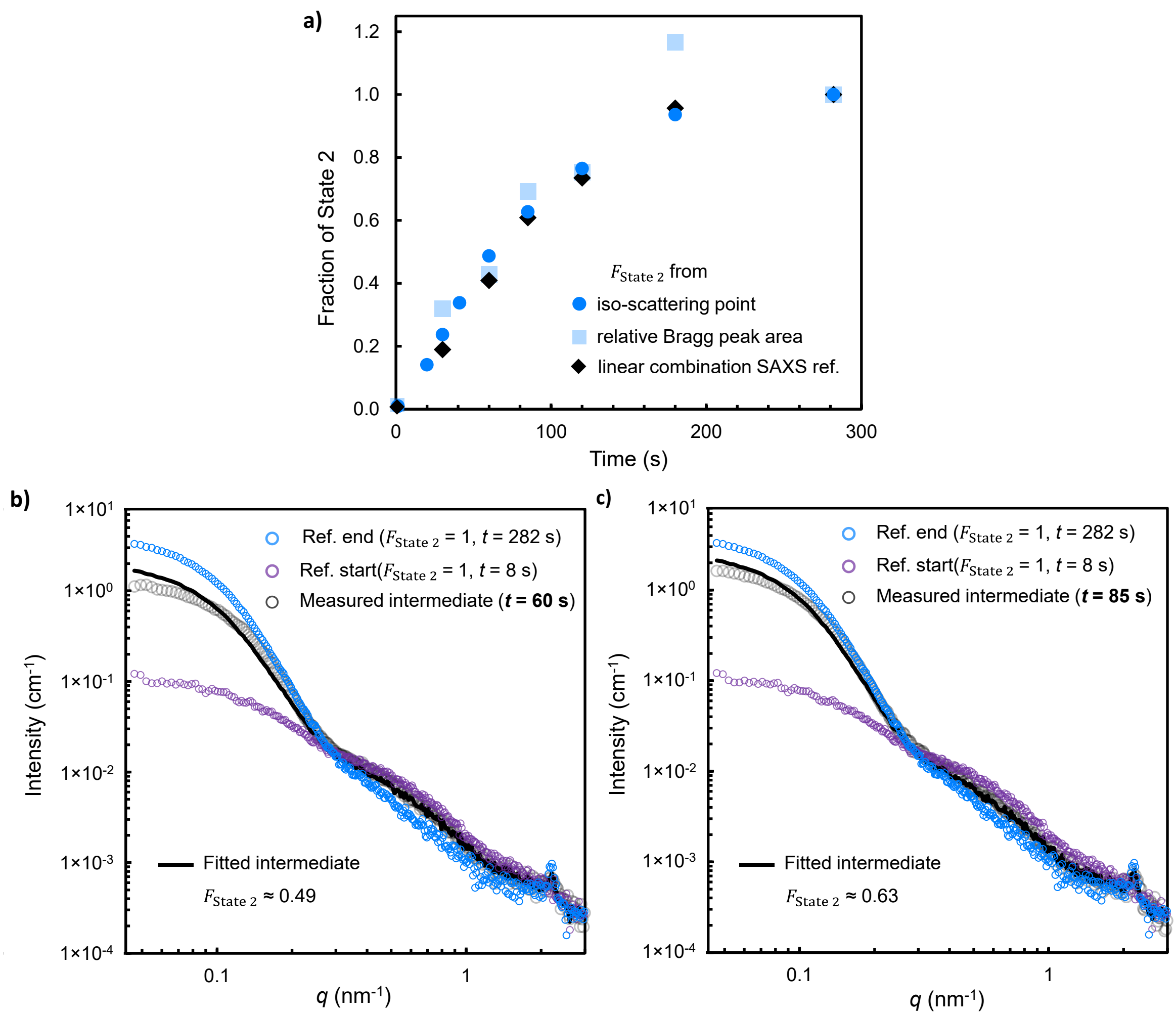

Figure S23. a) Fractions of block copolymers (BCPs) and double-stranded DNA (dsDNA) that reside in the particular state ($F_{State\ 2}$). $F_{State\ 2}$ was determined by using three independent analytical approaches based on the existence of an iso-scattering point (circles), the area under the Bragg peak (squares), and fitting intermediate SAXS frames as a linear combination of two reference SAXS profile representing the start- and end point of the assembly process (diamonds). All cases yield a comparable and gradual increase in $F_{State\ 2}$. **b), c)** Representative examples of fitting SAXS frames at intermediate times (t = 60 s and 85 s). The resulting fit (black line) is overlayed with the experimental data (gray). The reference spectra for the assembly end- and start point are shown in blue and purple, respectively.

To validate the analysis based on the iso-scattering point, an alternative way of determining the $F_{\text{State 2}}$ (particle state) was performed. Here, we propose that the total crystallinity in the sample at a given time point quantifies the total amount of dsDNA and BCP that reside in the particle phase. The area under the Bragg peak (**Figure S15**) provides a direct measure for the total crystallinity within the sample. By normalizing the area under the Bragg peak at a given time point by the maximum Bragg peak area obtained in the final SAXS frame, $F_{\text{State 2}}$ can be readily estimated (**Figure S23a**, light blue squares). The trend that $F_{\text{State 2}}$ gradually increases during the PIESA reaction is observed for both the iso-scattering and Bragg peak area-based analyses methods and the obtained values of $F_{\text{State 2}}$ are in good agreement. The observation that both analytical methods yield similar results must imply that dsDNA ordering is present throughout

the particle formation and growth. Furthermore, this implies that crystallite formation is not preceded by an amorphous state, as the Bragg peak only reflects ordered dsDNA strands. The relatively slow increase of R_g in time is consistent with the requirement that all incoming BCP–dsDNA complexes must occupy lattice positions within the growing particle (**Figure S17**).

A second alternative approach was used to estimate the relative contribution of two structural states during the transition. In this analysis, we assume that each recorded SAXS frame can be considered as a linear combination of two reference scattering profiles, representing the starting and final state. Building on the hypothesis of small unit charge-neutralized complexes (uPICs) that serve a building blocks for the particle assembly (see **Figure 4c** in **Main Text**), we selected the final frame (t = 282 s) as a reference for the particular state ($F_{\text{State 2}}$ = 1, **Figure S23b, c; blue**) and the last recorded frame before a distinct peak was observed in the Kratky plot (t = 8 s, see **Figure 3d** in **Main Text, Figure S23b, c**; **purple**) as reference for the uPIC state. Note that for this approximation, particle growth after nucleation was neglected. We speculate that this point just preceding particle nucleation represents the strongest scattering signature of the uPICs and equals $F_{state\,1}$ = 1. The optimal value of $F_{state\,2}$ is obtained by minimizing the difference between the target intermediate SAXS profile and the calculated mixed profile with $F_{state\,2}$ being constraint between 0 and 1. The fitting is performed in logarithmic intensity space so that both high and low intensity regions of SAXS data contribute evenly to the fit. The sum of squared errors (SSE) was calculated as

$$\text{SSE} = \sum_{q} [\log(I_{\text{intermediate}}(q)) - \log(I_{\text{mix}}(q))]^2 \qquad \textbf{Eq. S17}$$

where $I_{\text{intermediate}}(q)$ is the measured SAXS profile of the intermediate frame and $I_{\text{mix}}(q)$ is the calculated mixed two-state profile. Only positive intensity values were included in the logarithmic fit. The optimization was performed in Python using a bounded scalar minimization algorithm. $F_{\text{State 1}}$ and $F_{\text{State 2}} = 1 - F_{\text{State 1}}$ were extracted after optimizing SSE. respectively. Representative optimized mixed profiles are plotted in **Figure S23b, c** for SAXS frames recorded after 60 and 85 s. Despite a slight mismatch in the fit, the extracted values of $F_{\text{State 2}}$ (**Figure S23a**, diamonds) match the values obtained from the iso-scattering and Bragg peak area analysis. The inability to fully capture the shape of the experimental profile can be attributed to imperfection in the reference profiles and ignoring particle growth that occurs after rapid nucleation (see **S5.10** and FCS data in **Figure 4** of the **Main Text**). The reference for the end point can still contain minor contributions of molecularly dissolved macromolecules. Likewise, the reference spectrum for the starting point does not exclusively contain uPICs because of the temporally spaced-out formation of these species (see **Figure 4** in the **Main Text**). Additionally, a minor fraction of intermediate species not strictly belonging to **State 1** or **2** might be present.

S5.9 Direct mixing: Kratky Plot.

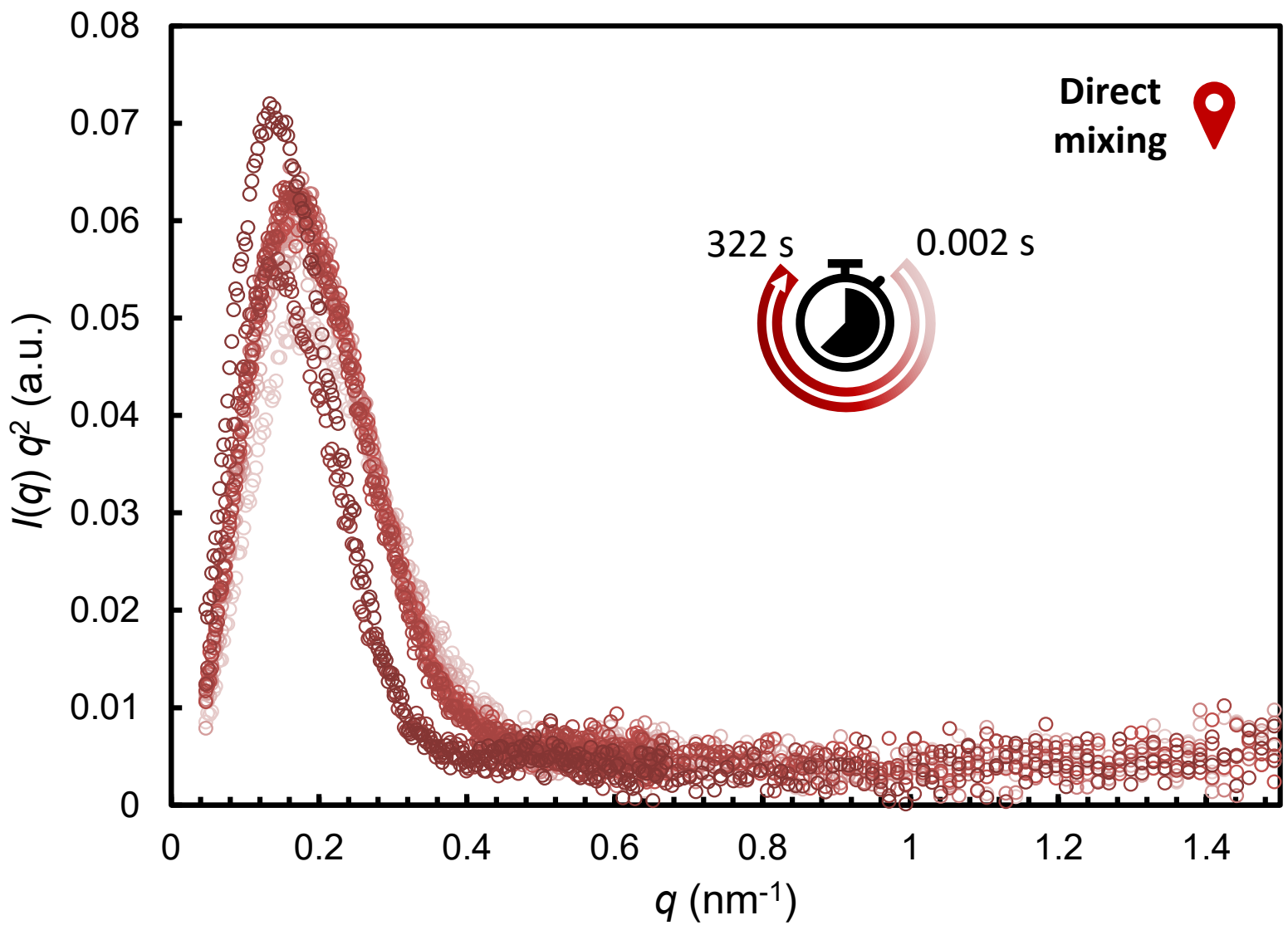

Figure S24. Time evolution of the Kratky plots during a direct mixing experiment probed with time-resolved small-angle X-ray scattering (TR-SAXS). After the 2 ms deadtime used for mixing the solutions of encapsulating block copolymer and the double-stranded DNA (dsDNA), a distinct peak was already observed indicating extremely rapid particle formation. After initial particle formation, the Kratky plots show minor signs of structural rearrangements.

S5.10 Simulated SAXS curves of toy models.

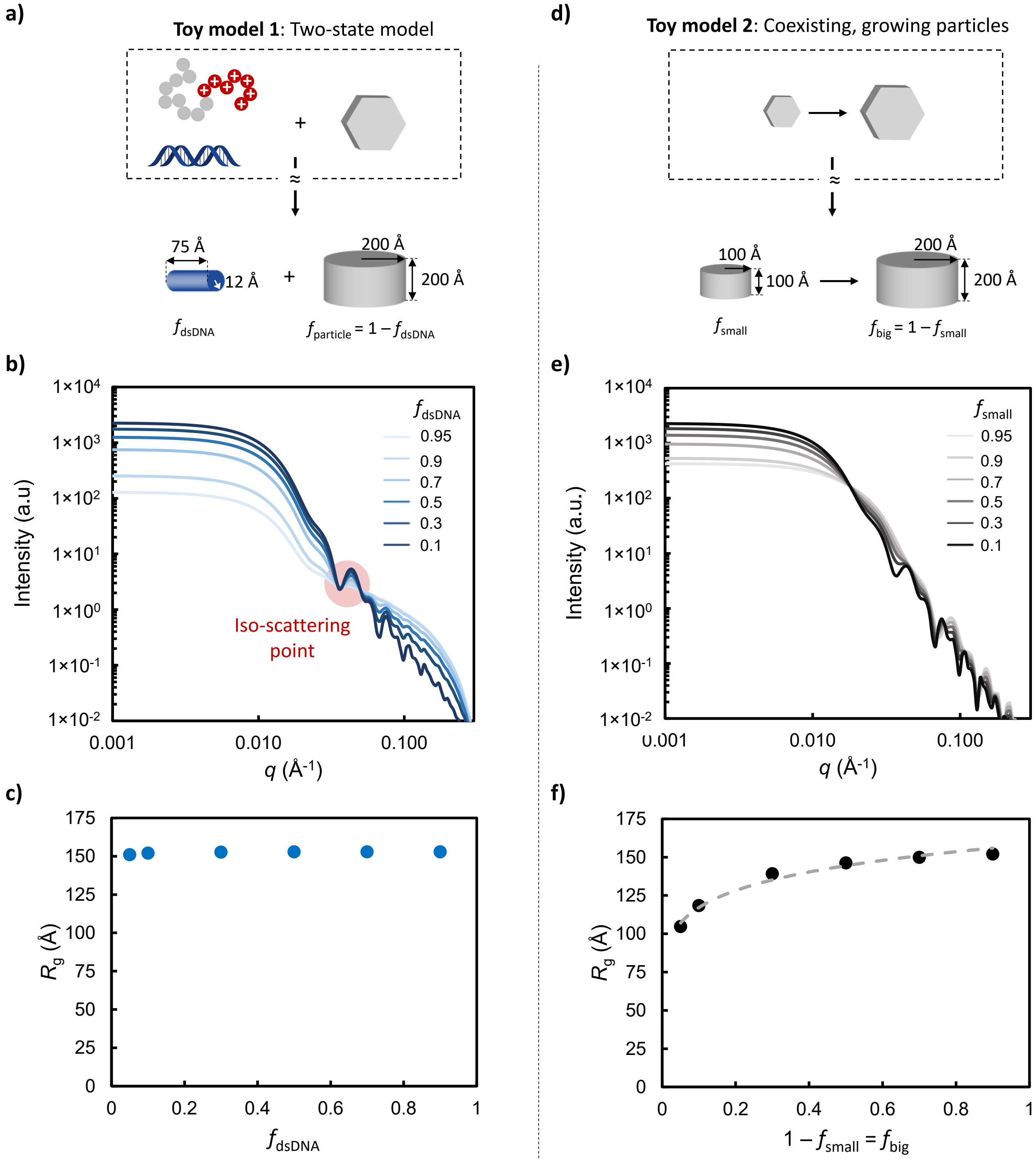

Figure S25. a) Schematic representation of the toy model representing the coexistence of **State 1** (non-encapsulated dsDNA) and **State 2** (encapsulated dsDNA in nanoparticles) (see **Eq. S16**). Only free dsDNA and nanoparticles are considered due to lower scattering contrast of the polymer. Both dsDNA (blue) and the nanoparticles (gray) are approximated as stiff cylinders. **b)** Calculated SAXS profiles for a mixture containing dsDNA and nanoparticles as a function of their relative fractions (f_{dsDNA} and $f_{particle}$). The observed iso-scattering point is highlighted in red. **c)** Radius of gyration (R_g) extracted from the profiles in panel b) using the Guinier analysis (see **Section S5.2**). **d)** Schematic representation of the toy model representing the growth of the PIESA-derived particles by having two coexisting disk-shaped particles with different dimensions. The mixing ratio of both disk-shaped particles is varied to simulate the growth. **e)** Calculated SAXS profiles as a function of the fraction of small disks. **f)** R_g values extracted from the profiles in panel e) using the Guinier analysis. The dotted line represents a power-law fit to the data points.

To further emphasize the mechanistic underpinnings of the PIESA process discussed in the **Main Text**, the general features of the experimental PIESA-derived TR-SAXS profiles (**Main Text**, **Figure 2b, d**) were simulated using two toy models.

S5.10.1 Iso-scattering point. The first model (**Figure S25a**) attempts to capture the iso-scattering point by assuming that the observed scattering at any given time is determined by the presence of non-encapsulated dsDNA and particles with a fixed dimension (disk with radius and height of 200 Å). Therefore, particle growth was not included in this model. The scattering signature of the (growing) block copolymer (BCP) was neglected due to low contrast with the medium. Both dsDNA and the particles were for simplicity modeled as hard cylinders (see **Section S5.4** for form factor description). This assumption is justified, as the final frame of the PIESA-derived TR-SAXS profile and the scattering profile measured for a pure solution of dsDNA, could be fitted reasonably well with cylindrical form factors (see **Figure S20** and **S21**). To simulate the PIESA process, the relative fraction of free (f_{dsDNA}) and encapsulated dsDNA ($f_{\text{particle}} = 1 - f_{\text{dsDNA}}$) was varied. Summing up the scattering contributions of both species at these varying fractions yields the profiles plotted in **Figure S25b**. In agreement with the experimental data, we observe an increase in scattered intensity (here in a.u.) at $q \rightarrow 0$, corresponding to the growing contribution of particle scattering. At $q \approx 0.035$ Å^{-1} the iso-scattering point emerged (**Figure S25b**, highlighted in red), which is remarkably close to the experimentally determined value ($q \approx 0.29$ nm^{-1} = 0.035 Å^{-1}). Note that the sharp oscillations at high q are the result of ignoring any polydispersity in the form factor of both dsDNA and the particle species. Extracting the radius of gyration (R_g) from the simulated scattering profiles using the Guinier analysis (see **Section S5.2**), yields a constant value of approximately 150 Å. The result is not surprising as this toy model assumes no particle growth. Evidently, this does not match the experimentally observed evolution in R_g and implies the particles are growing during the PIESA process (as suggested by the FCS data; **Figure 4b** in **Main Text**).

S5.10.2 Particle growth & R_g evolution. The second model explicitly takes the particle growth phase (as observed with FCS measurements) during PIESA into account (**Figure S25d**). For simplicity, the growth was modeled as a coexistence between smaller and larger particles, where the fraction of the larger particles was increased. As for the first toy model, the PIESA-derived particles were approximated as hard cylinders with a

radius and thickness of 100 and 200 Å for the small and big particles, respectively. The composite scattering profiles as a function of the relative particle fractions (f_{small}, f_{big}) are shown in **Figure S25e**. Note that these profiles also show an iso-scattering point at $q \approx 0.018$ Å^{-1}, due to simplifying the growth process by assuming the coexistence of only two particle species. A more realistic scenario is that particle growth is a continuous process, leading to the instantaneous coexistence of many different particle sizes. This would avoid the formation of an iso-scattering point and result in gradual shifts of the profile's oscillations. Regardless of this simplification, plotting R_g as function of f_{big} (**Figure S25f**) reveals a characteristic power law evolution (dotted line = power law fit: $R_g \sim f_{big}^{0.13}$) also observed experimentally (see **Figure 3e** in **Main Text**). This striking similarity shows that particle growth is likely occurring during a PIESA process and that **State 2** (as defined in the **Main Text**, **Eq. 1**) should be interpreted indeed as a fraction of encapsulated dsDNA rather than a static particle state with fixed dimensions.

S6. References

[1] M. Lueckheide, J. R. Vieregg, A. J. Bologna, L. Leon, M. V. Tirrell. Structure-Property Relationships of Oligonucleotide Polyelectrolyte Complex Micelles. *Nano Lett.* **2018**, *18* (11), 7111–7117. DOI: https://doi.org/10.1021/acs.nanolett.8b03132

[2] F. H. Sobotta, B. G. P. van Ravensteijn, I. K. Voets. Sequence-Controlled Neutral-Ionic Multiblock-Like Copolymers through Switchable PIESA in a One-Pot Approach. *ACS Macro Lett.* **2025**, *14* (9), 1277–1283. DOI: https://doi.org/10.1021/acsmacrolett.5c00108

[3] P. Sengupta, K. Garai, J. Balaji, N. Periasamy, S. Maiti. Measuring Size Distribution in Highly Heterogeneous Systems with Fluorescence Correlation Spectroscopy. *Biophys. J.* **2003**, *84* (3), 1977–1984. DOI: https://doi.org/10.1016/S0006-3495(03)75006-1

[4] A. Guinier, G. Fournet, *Small-Angle Scattering of X-rays*, John Wiley & Sons, London, **1955**.

[5] http://www.sasview.org/,

[6] L. S. van Hazendonk, R. Tuinier, E. Foschino, L. Matthews, H. Friedrich, M. Vis. Morphological Analysis of Polydisperse Nanoplatelets using SAXS. *Colloids Surf. A: Physicochem. Eng. Asp.* **2024**, *702*. DOI: https://doi.org/10.1016/j.colsurfa.2024.134997

[7] D. C. Arruda, I. J. Gonzalez, S. Finet, L. Cordova, V. Trichet, G. F. Andrade, C. Hoffmann, P. Bigey, W. A. de Almeida Macedo, A. Da Silva Cunha, Jr., A. Malachias de Souza, V. Escriou. Modifying Internal Organization and Surface Morphology of siRNA Lipoplexes by Sodium Alginate Addition for Efficient siRNA Delivery. *J. Colloid Interface Sci.* **2019**, *540*, 342–353. DOI: https://doi.org/10.1016/j.jcis.2019.01.043

[8] M. D. Krebs, E. Alsberg. Localized, targeted, and sustained siRNA delivery. *Chem. Eur. J.* **2011**, *17* (11), 3054–3062. DOI: https://doi.org/10.1002/chem.201003144

[9] K. M. Hansen, H. F. Ji, G. Wu, R. Datar, R. Cote, A. Majumdar, T. Thundat. Cantilever-Based Optical Deflection Assay for Discrimination of DNA Single-Nucleotide Mismatches. *Anal. Chem.* **2001**, *73* (7), 1567–1571. DOI: https://doi.org/10.1007/978-981-13-6004-6_3

[10] N. Gupta, D. B. Rai, A. K. Jangid, D. Pooja, H. Kulhari, in *Nanotechnology in Modern Animal Biotechnology: Recent Trends and Future Perspectives* (Eds.: S. Singh, P. K. Maurya), Springer Singapore, Singapore, **2019**, pp. 67–114.

[11] A. Schroeder, C. G. Levins, C. Cortez, R. Langer, D. G. Anderson. Lipid-Based Nanotherapeutics for siRNA Delivery. *J. Intern. Med.* **2010**, *267* (1), 9–21. DOI: https://doi.org/10.1111/j.1365-2796.2009.02189.x

[12] I. K. Voets, S. van der Burgh, B. Farago, R. Fokkink, D. Kovacevic, T. Hellweg, A. de Keizer, M. A. C. Stuart. Electrostatically Driven Coassembly of a Diblock Copolymer and an Oppositely Charged Homopolymer in Aqueous Solutions. *Macromolecules* **2007**, *40* (23), 8476–8482. DOI: https://doi.org/10.1021/ma071356z

[13] M. A. H. Farmer, O. O. Mykhaylyk, O. Shebanova, O. M. Musa, S. P. Armes. Time-Resolved SAXS Studies During the Synthesis of Hydrolytically Degradable Poly(ε-caprolactone)-Poly(*N*,*N*'-dimethylacrylamide) Diblock Copolymer Nanoparticles in Aqueous Media. *Angew. Chem. Int. Ed. Engl.* **2026**, *65* (2), e24000. DOI: https://doi.org/10.1002/anie.202524000

[14] C. Wohlfarth, *Physical Properties and their Relations I (Thermodynamic Properties: pVT data and miscellaneous properties of polymer solutions)*, Springer Materials.

[15] E. Read, B. Lonetti, S. Gineste, A. T. Sutton, E. Di Cola, P. Castignolles, M. Gaborieau, A. F. Mingotaud, M. Destarac, J. D. Marty. Mechanistic Insights into the Formation of Polyion Complex Aggregates from Cationic Thermoresponsive Diblock Copolymers. *J. Colloid Interface Sci.* **2021**, *590*, 268–276. DOI: https://doi.org/10.1016/j.jcis.2021.01.028

[16] C. Wandrey, A. Bartkowiak, D. Hunkeler. Partial Molar and Specific Volumes of Polyelectrolytes: Comparison of Experimental and Predicted Values in Salt-free Solutions. *Langmuir* **1999**, *15* (12), 4062–4068. DOI: https://doi.org/10.1021/la981089d

[17] G. F. Bonifacio, T. Brown, G. L. Conn, A. N. Lane. Comparison of the Electrophoretic and Hydrodynamic Properties of DNA and RNA Oligonucleotide Duplexes. *Biophys. J.* **1997**, *73* (3), 1532–1538. DOI: https://doi.org/10.1016/S0006-3495(97)78185-2

[18] M. T. Alting, D. M. E. Thies-Weesie, A. M. van Silfhout, M. de Ruiter, T. Narayanan, M. F. Haase, A. V. Petukhov. Insights into Formation of Bicontinuous Emulsion Gels via In Situ (Ultra-)Small Angle X-Ray Scattering. *J. Phys. Chem. B* **2025**, *129* (25), 6419–6427. DOI: https://doi.org/10.1021/acs.jpcb.5c02375
